# Phospholipase D1 activates NAD kinase fuelling neutrophil extracellular trap formation

**DOI:** 10.1038/s41467-026-79319-6

**Published:** 2026-11-06

**Authors:** Rahaf Albarghash, Sergio G. San-Miguel, Arturo Zychlinsky, Dhiren Ferise Patel

**Affiliations:** Department of Cellular Microbiology, Max Planck Institute for Infection Biology, Berlin, Germany

**Keywords:** Neutrophils, Phospholipids, Lipidomics, Innate immunity

## Abstract

Neutrophil extracellular trap formation is a central innate immune defence mechanism that depends on NADPH oxidase–driven reactive oxygen species production. How NADP⁺ availability is regulated during neutrophil activation remains unclear. Using integrated lipidomic, biochemical and functional analyses in human neutrophils, we identify NAD kinase, the sole enzyme responsible for NADP⁺ synthesis, as a direct lipid-regulated effector downstream of phospholipase D1. Neutrophil activation induces rapid, phospholipase D1-dependent production of phosphatidic acid and phosphatidylinositol 4,5-bisphosphate, which bind and activate NAD kinase. The resulting cytosolic NADP⁺ is reduced to NADPH by the oxidative pentose phosphate pathway and consumed by NADPH oxidase during neutrophil extracellular trap formation. Pharmacological or genetic disruption of phospholipase D1 suppresses NAD kinase activity, depletes NADP⁺ and impairs neutrophil extracellular trap formation independently of direct NADPH oxidase inhibition. These findings define a lipid–metabolic axis coupling phospholipase D1-driven membrane remodelling to NADP⁺ availability and innate immune function.

## Abstract (web summary)

Neutrophil extracellular trap (NET) formation is a key innate response that relies on NADPH oxidase-driven production of reactive oxygen species. Here, the authors implicate phospholipase D1 in the activation of NAD kinase linked to NET formation.

## Introduction

Neutrophils are the most abundant circulating leukocytes and are essential first responders to microbial invasion^1^. Upon activation, they rapidly remodel metabolic and signalling networks to migrate toward sites of infection and deploy antimicrobial mechanisms^1,2^. These include phagocytosis, degranulation, production of reactive oxygen species (ROS), and the release of neutrophil extracellular traps (NETs)^1,3,4^. NETs are web-like structures composed of decondensed chromatin coated with antimicrobial proteins that trap and kill pathogens^1,3^. Individuals unable to form NETs experience recurrent infections^1^. Conversely, dysregulated or excessive NETosis contributes to inflammation, tissue damage, and autoimmunity^1^.

NET formation triggered by microbial or mitogenic stimuli critically depends on ROS generated by the NADPH oxidase (NOX2) complex^1,5^. Sustained ROS production requires continuous regeneration of NADPH from NADP^+^, principally via the oxidative pentose phosphate pathway (PPP)^1,2^. This places NADP^+^ availability at the core of the metabolic control of NETosis, yet the upstream mechanisms that regulate NADP^+^ synthesis in cell biology, let alone in activated neutrophils, remain poorly defined.

Lipids represent one of the most versatile signalling modules in immune cells^6,7^. Beyond their structural roles, phospholipids undergo rapid stimulus-dependent remodelling and directly regulate protein localisation, conformation and activity through electrostatic or hydrophobic interactions^6–9^. Such lipid–protein interactions shape signalling outputs, metabolic processes and cell activation pathways. Among these phospholipid species, phosphatidic acid (PA) is a rapidly inducible lipid implicated in membrane remodelling and inflammatory signalling in immune cells^10,11^, yet its role in metabolic control during neutrophil activation remains poorly defined. Despite these broad regulatory capabilities, how phospholipids regulate the metabolic and oxidative requirements of NETosis remains unclear. Understanding whether specific lipid pathways support NADPH production, ROS generation, or downstream NET release could reveal how neutrophil effector function is regulated.

Here, we address this question by integrating lipidomic, biochemical, and functional analyses of human neutrophils undergoing activation and NET formation. We find that stimulation triggers extensive lipid remodelling, marked by a pronounced increase in PA and the phosphoinositide PI(4,5)P₂ (PIP₂). We identify phospholipase D1 (PLD1) as the central orchestrator of this response, controlling the production of PA. PLD1-derived PA also enhances downstream PIP₂ synthesis by promoting the activity of the phosphatidylinositol-4-phosphate 5-kinase (PIP5K1), positioning PLD1 as a broad remodeller of the neutrophil lipidome. Mechanistically, we show that PLD1-derived PA and PIP₂ directly bind to and activate NAD kinase (NADK)—the sole enzyme converting NAD^+^ into NADP^+^, thereby rapidly increasing NADP^+^ availability, which is reduced to NADPH via the oxidative PPP and consumed by NOX2 during NET formation. Together, these findings establish PLD1 as a key regulator of lipid remodelling and uncover a lipid–enzyme regulatory axis that couples membrane signalling to NADP^+^ availability, ROS generation and NET formation.

## Results

### PA, formed by phospholipase D1, is required for NET formation

**Fig. 1:**
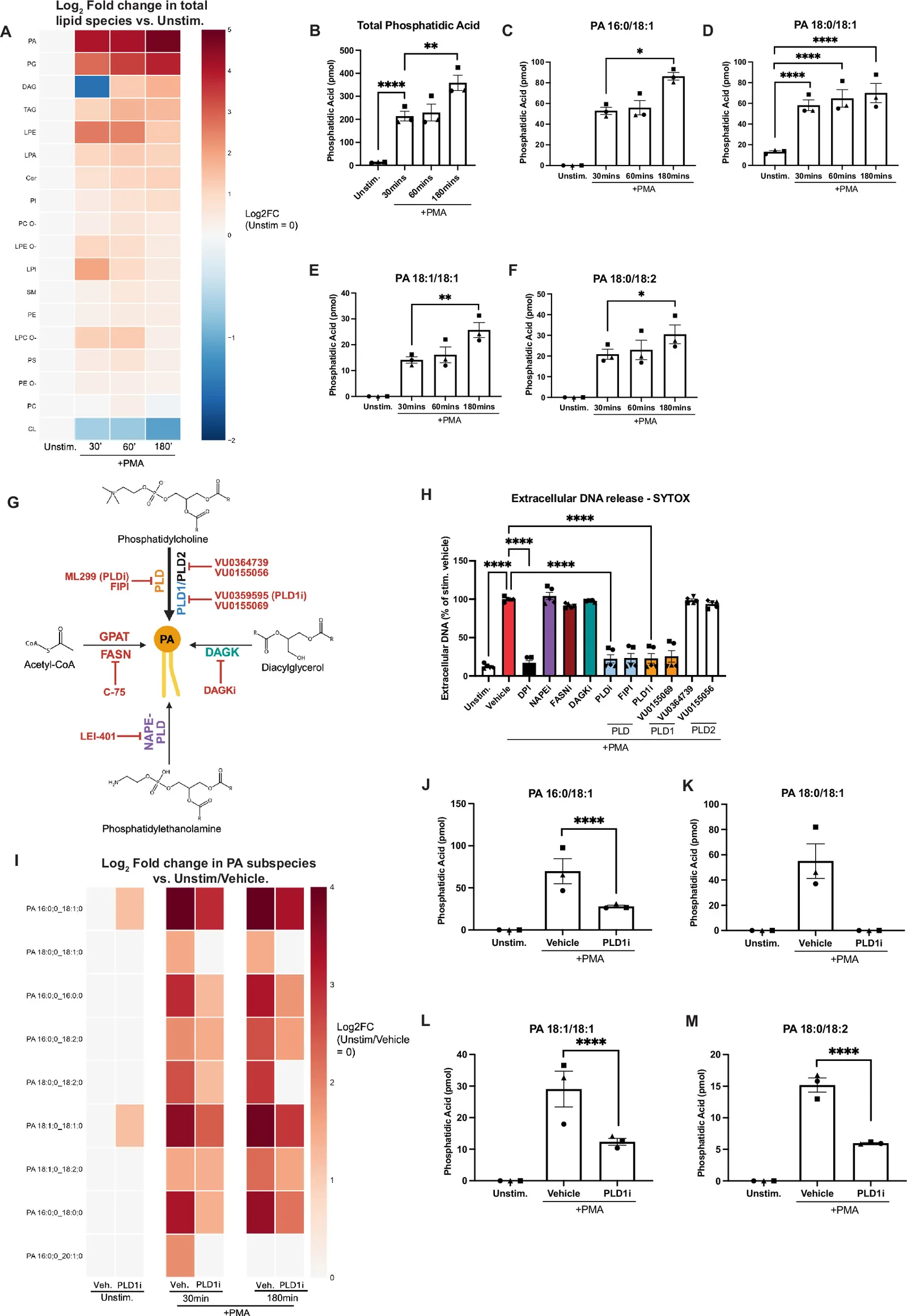
PLD1 synthesises PA rapidly during NETosis. **A** Heatmap showing log₂ fold change (log₂FC) in major lipid classes in lysates of human neutrophils stimulated with PMA for 30, 60 or 180 min compared to unstimulated cells, including phosphatidylglycerol (PG), diacylglycerol (DAG), triacylglycerol (TAG), lysophospholipids (LPE, LPA), ceramides (Cer), phosphatidylinositol (PI), and cardiolipin (CL). Data shown as mean log₂ fold change, *N* = 3 donors. **B** Total PA concentration in neutrophil lysates measured by shotgun lipidomics. **C** PA 16:0/18:1, **D** PA 18:0/18:1, **E** PA 18:1/18:1, and (**F**) PA 18:0/18:2, the four major PA species at 180 min. **G** Schematic of PA synthesis pathways and the inhibitors used for the indicated enzymes. Created in BioRender. Patel, D. (2026) https://BioRender.com/joo2wz1. **H** Extracellular DNA release, used here as a proxy for NETs, was measured fluorometrically with the cell-impermeable dye, SYTOX Green. Neutrophils were pre-incubated with the indicated inhibitors for 30 min before PMA stimulation. **I** Heatmap showing log₂ fold change in PA subspecies in lysates of neutrophils pre-incubated with vehicle or the PLD1 inhibitor (PLD1i) and stimulated with PMA for 30 or 180 min, normalised to unstimulated vehicle-treated neutrophils. J–M PA subspecies concentrations in lysates of neutrophils stimulated with PMA for 30 min with or without PLD1i. **J** PA 16:0/18:1, **K** PA 18:0/18:1, **L** PA 18:1/18:1 and (**M**) PA 18:0/18:2. **B**–**F**, **J**–**M** Data shown as mean ± s.e.m., *N* = 3 donors. Each point is one donor, plotted as the mean of 3 (**B**–**F**) or 2 (**J**–**M**) technical replicates and shown with a different symbol. **H** Data shown as mean ± s.e.m., *N* = 5 donors. Each point is one donor, plotted as the mean of 2 technical replicates and shown with a different symbol. **B**–**F**, **J**–**M** Statistical significance was determined by moderated *t*-tests (limma) with Benjamini–Hochberg correction for multiple comparisons. **H** Statistical significance was determined by one-way ANOVA with Bonferroni’s multiple comparisons test. *P < 0.05, **P < 0.01 and ****P < 0.0001. Species not detected are shown as 0 and excluded from statistical comparisons. Exact *P* values are provided in the Source Data file. Source data are provided as a Source Data file.

To identify the lipid pathways regulated during NETosis, we stimulated neutrophils with the mitogen, Phorbol 12-myristate 13-acetate (PMA) for 30, 60, or 180 min and analysed their lysates by mass spectrometry (Supplementary Fig. 1A). Although PA is only 0.1 % of the naive neutrophil lipidome (Supplementary Fig. 1B–E), it increased more than 10-fold 30 min after PMA stimulation (Fig. 1A, B). Other lipid species, including phosphatidylglycerol (PG), diacylglycerol (DAG), triacylglycerol (TAG), lysophosphatidylethanolamine (LPE), lysophosphatidic acid (LPA), ceramides (Cer) and phosphatidylinositol (PI) also increased, albeit to a lesser degree (Fig. 1A).

PA subspecies, like other phospholipids, vary based on the composition of their 2 fatty acyl chains^12^. Numerous PA subspecies were increased by PMA stimulation. We focused on the four most abundant and robustly induced PA subspecies at 30 and 180 min: PA (16:0/18:1), PA (18:0/18:1), PA (18:1/18:1) and PA (18:0/18:2), highlighted in Fig. 1C–F.

PA in mammalian cells is generated by four defined enzymes, phospholipase D (PLD), DAG kinase (DAGK), N-acyl phosphatidylethanolamine-specific phospholipase D (NAPE-PLD) and glycerol-3-acyltransferases (GPATs) (Fig. 1G). Although we did not directly inhibit GPATs, we used an inhibitor of fatty acid synthase (FASN), since FASN supplies the fatty acids that GPATs use to synthesise PA. Notably, PLD, but not DAGK, FASN, or NAPE-PLD, inhibitors significantly blocked PMA-induced NETosis (Fig. 1H). In this experiment, DPI (diphenyleneiodonium, a NOX2 inhibitor) served as a positive control.

PLD converts phosphatidylcholine (PC) to PA by replacing the choline headgroup with a monophosphate^10,12^ (Fig. 1G). Of the six mammalian PLD isoforms, only PLD1 and PLD2 synthesise PA (Fig. 1G). Targeting each isoform with two inhibitors (Fig. 1G) for PLD1 (VU0359595, hereafter called PLD1i, and VU0155069) and PLD2 (VU0364739 and VU0155056), revealed that PLD1, but not PLD2, is required for NET formation induced by PMA, a direct protein kinase C (PKC) activator (Fig. 1H). PLD1 inhibition also blocked NET formation triggered by two distinct NOX2-dependent stimuli, the calcium ionophore A23187 and heat-killed *Candida albicans* hyphae (a physiological, receptor-mediated stimulus)^5^ (Supplementary Fig. 1F–G). We confirmed the inhibitor results with live microscopy (Supplementary Movies 1–5) and with immunofluorescent staining (IF) (Supplementary Fig. 2A–C) of neutrophils activated for 4 hours with PMA. We detected NETs using the NET-specific antibody 3D9, which recognises cleaved histone H3^13^, alongside a panel of control inhibitors, Neutrophil Elastase inhibitor (NEi, BAY 85-8501), DPI and the pan-isoform PKC inhibitor, Gö6983 (PKCglo) to validate the pathway specificity (Supplementary Fig. 2).

To elucidate the effects of PLD1 on PA production, we compared the lipidome of neutrophils treated with PLD1i before stimulation with PMA. A heatmap showing fold changes in PA subspecies relative to unstimulated vehicle-treated neutrophils showed that PMA stimulation led to an induction of nearly all detectable PA subspecies (Fig. 1I). More importantly, PLD1i reduced PMA-induced PA subspecies (Fig. 1I). The four most highly induced PA subspecies (Fig. 1C–F) also required PLD1 for their production (Fig. 1J–M). Together, these results indicate that PLD1 is required for PA synthesis during NET formation.

Consistently, PLD activity increased upon PMA activation, as measured with a fluorogenic substrate (Supplementary Fig. 3A). PMA elicits PKC and NOX2 activity to trigger NETosis^14^. Notably, the PKC inhibitor PKCglo significantly reduced PLD activity. DPI had a statistically significant but modest effect on PLD activity compared with the PLD inhibitors, indicating that ROS contribution is minor (Supplementary Fig. 3A). A23187 and *C. albicans* hyphae^5^ also robustly induced neutrophil PLD activity largely independently of ROS production. As expected, ML299 (PLDi, PLD1 and PLD2 inhibitor) and PLD1i blocked increases in PLD activity by all stimuli (Supplementary Fig. 3A). Since PLD liberates choline from PC, as expected, the concentration of choline (Supplementary Fig. 3B) mirrored the readout for PLD activity (Supplementary Fig. 3A).

Upon cellular activation, PLD1 relocates to specific cellular membranes to produce PA locally^11^. In PMA-stimulated neutrophils, PLD1 redistributes from the plasma membrane to the cytoplasm 30 min after stimulation (Supplementary Fig. 3C, D). PLD1 intensity quantification confirmed that the cytoplasmic signal increased after PMA stimulation and was reduced by PLD1 and PKC inhibition (PKCglo), but not by DPI (Supplementary Fig. 3D). PLD1 was not detectable 180 min after stimulation, likely because endomembranes disassemble during NETosis^15^. Notably, PLD1 did not colocalise with NETs, indicating that its activity is intracellular prior to chromatin decondensation (Supplementary Fig. 3C).

### Phospholipase D1 promotes the formation of phosphoinositides

**Fig. 2:**
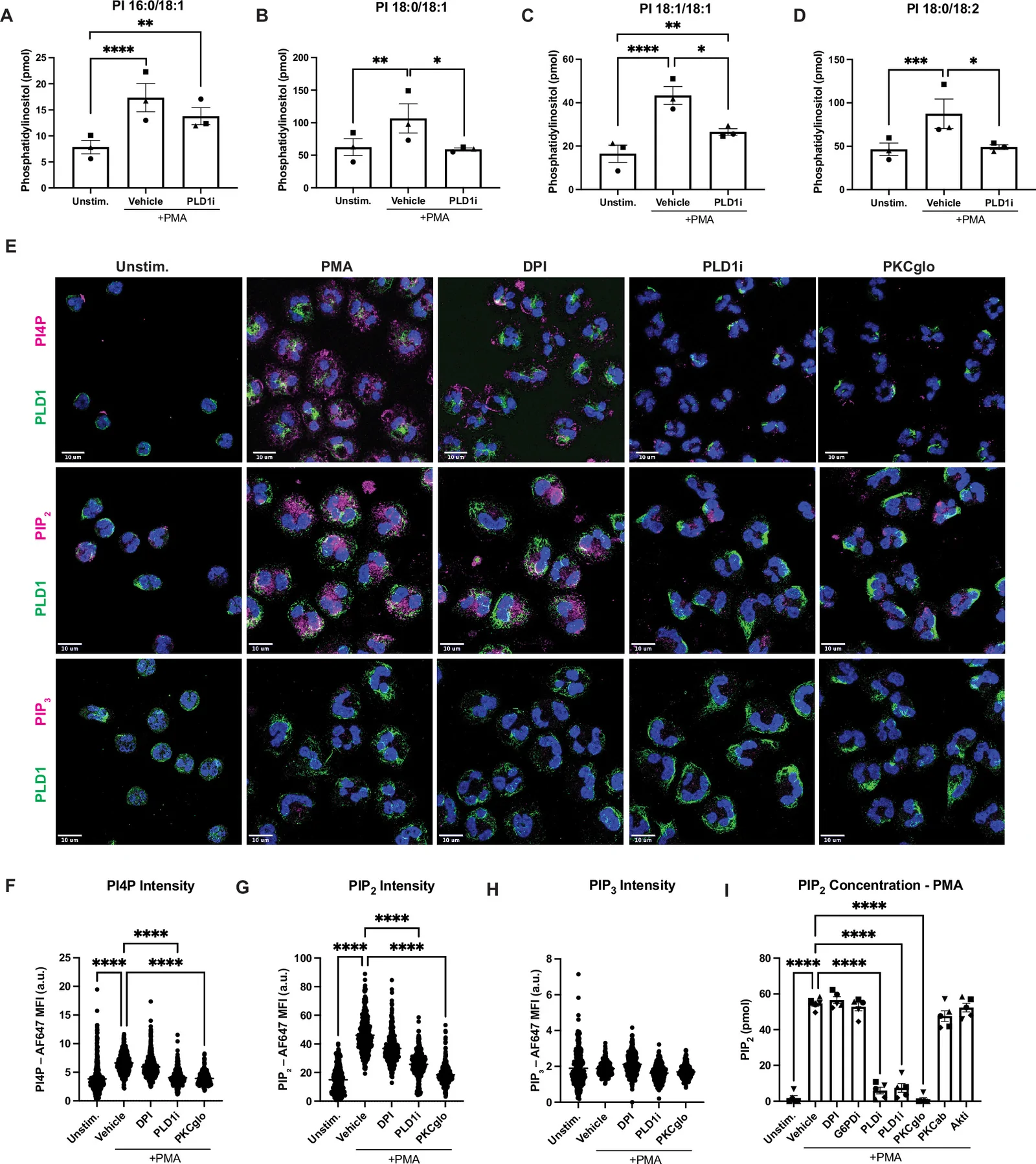
PLD1 promotes PI and PIP₂ synthesis during neutrophil activation. Concentrations of the indicated PI subspecies in lysates of neutrophils stimulated with PMA with or without PLD1i and measured by lipidomics: **A** PI 16:0/18:1, **B** PI 18:0/18:1, **C** PI 18:1/18:1 and (**D**) PI 18:0/18:2. **E** Representative confocal images of neutrophils pre-incubated with the indicated inhibitors before activation and stained with antibodies to PLD1 (green) and PI4P (magenta) top panels, PLD1 (green) and PIP₂ (magenta) middle panels, and PLD1 (green) and PIP₃ (magenta) bottom panels. Confocal images quantification of cells stained with antibodies recognising PI4P (**F**), PIP₂ (**G**) and PIP₃ (**H**). Mean fluorescence intensity of the Alexa 647-labelled secondary. **I** PIP₂ concentrations in lysates of neutrophils treated with the indicated inhibitors for 30 min before stimulating them with PMA. **A**–**D** Data shown as mean ± s.e.m., *N* = 3 donors. Each point is one donor, plotted as the mean of 2 technical replicates and shown with a different symbol. **E** Representative images from *N* = 2 donors. Scale bar, 10 µm. Data shown as mean ± s.e.m., *N* = 2 donors, each data point representative of individual cells (*n* = cells per condition, donor 1/donor 2; Unstim., Vehicle, DPI, PLD1i, PKCglo): **F** 211/265, 202/272, 202/298, 167/196, 112/111; **G**, 141/85, 229/209, 155/217, 164/121, 197/183; **H**, 185/74, 109/116, 149/165, 120/147, 87/134. **I** Data shown as mean ± s.e.m., *N* = 5 donors. Each point is one donor, plotted as the mean of 2 technical replicates and shown with a different symbol. **A**–**D** Statistical significance was determined by moderated t-tests (limma) with Benjamini–Hochberg correction for multiple comparisons. **F**–**G**, **I** Statistical significance was determined by one-way ANOVA with Bonferroni’s multiple comparisons test. *P < 0.05, **P < 0.01, ***P < 0.001 and ****P < 0.0001. Species not detected are shown as 0 and excluded from statistical comparisons. a.u., arbitrary units. Exact *P* values are provided in the Source Data file. Source data are provided as a Source Data file.

PA is a metabolic intermediate that contributes to the synthesis of multiple phospholipids and promotes the activity of lipid-modifying enzymes^10,12,16,17^. Consistently, PLD1 inhibition altered the broader neutrophil lipidome, reducing not only PA but also other lipid classes such as Cer, TAG, and phosphatidylinositol (PI) (Supplementary Fig. 4A). PI was of particular interest because its phosphorylated derivatives (phosphoinositides) regulate actin remodelling and the oxidative burst^18,19^, key processes in NETosis. Fold change analysis of PI subspecies in neutrophils exposed to PMA or vehicle for 30 min revealed that PLD1 inhibition blunted the induction of most detectable PI species (Supplementary Fig. 4B). Although PA and PI can interconvert through the cytidine diphosphate-diacylglycerol (CDP-DAG)/PI cycle^20^, we did not measure this flux directly. To look for any relationship, we examined PI subspecies whose fatty acyl chains matched the four major PA species identified earlier (Fig. 1C–F). While PLD1 inhibition did not decrease PI (16:0/18:1), it significantly reduced PI (18:0/18:1), PI (18:1/18:1), and PI (18:0/18:2) (Fig. 2A–D). These findings indicate that PLD1 activity shapes the PI subspecies profile during NETosis.

We next assessed downstream PI-derived phosphoinositides, phosphatidylinositol-4-monophosphate (PI4P), phosphatidylinositol 4,5-bisphosphate (PIP₂) and phosphatidylinositol (3,4,5)-trisphosphate (PIP₃) (Supplementary Fig. 3E)^16,17^. PMA triggered PI4P and PIP₂ accumulation in the cytoplasm and nucleoplasm, which was abrogated upon PLD1 inhibition (Fig. 2E). Quantification of the mean fluorescence intensity confirmed that PLD1i and PKCglo, but not DPI, reduced PI4P and PIP_2_ (Fig. 2F–G). PIP₃ gave a signal close to background by IF, consistent with its highly transient nature (Fig. 2E, H). Using a PIP₂ mass ELISA, we confirmed that PMA markedly increased PIP₂ concentrations, whereas PLD and PLD1 inhibitors reduced this response (Fig. 2I). Of note, PKCglo, but not the PKCα/β-selective inhibitor Gö6976 (PKCab), Akt-1/2 inhibitor (Akti), or DPI, reduced PIP₂ production, comparable to PLDi and PLD1i. This indicates that PIP₂ induction was reduced by inhibition of PLD1 and Ca^2+^-independent PKC isoforms, but was not significantly affected by Akt, NOX2 inhibition, or oxidative PPP inhibition (G6PDi, a glucose-6-phosphate dehydrogenase inhibitor^2^) after PMA stimulation. In contrast, when the neutrophils were stimulated with A23187, PLD and PLD1 inhibitors reduced A23187-induced PIP₂, whereas PKCglo, PKCab, Akti, DPI and G6PDi had no significant effect (Supplementary Fig. 4C); imaging of the corresponding conditions showed the same pattern (Supplementary Fig. 4D). These data suggest that the PKC contribution to PIP₂ generation is PMA-specific, whereas PLD1 is required for both stimuli.

To test whether the PLD1–PIP₂ axis operates under other stimuli, we examined three damage- and pathogen-associated molecular patterns (DAMPs and PAMPs): bacterial lipopolysaccharide (LPS), the chemotactic peptide N-formyl-methionyl-leucyl-phenylalanine (fMLP), and heme applied after a 30-min tumour necrosis factor-α (TNFα) priming step. These stimuli increased PIP₂ intensity, although to a lesser extent than PMA, as measured by quantitative immunofluorescence (Supplementary Fig. 4E–F).

Given that PA enhances the activity of phosphoinositide-synthesising enzymes, including PIP5K1 isoforms which convert PI4P to PIP₂^16,17^, we focused on PIP5K1 regulation. PMA increased neutrophil PIP5K1 activity, which was significantly reduced by PLD and PLD1 inhibition, mirroring PKC inhibition (Supplementary Fig. 5A). ROS production (DPI), oxidative PPP (G6PDi), Akt (Akti), or calcium-dependent PKC isoforms (PKCab) did not significantly affect PIP5K1 inducibility upon neutrophil PMA stimulation. The PIP5K1 isoforms PIP5K1A and PIP5K1C have immunological functions^21,22^. Therefore, we explored whether these PIP5K1 isoforms can bind to PA.

Recombinant PIP5K1A bound strongly to anionic phospholipids like phosphoinositides, phosphatidylserine, sulphogalactosylceramide, and cardiolipin, in a dot blot assay. PIP5K1C, however, selectively bound phosphoinositides (Supplementary Fig. 5B–C). PA, especially PA (18:0/18:1), PA (18:0/18:2), PA (18:1/18:1) as well as a PA mixture reflecting PMA-induced subspecies, enhanced PIP5K1A activity (Supplementary Fig. 5D). In contrast, PA subspecies reduced PIP5K1C activity, whereas phosphatidylcholine species increased it (Supplementary Fig. 5E). PMA triggered the relocalisation of both PIP5K1A and PIP5K1C to the nucleoplasm and cytoplasm where PIP₂ accumulates (Supplementary Fig. 5F). Although PLDi and PLD1i did not affect the localisation of these enzymes, they decreased PIP₂ synthesis (Supplementary Fig. 5F). Mechanistically, PA acts as an allosteric activator of PIP5K1A^23,24^; accordingly, PLD1 inhibition reduced PIP5K1A activity in neutrophils and the stimulus-induced rise in PIP₂ in parallel, even though PI subspecies remained detectable.

### PLD1 regulates reactive oxygen species production

**Fig. 3:**
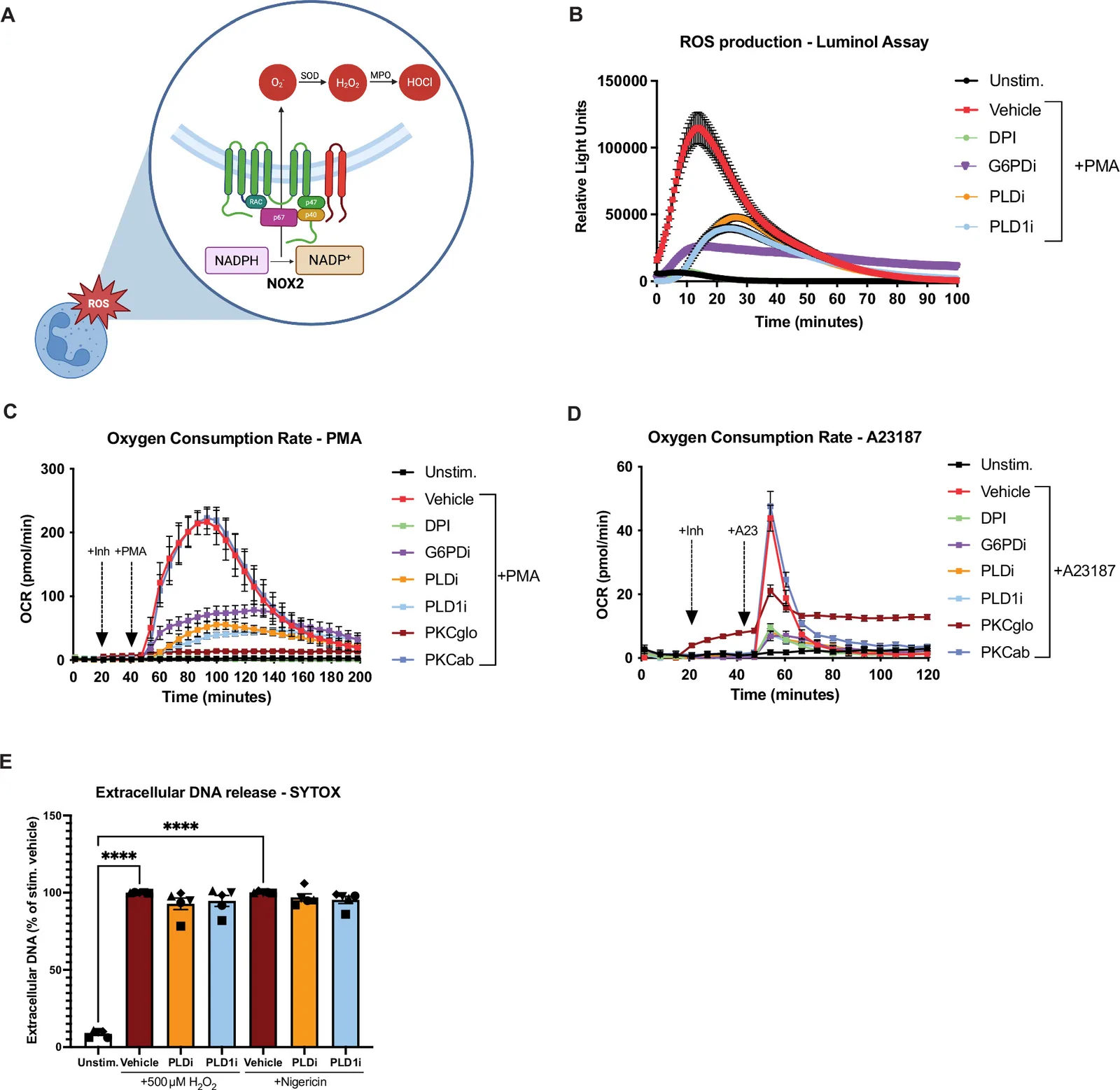
PLD1 is required for ROS production and NET formation. **A** Schematic of ROS generation by NOX2 in neutrophil. Created in BioRender. Patel, D. (2026) https://BioRender.com/1x6n143. **B** ROS production in response to PMA in the presence of the indicated inhibitors in neutrophils. **C** Oxygen consumption rate (OCR) of neutrophils pre-incubated with the indicated inhibitors and then stimulated with PMA. **D** OCR of neutrophils pre-incubated with the indicated inhibitor and then stimulated with A23187. **E** Extracellular DNA release, used here as a proxy for NETs, measured fluorometrically with cell-impermeable dye, SYTOX green. Neutrophils were pre-incubated with the indicated inhibitors before addition of either Nigericin or H₂O₂ for 4 h. **B**–**D** Data shown as mean ± s.e.m., *N* = 5 donors, 2 technical replicates per donor. **E** Data shown as mean ± s.e.m., *N* = 5 donors. Each point is one donor, plotted as the mean of 2 technical replicates and shown with a different symbol. **E** Statistical significance was determined by one-way ANOVA with Bonferroni’s multiple comparisons test. ****P < 0.0001. Exact *P* values are provided in the Source Data file. Source data are provided as a Source Data file.

ROS production is pivotal to mitogen- and microbial-induced NET formation mediated by the NOX2 enzyme (Fig. 3A). This NOX2-driven ROS production is fuelled by NADPH regenerated through the oxidative PPP (Supplementary Fig. 6A)^1,2^. PLD and PLD1 inhibition reduced and delayed PMA-, A23187-, and *C. albicans*-induced ROS in time course luminol assays (Fig. 3B and Supplementary Fig. 6B, C). As controls, both DPI^25^ and G6PDi^2^ effectively blocked ROS production (Fig. 3B). We confirmed these results showing that PLDi and PLD1i abrogated ROS upon PMA (Fig. 3C) or A23187 (Fig. 3D) stimulation when monitoring the oxygen consumption rate (OCR).

Furthermore, PLDi or PLD1i did not block H₂O₂-mediated DNA release (Fig. 3E), used here as a proxy for NET formation, suggesting that PLD1 functions upstream of NOX2 and myeloperoxidase (MPO) activity. Nigericin, an ionophore produced by *Streptomyces hygroscopicus*, activates a ROS-independent NETosis^5^ and, consistent with our model, PLD inhibitors did not affect NET formation with this stimulus (Fig. 3E). Taken together, these data indicate that PLD1 is required for ROS production during NETosis.

During phagocytosis, NOX2 is activated, prompting us to ask whether PLD1 is required for ROS production in this context as well. We incubated neutrophils with opsonised *Escherichia coli* at a multiplicity of infection (MOI) of 10, a dose routinely used to assess phagocytosis. PLDi and PLD1i reduced ROS during *E. coli* phagocytosis (Supplementary Fig. 6D) without affecting ingestion (Supplementary Fig. 6E). PLD activity triggered by the phagocytic stimuli *E. coli* and zymosan was blocked by the actin inhibitor Cytochalasin D (Supplementary Fig. 6F) and PLD inhibitors. These data indicate that PLD1 is required to activate NOX2 in different neutrophil effector functions.

### PLD1 deletion affects ROS and PIP₂ production in neutrophils derived from haematopoietic stem cells

To validate our pharmacological results, we exploited genetic depletion strategies. Neutrophils are terminally differentiated cells with a limited lifespan in which genetic manipulation is not possible. We therefore differentiated CD34^+^ stem cells isolated from human blood into neutrophil-like cells and knocked out PLD1 with CRISPR/Cas9^26^. PLD1-deficient cells displayed less PIP₂ accumulation post PMA stimulation than cultures transfected with a control sgRNA (Supplementary Fig. 6G) as observed by IF. Furthermore, upon PMA stimulation, PLD1-deficient cells also produced less ROS compared to cells transfected with a control sgRNA (Supplementary Fig. 6H).

### PLD1 regulates NAD kinase activity to promote ROS

**Fig. 4:**
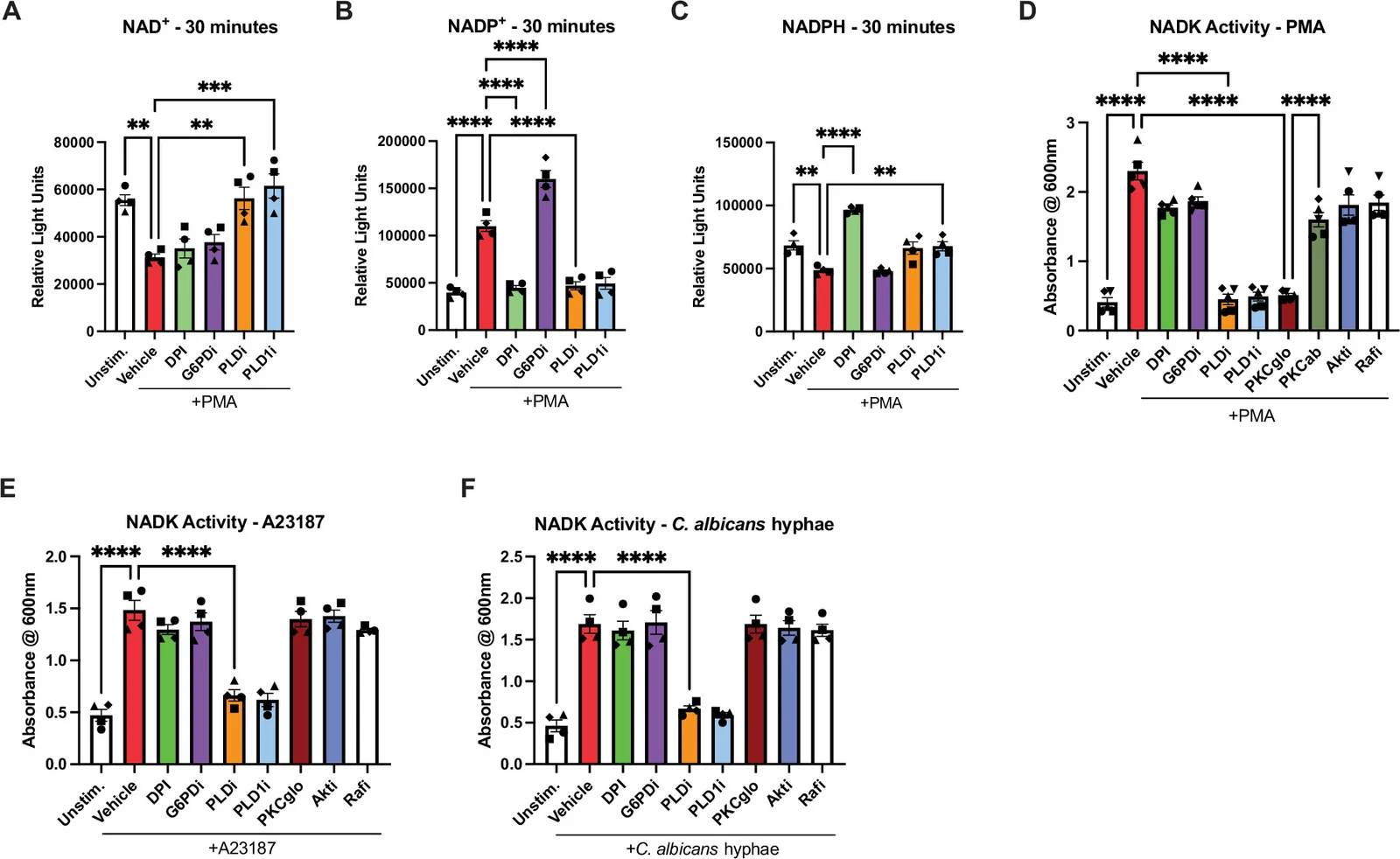
PLD1 promotes NADK activity to maintain NADP^+^ availability for ROS production. NAD^+^ (**A**), NADP^+^ (**B**) and NADPH (**C**) concentration in neutrophil lysates, incubated with the indicated inhibitors and stimulated with PMA for 30 min. The concentrations were determined by a luminescence assay. **D** NADK activity in neutrophil lysates of cells pre-incubated with the indicated inhibitors and stimulated for 30 min with PMA. **E** NADK activity in neutrophil lysates of cells pre-incubated with the indicated inhibitors and stimulated for 30 min with A23187. **F** NADK activity in neutrophil lysates of cells pre-incubated with the indicated inhibitors and stimulated for 30 min with *C. albicans* hyphae. **A**–**C**, **E**–**F** Data shown as mean ± s.e.m., *N* = 4 donors. Each point is one donor, plotted as the mean of 2 technical replicates and shown with a different symbol. **D** Data shown as mean ± s.e.m., *N* = 5 donors. Each point is one donor, plotted as the mean of 2 technical replicates and shown with a different symbol. **A**–**F** Statistical significance was determined by one-way ANOVA with Bonferroni’s multiple comparisons test. **P < 0.01, ***P < 0.001 and ****P < 0.0001. Exact *P* values are provided in the Source Data file. Source data are provided as a Source Data file.

NOX2 oxidises NADPH to NADP^+^ to generate ROS^1,2^ (Supplementary Fig. 6A). NADPH, in turn, is generated in the PPP by reducing NADP^+^ to NADPH (Supplementary Fig. 6A). To determine how PLD1 regulates this nicotinamide metabolic cycle, we quantified NAD^+^, NADP^+^ and NADPH at 10, 30 (this timepoint is highlighted in Fig. 4A–C) and 60 min after PMA stimulation in the presence or absence of NOX2, PPP or PLD inhibitors (Supplementary Fig. 7A–C).

In unstimulated neutrophils, NAD^+^, NADP^+^, and NADPH concentrations remained stable, but upon PMA stimulation, NAD^+^ rapidly decreased while NADP^+^ increased within 10 min (Supplementary Fig. 7A–C). NADPH levels remained relatively stable, consistent with continued reduction of NADP^+^ to NADPH via the oxidative PPP and its recycling back to NADP^+^ by NOX2. Supplementary Fig. 7D schematises this cycle, in which NAD kinase phosphorylates NAD^+^ to NADP^+^^27,28^.

NADP^+^ rose after stimulation and remained elevated throughout the time course, while it stayed unchanged in unstimulated cells, indicating that NADP^+^ production is dynamically tuned during activation. To interpret the NAD^+^, NADP^+^, and NADPH measurements, we first established how DPI and G6PDi affect them in PMA-stimulated neutrophils. NOX2 consumes NADPH to generate ROS and NADP^+^; blocking this step with DPI caused NADPH to accumulate and NADP^+^ to fall. The oxidative PPP regenerates NADPH from NADP^+^; blocking this step with G6PDi caused NADP^+^ to accumulate (Fig. 4A–C and Supplementary Fig. 7A–C), consistent with prior work linking oxidative PPP flux to NADPH supply for the NOX2 burst^2,29^.

PLD and PLD1 inhibition matched neither the DPI nor the G6PDi profile. Instead, the PMA-induced NADP^+^ rise was blunted, and NAD^+^ remained at unstimulated levels (Fig. 4A–C and Supplementary Fig. 7A–C). PMA activation lowers NAD^+^, and neither DPI nor G6PDi blocked this. This is the expected result when NADK, which phosphorylates NAD^+^ to NADP^+^, is impaired. This implies that PLD1 acts on NADK itself, upstream of both NOX2 and the PPP.

Consistent with a block in NADP^+^ synthesis, PLDi and PLD1i, but not DPI or G6PDi, reduced NADK activity after PMA stimulation (Fig. 4D). PLD inhibition also reduced NADK activity following A23187 stimulation or *C. albicans* hyphae exposure (Fig. 4E, F). Notably, PLD inhibitors did not affect the activity of recombinant NADK, excluding the possibility that PLDi or PLD1i had an off-target effect on this enzyme (Supplementary Fig. 7E).

We next examined how PLD1 inhibition affects extracellular acidification rate (ECAR). As expected, G6PDi reduced the ECAR response to PMA or A23187, as did PLD and PLD1 inhibition (Supplementary Fig. 6I–J). We interpret this as a reduction in activation-associated extracellular acidification, compatible with impaired PPP cycling or a broader reduction in activation-associated glucose flux; ECAR alone does not establish direct inhibition of glycolysis. Critically, the NAD^+^/NADP^+^/NADPH patterns under PLD inhibition differed markedly from those induced by G6PDi or DPI (Fig. 4A–C and Supplementary Fig. 7A–C), placing PLD1 upstream of NADK-mediated NADP^+^ synthesis rather than within the PPP or at NOX2. By reducing NADP(H) supply to NOX2, PLD1 inhibition therefore accounts for both the fall in ECAR and the block in NET release.

Phosphorylation of NADK by kinases such as Akt, Raf, or PKC enhances its activity^27,28,30^. Neither Akt (Akti) nor Raf (GW5074, Rafi) inhibitors influenced NADK activity in lysates from PMA-stimulated neutrophils (Fig. 4D). However, PKCglo, but not PKCab, decreased NADK activity (Fig. 4D), suggesting that a Ca^2+^-independent PKC isoform regulates NADK activity during PMA stimulation. PKCglo also reduced PMA-induced PLD activity to a similar extent as the PLD inhibitors themselves (Supplementary Fig. 3A), so its effect on NADK may be indirect. Interestingly, neither PKCglo, Akti nor Rafi affected NADK activity after A23187 or *C. albicans* hyphae stimulation, and only PLD and PLD1 inhibition did so (Fig. 4D–F). This implies that PLD-derived lipids can activate NADK independently of its phosphorylation.

### PLD1-derived phospholipids bind and regulate NADK activity

**Fig. 5:**
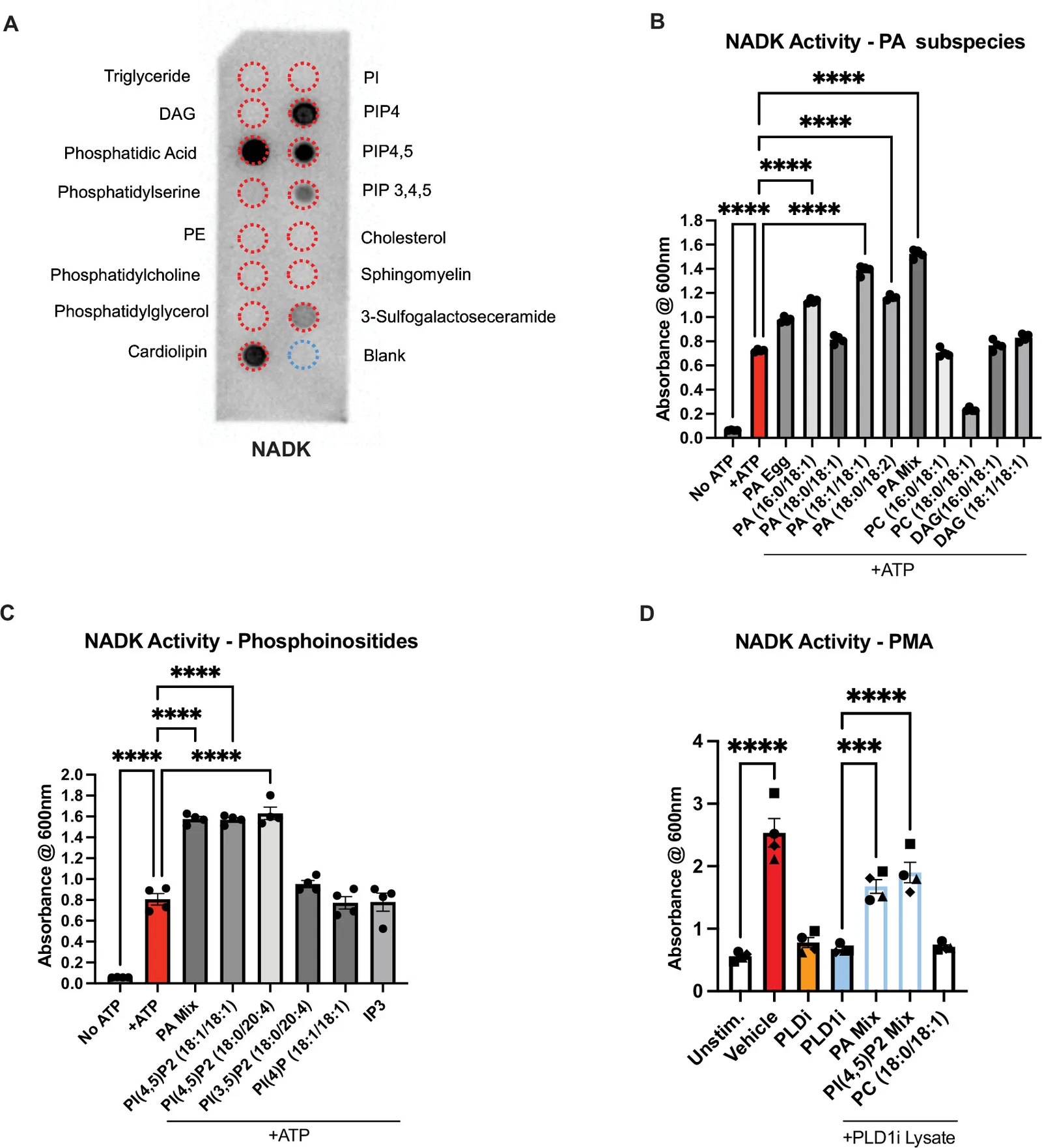
PLD1-derived phospholipids bind and activate NADK. **A** Representative dot blot of the indicated lipids with recombinant NADK. **B** Recombinant NADK was pre-incubated with the indicated PA, DAG, or PC lipids before measuring NADK activity in the presence of NAD^+^ and ATP. **C** Recombinant NADK was pre-incubated with the indicated phosphoinositide lipids before measuring NADK activity in the presence of NAD^+^ and ATP. **D** NADK activity in neutrophil lysates of cells pre-incubated with the indicated inhibitors and stimulated for 30 min with PMA. Lysate from PLD1i-PMA-treated neutrophils was incubated with a PA mix, PIP₂ mix, and PC (18:0/18:1), and NADK activity was assessed. A Representative blot from two independent experiments. **B**–**C** Data shown as mean ± s.e.m., *n* = 4 experiments. Each point is one experiment, plotted as the mean of 2 technical replicates. **D** Data shown as mean ± s.e.m., *N* = 4 donors. Each point is one donor, plotted as the mean of 2 technical replicates and shown with a different symbol. B–D Statistical significance was determined by one-way ANOVA with Bonferroni’s multiple comparisons test. ***P < 0.001 and ****P < 0.0001. Exact *P* values are provided in the Source Data file. Source data are provided as a Source Data file.

Since PLD1 promotes NADK activity in neutrophils, we examined whether PLD1-derived lipids directly regulate this enzyme. Recombinant NADK bound to anionic lipids, including PA, phosphoinositides, and cardiolipin, on lipid dot blots, but showed minimal interaction with zwitterionic lipids (Fig. 5A).

Pre-incubating recombinant NADK with PA 16:0/18:1, PA 18:1/18:1, PA 18:0/18:2 as well as a PA egg extract and a PA mixture of the subspecies identified in Fig. 1, enhanced NADK activity (Fig. 5B). Conversely, other phospholipids, such as DAG, failed to stimulate NADK activity, while specific PC species (e.g., PC 18:0/18:1) appeared to suppress it below baseline (Fig. 5B). PIP₂ species such as PI(4,5)P₂ (18:1/18:1) and PI(4,5)P₂ (18:0/20:4) also increased NADK activity, more so than other phosphoinositides (PI4P, PI(3,5)P₂) or IP3 (Fig. 5C). These data indicate that specific PA and PIP₂ species directly stimulate NADK activity.

Since NADK is a cytoplasmic protein, we extracted lysates from neutrophils to determine if lipid supplementation could rescue PLD1-mediated NADK activity inhibition. Supplementing lysates from PLD1-inhibited, PMA-stimulated cells with PA or PIP₂ restored NADK activity, whereas PC did not (Fig. 5D).

Because PIP₂ synthesis is downstream of PLD1 activation, we tested whether blocking PIP₂ production phenocopied PLD1 inhibition. Indeed, phosphatidylinositol 4-kinase (PI4K) inhibitors (PAO and PI4KAi, GSK-A1), which prevent generation of the PI4P precursor required for PIP₂ synthesis, inhibited PMA-induced NET formation as assessed by extracellular DNA release and IF after 4 h (Supplementary Fig. 8A–B). In contrast, inhibition of phospholipase C (PLC) with U-73122 (PLCi), which hydrolyses PIP₂ rather than contributing to its synthesis, did not impair NET formation (Supplementary Fig. 8A). PI4K inhibition also reduced NADK activity in neutrophils stimulated with PMA or *C. albicans* hyphae, whereas PLC inhibition had no effect (Supplementary Fig. 8C–D). Furthermore, consistent with earlier observations, PKCglo reduced NADK activity in PMA- (Supplementary Fig. 8C) but not hyphae-stimulated cells (Supplementary Fig. 8D), while PI4K inhibitors suppressed NADK activity under both conditions (Supplementary Fig. 8C–D). Together, these results indicate that PLD1-derived PA promotes NADK activity both directly and through downstream generation of PIP₂, and that disruption of PIP₂ synthesis is sufficient to block NADK activity and NETosis.

## Discussion

Neutrophil activation requires rapid coordination between membrane lipid remodelling, metabolic reprogramming, and effector functions such as ROS generation and NET release. Here we identify PLD1 as a key upstream regulator of all these processes. Using lipidomics, biochemical, microscopical and functional assays, we show that upon stimulation, PLD1 is essential for the production of PA, downstream phosphoinositides, NADP^+^ availability, and ultimately NETosis. These findings define a lipid–metabolic axis in which PLD1-dependent lipids directly regulate NADK, the sole enzyme generating NADP^+^, thereby coupling membrane dynamics to metabolic activation.

Lipidomic profiling revealed that neutrophil stimulation triggers rapid changes in phospholipids, especially in PA. Using a pharmacological approach, we demonstrated that PLD1, rather than PLD2, DAGK or NAPE-PLD, and, indirectly, the GPAT route through FASN inhibition, is the dominant source of PA during NETosis. PLD1 inhibition blunted the induction of major PA subspecies as well as the synthesis of PI and phosphoinositides.

PIP5K1 activity as well as phosphoinositide synthesis was reduced during PLD inhibition. PA promotes the activity of PIP5K1 isoforms^16,17^. We found that the PIP5K1A isoform selectively bound anionic lipids, particularly PA and phosphoinositides, and that PA enhanced PIP5K1A activity. Because PIP₂ regulates cytoskeletal organisation, vesicle formation and trafficking as well as protein function^19,31,32^, reduced PIP₂ synthesis likely contributes to the broad defects in NET formation observed upon PLD1 inhibition. Together, these data position PLD1 upstream of a lipid cascade that coordinates the phosphoinositide landscape required for efficient neutrophil activation.

The relative contributions of de novo PI synthesis (via the CDP-DAG/PI cycle)^20^ versus turnover of pre-existing pools remain an open question that the present data do not resolve. Our data instead support PLD1-derived PA acting as an allosteric input on PIP5K1A rather than as a determinant of bulk PI subspecies abundance.

A central finding of this report is that PLD1 regulates neutrophil NADP^+^/NAD^+^ metabolism through direct lipid-dependent activation of NADK. PLD1 inhibition consistently reduced NADP^+^ and increased NAD^+^ during PMA stimulation, indicating impaired NAD^+^ phosphorylation and placing NADK downstream of PLD1. Notably, this NADP^+^/NAD^+^ shift reflected impaired NADK activity rather than altered oxidative-PPP flux or NOX2 activity. Although PA was reported to interact with subunits of the NOX2 complex^33^, PLD1 inhibition did not reproduce the metabolic or ROS phenotypes observed with direct NOX2 inhibition. Instead, we found that PLD1 regulates cellular NADK activity across multiple stimuli, establishing NADK as the downstream metabolic effector linking PLD1-derived lipids to NADP^+^ production.

Activated neutrophils engage the PPP to power the NADPH-dependent oxidative burst^2^, and pharmacological diversion of glycolytic flux away from the PPP depletes cellular NADPH and suppresses the NOX2-dependent burst^29^. Our direct nicotinamide-nucleotide measurements place PLD1, via NADK, upstream of this PPP-dependent step: by setting the size of the NADP^+^ pool, PLD1 governs the NADP(H) supply on which PPP-coupled NADPH regeneration and, ultimately, NOX2-dependent NET release depend.

Reconstitution assays showed that NADK binds acidic phospholipids and that defined PA and PIP₂ species enhance its enzymatic activity. Although phosphorylation by kinases such as PKC^28^ and Akt^27^ modulates NADK function, our findings identify membrane phospholipids as direct activators of NADK, revealing an additional regulatory mechanism. Recent work demonstrated that NADK activity can also be enhanced by palmitoylation mediated by the palmitoyl-transferase ZDHHC5^34^. While PA can contribute to cellular fatty-acid pools, including palmitate (16:0) used for protein acylation, we demonstrate that PA and phosphoinositide species lacking 16:0 directly stimulate NADK activity, positioning NADK as a lipid-responsive metabolic node.

Direct dissection of lipid binding to NADK, for example via point mutations in candidate PA/PIP₂-binding residues, will require structural identification of those residues and a heterologous expression system, and is a priority for future work.

Previous studies have implicated PLD in NETosis. A recent in vivo study using PLD1-deficient mice showed impaired ROS production and NET release, underscoring the physiological relevance of PLD1 during inflammation^35^. A separate lipidomics-focused study reported PA generation upon PMA stimulation in human neutrophils and suggested that PLD contributed to NET formation^36^. However, these reports did not resolve how PLD-derived lipids support the metabolic requirements of NETosis. Our work extends these observations by identifying NADK as the lipid-regulated metabolic effector that sustains NADP^+^ supply for PPP-coupled NADPH regeneration, ROS generation, and NET release.

Consistent with a requirement for ROS production rather than for post-ROS NET-execution events, PLD1 inhibition did not impair NET formation driven by exogenous H₂O₂ (which bypasses NOX2) or by nigericin (a ROS-independent stimulus). Whether the PLD1–NADK axis additionally contributes to mitochondrial-ROS-driven vital NETosis was not tested here and remains an open question.

NADK is implicated in a range of pathological settings, including oxidative stress^37^ and metabolic support of tumour growth^28,38^, underscoring the broader importance of understanding how its activity is regulated. Although previous work has focused largely on kinase-dependent phosphorylation as the principal mechanism controlling NADK activity^27,28^, our findings reveal a lipid-directed mode of regulation in which PA and PIP₂ directly enhance NADK activation. This places NADK at the intersection of lipid- and kinase-derived inputs, allowing dynamic tuning of NADP^+^ production during neutrophil activation.

PLD activity has been shown in other cell types to generate PA at defined membrane regions^11,39^, providing spatially restricted lipid signalling. Lipid-directed regulation of NADK therefore provides a mechanism to position enzyme activity at these sites. Local PA and PIP₂ production could help position NADK activity at specific sites of neutrophil activation. Because NADP^+^ is membrane-impermeable, such spatial lipid regulation may also contribute to localised NADP^+^ generation, ensuring that NADP^+^ availability aligns with metabolic demand during cell activation.

Together, these results suggest that lipid–NADK coupling may represent a broader regulatory principle that could influence additional neutrophil effector functions beyond NETosis, as well as NADP^+^-dependent pathways operating in other immune and disease contexts.

Future in vivo studies will be required to define how PLD1–NADK coupling operates during infection and inflammation. Lipid supplementation did not restore NADK activity in intact cells, likely reflecting constraints in lipid delivery or subcellular localisation. Given the central role of NADK and NADP^+^ in cell survival, metabolism and proliferation^37^, lipid-mediated regulation of NADK may therefore have broader metabolic consequences beyond neutrophil ROS production.

Our study has limitations. We have not measured glycolytic or PPP flux directly; our metabolic conclusions rest on the NADP(H) pool measurements and on ECAR interpreted as activation-associated acidification. More generally, inhibitor-based conclusions have intrinsic limitations; where possible, pharmacological perturbations were corroborated by PLD1-CRISPR genetic loss-of-function, and instances in which pharmacological and genetic results diverge are noted as such. Although we observed that DAMPs and PAMPs induced PIP₂ production, further studies will be required to confirm that this occurs via PA and PLD1 activity.

Together, our findings define PLD1 as a central organiser of lipid and metabolic remodelling during neutrophil activation. Through the coordinated production of PA and PIP₂, PLD1 promotes NADK activity, maintains NADP^+^ availability and supports the metabolic programme required for ROS generation and NET release. This work identifies lipid-mediated NADK activation as a regulatory mechanism linking membrane lipid dynamics to metabolic control in innate immunity.

## Methods

### Human subjects and neutrophil isolation

Experiments using human samples were conducted in accordance with the Declaration of Helsinki. Blood samples and buffy coats were collected from anonymous healthy adult donors with consent and approval from the Charité Hospital ethics committee in Berlin, Germany (EA1/0104/06). Donor age was not recorded, and donors received no compensation. Donor sex and gender were not recorded at collection, either by self-report or by assignment, and cannot be linked to individual samples. Sex- and gender-disaggregated analyses were therefore not possible, and none were performed. Our findings do not apply to only one sex or gender, and the cellular and biochemical mechanisms we describe are not expected to be sex- or gender-specific. Neutrophils were isolated using two different methods. The first isolation method is a two-step density separation used for extracellular DNA release, phagocytosis/*E. coli* ingestion, luminol assays, and all microscopy experiments. Peripheral blood was layered over an equal volume of Histopaque-1119 and centrifuged at 800 × *g* for 20 min. PBMC and neutrophil layers were collected separately, washed with PBS containing 0.2% human serum albumin (HSA), and pelleted at 300 × *g* for 10 min. The neutrophil pellet was resuspended in PBS + 0.2% HSA and layered onto a discontinuous Percoll gradient (85%–65%, 2 mL layers) and centrifuged at 800 × *g* for 20 min. The neutrophil band was collected, washed, pelleted (300 × *g*, 10 min), and counted using a CASY Counter (OMNI Life Science). The second isolation method was achieved utilising an EasySep™ Direct Human Neutrophil Isolation Kit (Stemcell). The isolation was performed as per manufacturer’s instructions, and the buffer used for the isolation was 0.05% HSA, 1 mM EDTA in PBS. This isolation method was used for the lipidomics screen, nucleotide metabolism, Seahorse, PLD activity, PIP5K1 activity, NADK activity, and free choline assays.

### Inhibitors

Unless stated otherwise, neutrophils were pretreated with inhibitors for 30 min at 37 °C prior to stimulation, and vehicle controls received an equivalent volume of DMSO (0.001% final). The following inhibitors were used at the indicated final concentrations: ML299 (PLDi, pan-PLD; Sigma Aldrich, 1 µM), FIPI (pan-PLD; Sigma Aldrich, 1 µM), VU0359595 (PLD1i, PLD1; Sigma Aldrich, 1 µM), VU0155069 (PLD1; Sigma Aldrich, 1 µM), VU0364739 (PLD2; Tocris, 1 µM), VU0155056 (PLD2; Sigma Aldrich, 1 µM), diphenyleneiodonium (DPI, NOX2; Sigma Aldrich, 10 µM), BAY 85-8501 (NEi, neutrophil elastase; MedChemExpress, 5 µM), Gö6983 (PKCglo, pan-PKC; Sigma Aldrich, 1 µM), Gö6976 (PKCab, PKCα/β; Sigma Aldrich, 1 µM), GW5074 (Rafi, Raf; Sigma Aldrich, 10 µM), Akt-1/2 inhibitor (Akti; Sigma Aldrich, 5 µM), glucose-6-phosphate dehydrogenase inhibitor (G6PDi; Sigma Aldrich, 50 µM), phenylarsine oxide (PAO, PI4K; Sigma Aldrich, 1 µM), GSK-A1 (PI4KAi, PI4KA; Sigma Aldrich, 1 µM), U-73122 (PLCi, PLC; Sigma Aldrich, 5 µM), Cytochalasin D (CytoD, actin polymerisation; Sigma Aldrich, 5 µM), LEI-401 (NAPEi, NAPE-PLD; Tocris, 10 µM), C-75 (FASNi, fatty acid synthase; Sigma Aldrich, 50 µM) and diacylglycerol kinase inhibitor 1 (R59022, DAGKi; Sigma Aldrich, 10 µM).

### Lipidomics sample preparation

For lipidomics, 1 × 10⁷ neutrophils were plated on Petri dishes (Greiner Bio-One) and pretreated with vehicle (0.001% DMSO) or 1 µM VU0359595 (PLD1i) for 30 min before stimulation with 50 nM PMA for 30, 60, or 180 min. Cells were treated with DNase I (10 U/mL, ThermoFisher) for 10 min, washed with ice-cold PBS, scraped into 500 µL PBS, briefly sonicated, and stored at −80 °C until analysis.

### Lipidomics – shotgun mass spectrometry

Mass spectrometry-based lipid analysis was performed on cell lysates by Lipotype GmbH (Dresden, Germany)^40^. Two lipidomic datasets were acquired. The PMA time course comprised four conditions (unstimulated, and PMA for 30, 60 and 180 min) from *N* = 3 donors, each analysed in three technical replicates (36 samples). The PLD1 inhibitor experiment comprised six conditions (unstimulated and PMA for 30 or 180 min, each with vehicle or 1 µM VU0359595) from *N* = 3 donors, each analysed in two technical replicates (36 samples). Blank samples and quality-control reference samples were processed in the same analytical runs. Lipids were extracted using a two-step chloroform/methanol procedure^41^. Samples were spiked with internal lipid standard mixture containing: cardiolipin 14:0/14:0/14:0/14:0 (CL), ceramide 18:1;2/17:0 (Cer), diacylglycerol 17:0/17:0 (DAG), hexosylceramide 18:1;2/12:0 (HexCer), lyso-phosphatidate 17:0 (LPA), lyso-phosphatidylcholine 12:0 (LPC), lyso-phosphatidylethanolamine 17:1 (LPE), lyso-phosphatidylglycerol 17:1 (LPG), lyso-phosphatidylinositol 17:1 (LPI), lyso-phosphatidylserine 17:1 (LPS), phosphatidate 17:0/17:0 (PA), phosphatidylcholine 17:0/17:0 (PC), phosphatidylethanolamine 17:0/17:0 (PE), phosphatidylglycerol 17:0/17:0 (PG), phosphatidylinositol 16:0/16:0 (PI), phosphatidylserine 17:0/17:0 (PS), cholesterol ester 20:0 (CE), sphingomyelin 18:1;2/12:0;0 (SM), triacylglycerol 17:0/17:0/17:0 (TAG). After extraction, the organic phase was transferred to an infusion plate and dried in a speed vacuum concentrator. The dry extract was resuspended in 7.5 mM ammonium formate in chloroform/methanol/propanol (1:2:4, V:V:V). All liquid handling steps were performed using the Hamilton Robotics STARlet robotic platform with the Anti Droplet Control feature for organic solvent pipetting. Samples were analysed by direct infusion, without chromatographic separation, on a Q Exactive hybrid quadrupole-Orbitrap mass spectrometer (Thermo Fisher Scientific) equipped with a TriVersa NanoMate automated nano-electrospray ion source (Advion Biosciences), in both positive and negative ion mode. Lipids were identified from unprocessed (.raw) spectra using LipotypeXplorer, an in-house identification software based on LipidXplorer^42,43^, and filtered on mass accuracy, occupation threshold, noise, and background. Glycerophospholipids, diacylglycerol, triacylglycerol and cholesteryl esters were identified from MS/MS spectra following collision-induced fragmentation, giving acyl-chain (subspecies) resolution; the remaining classes were identified from MS spectra at the species level. Only lipid identifications with a signal-to-noise ratio >5 and a signal intensity 5-fold higher than in the corresponding blank samples were considered for further analysis. Lipids were quantified by normalising the intensity of each species to that of its lipid class-specific internal standard and are reported in pmol.

### Extracellular DNA release (SYTOX Green NETosis assay)

Neutrophils (1 × 10^6^/mL) were incubated with SYTOX Green (1:50,000, ThermoFisher Scientific, 5 mM stock) for 10 min at 37 °C. 100 µL (1 × 10⁶ neutrophils) were plated per well in 96-well plates, pretreated with inhibitors for 30 min at 37 °C, and stimulated with vehicle, PMA (50 nM), A23187 (Sigma Aldrich, 5 µM), *Candida albicans* hyphae (MOI = 1), H₂O₂ (Sigma Aldrich, 500 µM), or nigericin (Sigma Aldrich, 15 µM). Fluorescence (Ex/Em 488/518 nm) was measured after 4 h on an Agilent BioTek Synergy H1 plate reader using Gen5 (version 3.14, Agilent BioTek). For each stimulus, extracellular DNA release was expressed as a percentage of its own stimulated vehicle control, which was set to 100%.

### Luminol ROS assay

Neutrophils (1 × 10⁶ cells/well) were pretreated with inhibitors for 30 min at 37 °C and stimulated with vehicle, PMA (50 nM), A23187 (5 µM), *E. coli* K-12 (MOI = 10), or *C. albicans* hyphae (MOI = 1). HRP (1.2 U/mL final) and luminol (50 µM) were added, and luminescence was monitored kinetically using a VICTOR Light luminescence counter (Perkin Elmer).

### PLD activity assay

PLD activity was quantified using a colorimetric assay kit (Sigma Aldrich). Neutrophils (5 × 10⁶) were pretreated with inhibitors for 30 min and stimulated with PMA, A23187, *C. albicans* hyphae, opsonised *E. coli*, and opsonised Zymosan A (*S. cerevisiae*, ThermoFisher Scientific, MOI = 10). Cells were pelleted (300 xg, 10 min), washed with cold PBS, resuspended in 50 µL assay buffer, and processed according to the manufacturer’s instructions.

### Free choline assay

Free choline levels were measured using a fluorescent kit (Abcam). Neutrophils (1 × 10^7^) were pretreated and stimulated as above, washed, lysed in 50 µL assay buffer, and processed according to manufacturer protocols.

### PIP₂ and PIP₃ ELISA

A PIP₂ and PIP₃ ELISA was performed on neutrophil lysates (Echelon) according to manufacturer’s protocols. To prepare the lysates, 1 × 10^7^ neutrophils were pretreated with vehicle or inhibitors for 30 min prior to stimulation with vehicle, 50 nM PMA, or 5 µM A23187. Neutrophils were then spun down at 300 × *g* for 10 min and washed once with ice-cold PBS. Neutrophils were then washed with 5% TCA/1 mM EDTA before acidic lipids were removed using MeOH:CHCl3 (2:1) (Sigma Aldrich) from the cell lysates. Neutral lipids from the remaining cell pellet were then extracted using MeOH:CHCl3:HCl (80:40:1) (Sigma Aldrich) and isolated by a phase split of the supernatant, collecting the organic phase layer. The remaining solvents were evaporated using a vacutainer and the remaining lipid film was frozen at −20 °C until the experiment. The samples were prepared and run on the ELISA as per the kit protocol (Echelon).

### PIP5K1 activity assay (cell lysates)

Neutrophils (1 × 10⁷) were pretreated with inhibitors for 30 min and stimulated with PMA or A23187. Cells were lysed in RIPA buffer containing HALT protease/phosphatase inhibitors (ThermoFisher Scientific). Lysates were cleared (15,000 × *g*, 15 min) and analysed using Echelon’s PIP5K1 activity kit.

### Lipid–protein interaction assays

Lipid strips (Echelon) were blocked in TBS-T + 3% BSA for 1 h. Recombinant NADK (Adipogen), PIP5K1A or PIP5K1C (Reaction Biology) (5 µg each) were incubated with membranes overnight at 4 °C. After washing, membranes were incubated overnight with anti-NADK (Cell Signaling Technology, #55948, rabbit polyclonal, lot 1, 1:1000), anti-PIP5K1A (ThermoFisher Scientific, MA5-47257, clone HL2011, lot AA46625051, 1:1000) or anti-PIP5K1C (ThermoFisher Scientific, 702444, clone 16H4L5, lot 2554946, 1:1000), followed by Peroxidase AffiniPure goat anti-rabbit IgG, F(ab′)₂ fragment specific (Jackson ImmunoResearch, 111-035-047, lot 000000135784, 1:1000) for 1 h. Membranes were washed and developed with SuperSignal™ West Pico PLUS (ThermoFisher Scientific) and imaged using a Gel Doc XR+ Gel Documentation System with Image Lab (version 5.2.1 build 11, Bio-Rad).

### Immunofluorescence staining of NETs (3D9)

Neutrophils (1 × 10⁵/well) were plated on Ibidi 8-well chambers, pretreated for 30 min with inhibitors, and stimulated with PMA for 4 h. Cells were fixed with 4% PFA (15 min), permeabilised with 0.1% Triton X-100, blocked in 1% BSA/0.5% saponin solution, and stained overnight with an in-house mouse monoclonal antibody against cleaved histone H3^13^ (clone 3D9, 1:200). Bound antibody was detected with goat anti-mouse IgG (H + L) highly cross-adsorbed secondary antibody, Alexa Fluor 488 (ThermoFisher Scientific, A-11029, lot 1789729, 1:1000), and cells were counterstained with DAPI (1 µg/mL). Images were acquired on a Leica SP8 AOBS confocal microscope using a 40× oil objective. Z-stacks (2048 × 2048) were processed using LAS X (version 3.5.2.18963, Leica Microsystems).

### Immunofluorescence staining of PLD1 and phosphoinositides

Neutrophils (1 × 10⁵/well) were pretreated, stimulated (PMA 30 or 180 min; A23187 10 min; DAMP/PAMPs: LPS (1 µg/mL), fMLP (100 nM), or heme (25 µM) applied after a 30-min TNFα (100 ng/mL) priming step), fixed with 4% PFA, permeabilised with 0.05% saponin and blocked in 1% BSA. Primary antibodies were anti-PLD1 (Abcam, ab235997, clone EP1506Y, lot 1056216-18, 1:400), anti-PtdIns(4)P IgM (Echelon Biosciences, Z-P004, clone PI4-2, lot XCM091624-21, 1:250), anti-PtdIns(4,5)P₂ IgM (ThermoFisher Scientific, MA3-500, clone 2C11, lot AB399605, 1:250), anti-PtdIns(3,4,5)P₃ IgM (Echelon Biosciences, Z-P345, clone RC6F8, lot AB102821-23, 1:250), anti-PIP5K1A (ThermoFisher Scientific, MA5-47257, clone HL2011, lot AA46625051, 1:200) and anti-PIP5K1C (ThermoFisher Scientific, 702444, clone 16H4L5, lot 2554946, 1:200). Secondary antibodies were F(ab′)₂-goat anti-rabbit IgG (H + L) cross-adsorbed Alexa Fluor 488 (ThermoFisher Scientific, A-11070, lot 2563831) and goat anti-mouse IgG (H + L) cross-adsorbed Alexa Fluor 647 (ThermoFisher Scientific, A-21235, lot 3192624), both at 1:1000. All fixed cells were stained with DAPI (Sigma Aldrich, 1:1000). Images were acquired on a Leica SP8 AOBS confocal microscope using a 100× or 63× oil objective. Z-stacks (1024×1024 or 2048×2048) were processed using LAS X.

### Quantification of nuclear area and 3D9 intensity

Image analysis was performed in Fiji (ImageJ version 1.54) on confocal images acquired with a 40× oil objective at 2048×2048 pixels. Neutrophils were stained for DNA (DAPI) and the NET-specific cleaved histone H3 antibody (3D9). NETs were quantified with a separate Fiji-based approach, not the single-cell watershed pipeline used for the phosphoinositide panels. A NET’s decondensed, diffuse chromatin lacks the compact nucleus that the watershed segmentation needs, so it is not reliably recognised. Because activated cultures contain a mixture of intact cells (compact, lobulated nuclei) and NETs (expanded, decondensed chromatin), all DAPI^+^ objects were segmented from the DAPI channel by automated thresholding and measured without pre-selection. For each object, the DAPI^+^ area was recorded using Analyze Particles as a readout of chromatin decondensation, and the 3D9 mean fluorescence intensity was measured within the same region as a readout of NET-associated histone H3 cleavage. A shift in the population towards larger DAPI^+^ areas and higher 3D9 signal reflects increased NET formation. Objects touching the image border were excluded, and ≥35 DAPI^+^ objects per donor per condition (two independent donors) were retained.

### Phosphoinositide and cytoplasmic PLD1 quantification

These mediators were detected with AF488- and AF647-conjugated secondary antibodies, and nuclei were stained with DAPI. Images were acquired on Leica confocal microscopes and processed with LAS X (version 3.5.2.18963, Leica Microsystems). PIP₂ panels were acquired with an HC PL APO 63×/1.32 oil-immersion objective at 2048×2048 pixels, whereas PI4P and PIP₃ panels were acquired at 1024×1024 pixels on an SP8 AOBS with a 100× oil objective. For each target, all conditions were imaged in the same run and analysed with a fully scripted pipeline in Python (version 3.12.5) with scikit-image (version 0.26.0)^44^. Each channel was recorded across all Z planes, and the stack was combined by sum-intensity projection before measurement. Background was removed in two stages: a per-slice rolling-ball estimate per channel, followed by a per-cell local correction subtracting the median intensity of a surrounding annular (donut-shaped) ring, excluding neighbouring cells and image borders, with an image-wide non-cell median as a fall-back. Cells were segmented by a marker-controlled watershed^45^; the DAPI-stained nucleus of each cell served as the starting marker (Gaussian smoothing, Li thresholding and morphological closing, which merges the lobes of a single polymorphonuclear nucleus without joining adjacent cells), and the cell footprint was defined by Li (or, as a fall-back, triangle) thresholding of the combined PLD1-and-phosphoinositide signal. For each cell the nucleus was excluded, and intensities were measured for PLD1 and the phosphoinositide channel; we report the mean fluorescence intensity. For PLD1, the rim (0.45 µm wide, a plasma-membrane proxy) was additionally excluded and the mean cytoplasmic fluorescence intensity measured in the interior compartment. Cells touching the image border, or with a cross-sectional area outside 20–2000 µm^2^, were excluded, and the number of cells retained per condition per donor is specified as a range in each figure legend (two independent donors). For each target, the pooled single-cell distributions were compared across conditions by one-way ANOVA with Bonferroni’s multiple comparisons test.

### Seahorse OCR and ECAR measurements

Neutrophils (5 × 10^4^/well) were plated onto Cell-Tak–coated Seahorse XF96 plates and allowed to adhere. Cells were pretreated for 30 min and stimulated with PMA or A23187. OCR and ECAR were measured in real time (3 baseline cycles + 30 kinetic cycles) on a Seahorse XF96 Analyzer, with data acquired and exported using Wave (version 2.4.3.7, Agilent).

### Phagocytosis assay

AF488-labelled *E. coli* (K-12, ThermoFisher) were opsonised with human serum IgG (Sigma Aldrich) for 30 min at 37 °C. The *E. coli* were then added to neutrophils at MOI = 10 and incubated for 30 min at 37 °C. After washing and quenching extracellular fluorescence with trypan blue, the samples were analysed on a CytoFlex flow cytometer (Beckman Coulter) using CytExpert (version 2.3, Beckman Coulter), and flow data were analysed in FlowJo (version 11.1.1, BD Biosciences). Neutrophils were gated on forward/side scatter, and phagocytosis was quantified as the percentage of AF488^+^ neutrophils. A representative flow cytometry gating strategy is displayed in Supplementary Fig. 9.

### Preparation of *C. albicans* hyphae

*C. albicans* (clinical isolate, SC5314) was cultured overnight in YPD at 30 °C, washed, and resuspended at 1 × 10⁷/mL in RPMI + 10% FCS for 4 h to induce hyphae. Hyphae were washed and heat-killed at 90 °C for 1 h. The hyphae were opsonised with human serum IgG (Sigma Aldrich) for 30 min at 37 °C prior to incubation with neutrophils.

### Measurement of NAD^±^, NADP^±^, and NADPH

Nucleotides were quantified using NAD^+^/NADH-Glo and NADP^+^/NADPH-Glo kits (Promega). Neutrophils (1 × 10⁶) were pretreated for 30 min and stimulated for 10, 30, or 60 min with PMA. Luminescence was measured on an Agilent BioTek Synergy H1 plate reader using Gen5 (version 3.14, Agilent BioTek).

### NADK activity assay (neutrophil lysates)

Neutrophils (1 × 10⁷) were lysed in Tris-based lysis buffer containing NaCl, EDTA, NaF, β-glycerophosphate, PNPP, PMSF, and HALT inhibitors. 10 µg of lysate was incubated in reaction buffer (50 mM Tris-HCl pH 8, 10 mM MgCl₂, 1 mM NAD^+^, 5 mM ATP; 100 µL total) at 37 °C for 20 min. Reactions were boiled for 3 min, cooled, and mixed with detection buffer (62.5 mM Tris-HCl pH 8, 6.25 mM MgCl₂, 6.25 mM glucose-6-phosphate, 1.875 mM PMS, 0.624 mM MTT). The reaction was initiated by adding 1 U glucose-6-phosphate dehydrogenase and measuring absorbance at 600 nm.

### Recombinant NADK biochemical assay

Recombinant human NADK (100 ng/reaction) was pre-incubated with lipid species (25 µM, Avanti) for 15 min at 25 °C. The PA mix group is composed of the most induced PA subspecies (i.e., PA 16:0/18:1, PA 18:0/18:1, PA18:1/18:1, and PA18:0/18:2) mixed in equal proportions in chloroform prior to evaporation. NADK activity was initiated by adding reaction buffer (50 mM Tris-HCl pH 8, 10 mM MgCl₂, 1 mM NAD^+^, 5 mM ATP; 100 µL) and incubating at 37 °C for 15 min. Reactions were stopped at 95 °C for 3 min and NADK activity quantified as above through PMS/MTT-coupled detection.

### CD34^±^ HSPC isolation and neutrophil-like differentiation

CD34^+^ haematopoietic stem and progenitor cells were isolated from human buffy coats by density-gradient centrifugation over Histopaque-1077 (400 × *g*, 35 min, no brake) to obtain PBMCs, followed by red-cell lysis in ammonium chloride buffer (10 min, 4 °C) and magnetic-activated cell sorting (MACS) using the CD34 MicroBead Ultra-Pure Kit (Miltenyi Biotec). CD34^+^ cells were seeded at 1 × 10^5^ cells/mL in IMDM, maintained at 5 × 10^5^ cells/mL, and differentiated over 28 days using a stage-specific cytokine regimen adapted from published protocols^26,46–48^. During the expansion phase (Days 0–14), cells were cultured on untreated polystyrene plates with 10% human serum, SR-1 (1 µM), UM729 (1 µM), SCF (50 ng/mL), FLT3L (50 ng/mL), IL-3 (10 ng/mL), TPO (20 ng/mL), and cholesterin (10 µg/mL). From Day 14, serum was reduced to 5%, SR-1, UM729, TPO, and cholesterin were removed, and cells were transferred to fibronectin-coated plates (StemCell Technologies). To drive maturation, IL-6 (10 ng/mL) and G-CSF (10 ng/mL) were introduced on Day 14 and maintained continuously; SCF and FLT3L were halved to 25 ng/mL on Days 17–18 and then withdrawn; IL-3 was maintained at 10 ng/mL until Days 17–18 before withdrawal; GM-CSF was introduced at 10 ng/mL on Day 14, decreased to 5 ng/mL on Days 17–18, then removed. To promote terminal neutrophilic maturation, DHT (100 nM) and nicotinamide (1 mM) were added from Day 24 onwards. Donor sex and gender were not recorded at collection, either by self-report or by assignment, and cannot be linked to individual samples. Sex- and gender-disaggregated analyses were therefore not possible, and none were performed. Our findings do not apply to only one sex or gender, and the cellular and biochemical mechanisms we describe are not expected to be sex- or gender-specific.

### CRISPR-Cas9 mutagenesis of PLD1

For PLD1 knockout, CD34^+^ HSPCs were expanded for 10 days prior to electroporation. Ribonucleoprotein (RNP) complexes were assembled by combining Cas9-GFP protein (IDT, Cat. No. 10008161; 5 µg per 10^6^ cells) with a sgRNA targeting PLD1 exon 14 (5′-GAGCACAGACTCACAGACGT-3′, chr3q26.31) or a control sgRNA targeting TLR4 (5′-AGAACCAAACGATGGACTTC-3′) at a 1:5 molar ratio (Cas9:sgRNA), incubated at room temperature for 10–15 min. Cells were resuspended in Opti-MEM at 2 × 10^6^ cells/mL, mixed with the RNP complex, and electroporated using a NEPA21 electroporator (Nepagene) with the following settings: Poring pulse, 175 V, 5 ms pulse length, 50 ms interval, 2 pulses, 10% decay, positive polarity; Transfer pulse, 20 V, 50 ms pulse length, 50 ms interval, 5 pulses, 40% decay, ± polarity. Twenty-four hours post-electroporation, GFP^+^ cells were isolated by FACS and returned to the stage-specific differentiation culture. Editing efficiency was assessed at Days 21–28 by extracting genomic DNA (Qiagen DNeasy Kit), PCR-amplifying the PLD1 target locus (forward 5′-AGTATATGGGCGTGGGAGCTTG-3′, reverse 5′-CTAGGTGGGGAAGCTGGTGTTT-3′; Q5 High-Fidelity Polymerase, NEB) and Sanger sequencing (Eurofins Genomics; sequencing primer 5′-GCTTTTCAGGGAGAACACAGACC-3′); indel frequency was quantified using TIDE^49^. Editing at the control locus was assessed in parallel by amplifying the TLR4 locus (forward 5′-TCCAACAAAGGTGGGAATGCTT-3′, reverse 5′-CTGCCTCTGGTCCTTGATCCAT-3′) and Sanger sequencing (sequencing primer 5′-TGAAAAGAAGTTGTTGTGGCTCA-3′); no editing was detected in control cells.

### Statistical analysis

Statistical analyses were performed in GraphPad Prism (version 11.0) and in R (version 4.3.3) with limma (version 3.58.1)^50^. For each donor, or for each independent experiment in cell-free assays, technical replicates were averaged to give a single value per condition. For the shotgun lipidomics datasets, technical replicates were averaged per donor and donor-mean concentrations were log₂-transformed; differential abundance was assessed by fitting linear models across all conditions of each dataset with donor as a blocking factor and obtaining moderated t-statistics by empirical Bayes variance moderation, and *P* values were adjusted across lipid species by the Benjamini–Hochberg method^51^. For the differential abundance analysis, lipids not detected in a sample were treated as missing and not imputed. All *P* values are two-sided. The sample size and the test used are specified in each figure legend, and exact *P* values are provided in the Source Data file.

### Reporting summary

Further information on research design is available in the Nature Portfolio Reporting Summary linked to this article.

## Supplementary information

Supplementary Information

Description of Additional Supplementary Files

Supplementary Movie 1

Supplementary Movie 2

Supplementary Movie 3

Supplementary Movie 4

Supplementary Movie 5

Reporting Summary

Transparent Peer Review file

## Source data

Source data

## Data Availability

The data supporting the findings of this study are available within the article and its Supplementary Information files. The Source Data file comprises the numerical raw data underlying all main and Supplementary Fig. panels, organised by figure, with heatmap source values and uncropped blot scans in dedicated worksheets. The lipidomics datasets generated in this study, comprising absolute (pmol) lipid class and lipid species quantifications for both mass spectrometry experiments, have been deposited in figshare^52^ under accession code https://doi.org/10.6084/m9.figshare.33974230. Source data are provided with this paper.

## Code availability

Single-cell image quantification was performed with a custom analysis pipeline written in Python using the scikit-image library, as described in the Methods. All parameters and processing steps are reported in full in the Methods to allow the analysis to be reproduced. The custom analysis script used for single-cell image quantification is available in Zenodo^53^ under accession code https://doi.org/10.5281/zenodo.22896752. All other analyses used standard, previously published software packages as described in the Methods.
