# Supplementary material for: Phospholipase D1 activates NAD kinase fuelling neutrophil extracellular trap formation
Source: Nat Commun. 2026 Nov 6;17:11497. doi: 10.1038/s41467-026-79319-6 (PMC13743754; doi:10.1038/s41467-026-79319-6)

Supplementary Information

Phospholipase D1 activates NAD kinase fuelling Neutrophil Extracellular Trap formation

Rahaf Albarghash[1], Sergio San-Miguel[1], Arturo Zychlinsky[1]* and Dhiren Ferise Patel[1]*

[1]Department of Cellular Microbiology, Max Planck Institute for Infection Biology, Berlin, Germany.

*Corresponding author: patel@mpiib-berlin.mpg.de and zychlinsky@mpiib-berlin.mpg.de

Contents

Supplementary Figure 1: Lipidomic remodelling during neutrophil activation

Supplementary Figure 2: PLD1 inhibition reduces NET formation

Supplementary Figure 3: PLD1 activity increases during neutrophil activation and promotes phosphoinositide synthesis

Supplementary Figure 4: PLD1 inhibition alters PI and phosphoinositide synthesis during neutrophil activation

Supplementary Figure 5: PA regulates PIP5K1 activity and phosphoinositide synthesis

Supplementary Figure 6: PLD1 regulates ROS production and metabolic activity during neutrophil activation

Supplementary Figure 7: Temporal extension of Figure 4A–C

Supplementary Figure 8: PI4K inhibition impairs NETosis and NADK activity downstream of PLD1

Supplementary Figure 9: *E. coli* phagocytosis gating strategy

Uncropped scans of the dot blots shown in Supplementary Figure 5B and 5C

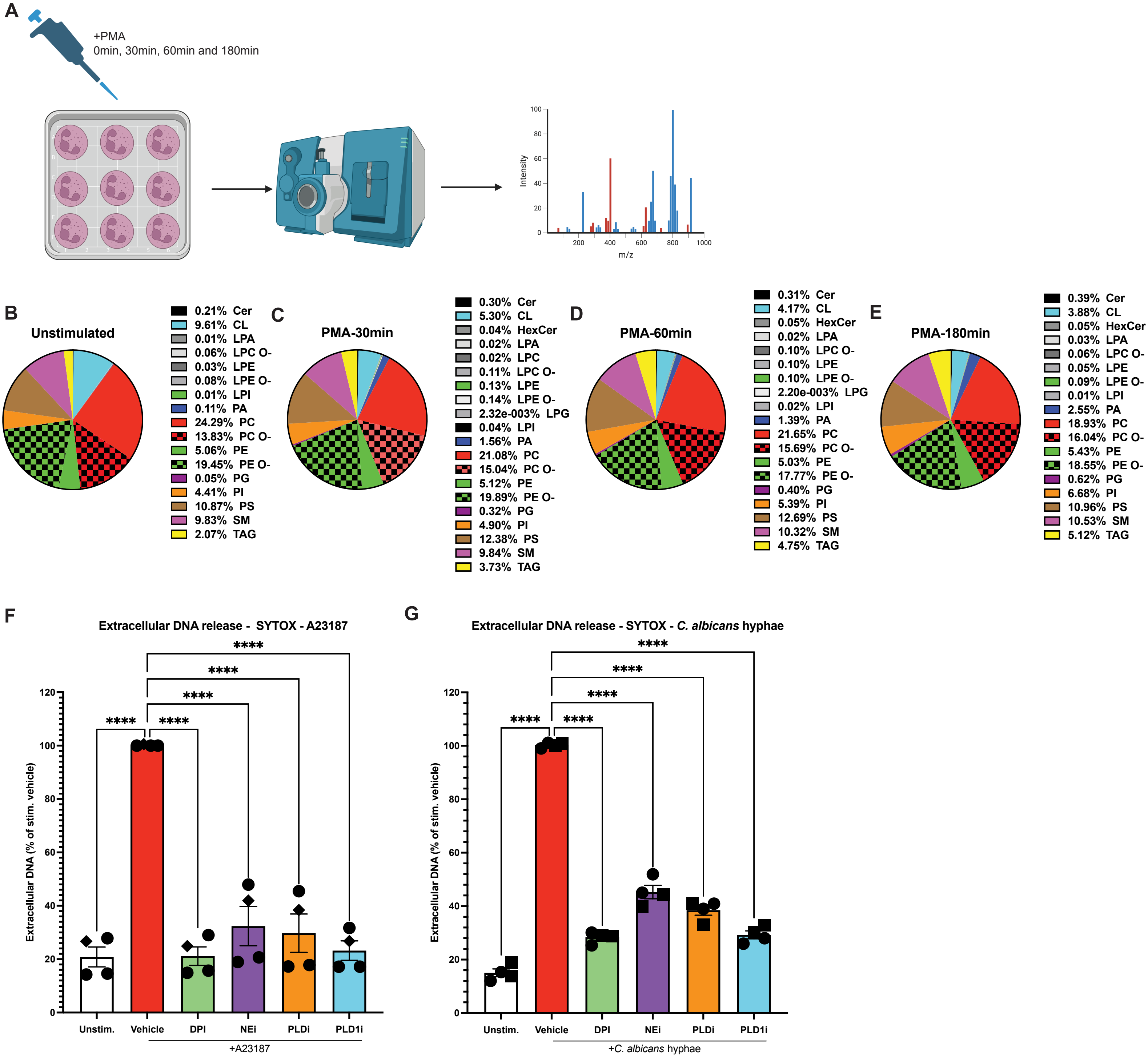
A
+PMA
0min, 30min, 60min and 180min
Intensity
m/z
B
Unstimulated
0.21% Cer
9.61% CL
0.01% LPA
0.06% LPC O-
0.03% LPE
0.08% LPE O-
0.01% LPI
0.11% PA
24.29% PC
13.83% PC O-
5.06% PE
19.45% PE O-
0.05% PG
4.41% PI
10.87% PS
9.83% SM
2.07% TAG
C
PMA-30min
0.30% Cer
5.30% CL
0.04% HexCer
0.02% LPA
0.02% LPC
0.11% LPC O-
0.13% LPE
0.14% LPE O-
2.32e-003% LPG
0.04% LPI
1.56% PA
21.08% PC
15.04% PC O-
5.12% PE
19.89% PE O-
0.32% PG
4.90% PI
12.38% PS
9.84% SM
3.73% TAG
D
PMA-60min
0.31% Cer
4.17% CL
0.05% HexCer
0.02% LPA
0.10% LPC O-
0.10% LPE
0.10% LPE O-
2.20e-003% LPG
0.02% LPI
1.39% PA
21.65% PC
15.69% PC O-
5.03% PE
17.77% PE O-
0.40% PG
5.39% PI
12.69% PS
10.32% SM
4.75% TAG
E
PMA-180min
0.39% Cer
3.88% CL
0.05% HexCer
0.03% LPA
0.06% LPC O-
0.05% LPE
0.09% LPE O-
0.01% LPI
2.55% PA
18.93% PC
16.04% PC O-
5.43% PE
18.55% PE O-
0.62% PG
6.68% PI
10.96% PS
10.53% SM
5.12% TAG
F
Extracellular DNA release - SYTOX - A23187
Extracellular DNA (% of stim. vehicle)
****
Unstim.
Vehicle
DPI
NEi
PLDi
PLD1i
+A23187
G
Extracellular DNA release - SYTOX - C. albicans hyphae
Extracellular DNA (% of stim. vehicle)
****
Unstim.
Vehicle
DPI
NEi
PLDi
PLD1i
+C. albicans hyphae

Supplementary Figure 1: Lipidomic remodelling during neutrophil activation. **A**. Schematic representation of the experimental setup where lysates of neutrophils stimulated with PMA for 30, 60, or 180 min were analysed with lipidomics. Created in BioRender. Patel, D. (2026) https://BioRender.com/7rzfkfm. B–E. Pie chart of the percentage (mol % of total lipid) of different lipid classes in naïve (**B**) as well as neutrophils stimulated with PMA for 30 (**C**), 60 (**D**) or 180 min (**E**). F–G. Extracellular DNA release, used here as a proxy for NETs, measured fluorometrically with cell-impermeable dye, SYTOX green, after stimulation with A23187 (**F**) or *C. albicans* hyphae (**G**) in the presence of vehicle, DPI, NEi, PLDi or PLD1i, for 240 min. B–E. Data represent the mean of $N = 3$ donors, each the mean of 3 technical replicates. F–G. Data shown as mean ± s.e.m., $N = 4$ donors. Each point is one donor, plotted as the mean of 2 technical replicates and shown with a different symbol. F–G. Statistical significance was determined by one-way ANOVA with Bonferroni's multiple comparisons test. ****$P < 0.0001$. Exact P values are provided in the Source Data file. Source data are provided as a Source Data file.

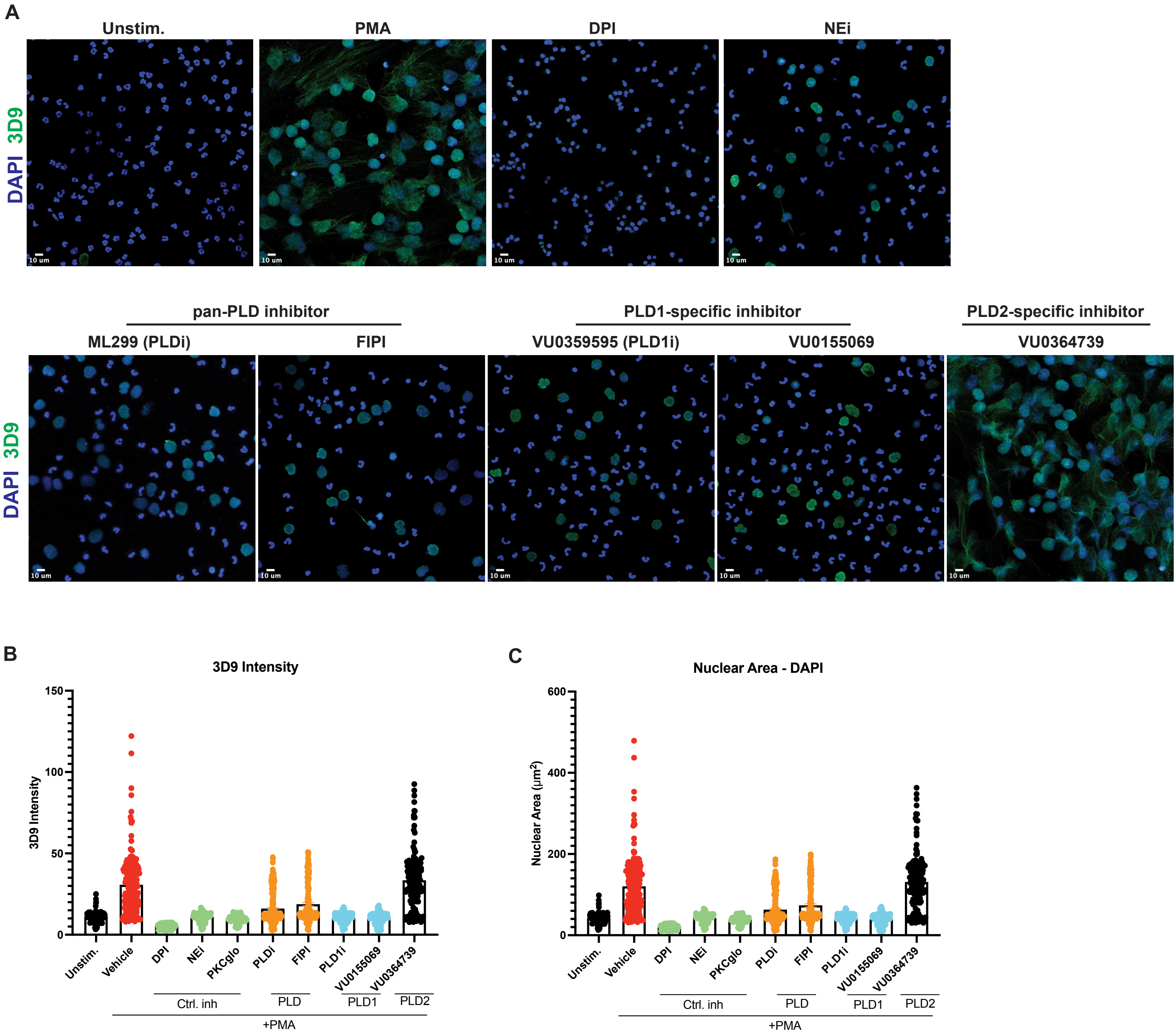
A
Unstim.
PMA
DPI
NEi
DAPI 3D9
pan-PLD inhibitor
PLD1-specific inhibitor
PLD2-specific inhibitor
ML299 (PLDi)
FIPI
VU0359595 (PLD1i)
VU0155069
VU0364739
10 um
B
3D9 Intensity
3D9 Intensity
150
100
50
0
C
Nuclear Area - DAPI
Nuclear Area (μm²)
600
400
200
0
Unstim.
Vehicle
DPI
NEi
PKCglo
PLDi
FIPI
PLD1i
VU0155069
VU0364739
Ctrl. inh
PLD
PLD1
PLD2
+PMA

Supplementary Figure 2: PLD1 inhibition reduces NET formation. **A**. Representative confocal microscopy images of neutrophils pre-incubated with the indicated inhibitors before 4 hours of PMA stimulation. The cells were then fixed and stained with DAPI (blue) and the NET-specific antibody, 3D9 (green). **B**. 3D9 intensity determined by Analyze Particles (Fiji) analysis of representative images shown in A. **C**. Nuclear area determined by Analyze Particles (Fiji) analysis of the images shown in A. Representative images from two donors. Scale bar, 10 µm. B–C. Data shown as mean ± s.e.m., $N = 2$ donors, each data point representative of individual cells (n = cells per condition, donor 1/donor 2): Unstim. 111/94, Vehicle 101/107, DPI 105/92, NEi 70/83, PKCglo 37/38, PLDi 104/130, FIPI 61/107, PLD1i 109/97, VU0155069 119/174, VU0364739 59/96. No statistical comparisons were performed for these panels. Source data are provided as a Source Data file.

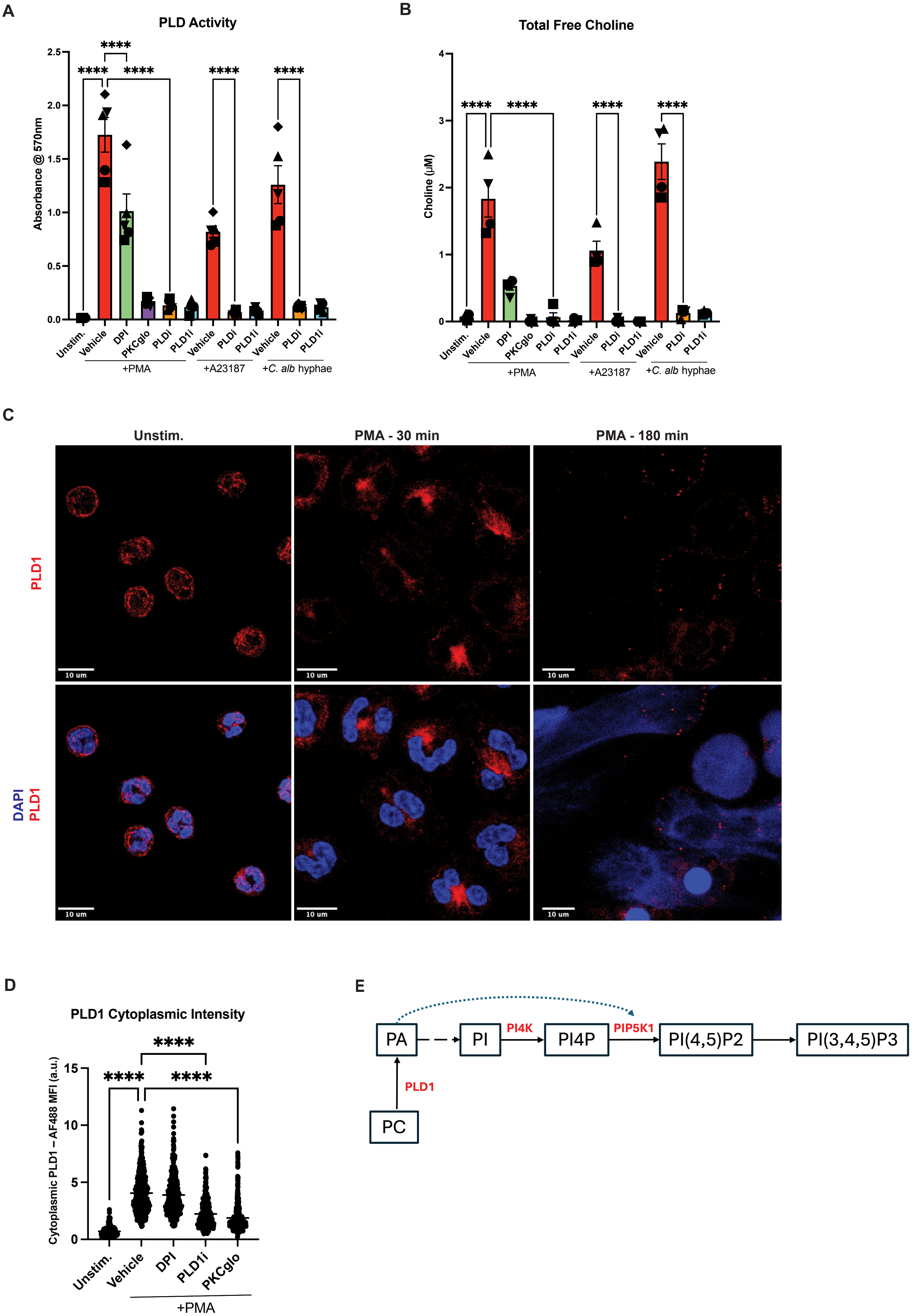
A
PLD Activity
Absorbance @ 570nm
2.5
2.0
1.5
1.0
0.5
0.0
****
****
****
****
****
Unstim.
Vehicle
DPI
PKCglo
PLDi
PLD1i
Vehicle
PLDi
PLD1i
Vehicle
PLDi
PLD1i
+PMA
+A23187
+C. alb hyphae
B
Total Free Choline
Choline (μM)
4
3
2
1
0
****
****
****
****
Unstim.
Vehicle
DPI
PKCglo
PLDi
PLD1i
Vehicle
PLDi
PLD1i
Vehicle
PLDi
PLD1i
+PMA
+A23187
+C. alb hyphae
C
Unstim.
PMA - 30 min
PMA - 180 min
PLD1
DAPI PLD1
10 um
D
PLD1 Cytoplasmic Intensity
Cytoplasmic PLD1 – AF488 MFI (a.u.)
15
10
5
0
****
****
****
Unstim.
Vehicle
DPI
PLD1i
PKCglo
+PMA
E
PA
PI
PI4K
PI4P
PIP5K1
PI(4,5)P2
PI(3,4,5)P3
PLD1
PC

Supplementary Figure 3: PLD1 activity increases during neutrophil activation and promotes phosphoinositide synthesis. **A**. PLD enzymatic activity measured with a fluorogenic substrate in neutrophil lysates that were pre-incubated with the indicated inhibitors for 30 min and stimulated with PMA or A23187 or *C. albicans* hyphae. **B**. Free choline concentration in neutrophil lysates that were pre-incubated with the indicated inhibitors for 30 min and stimulated with PMA or A23187 or *C. albicans* hyphae. **C**. Representative confocal images of neutrophils stimulated with PMA for the indicated timepoints, fixed and stained with DAPI (blue) and PLD1 (red). **D**. Confocal images quantification of cells stained with an antibody recognising PLD1. Mean cytoplasmic fluorescence intensity of the AlexaFluor 488-labelled secondary. **E**. Scheme showing how PA affects the synthesis of phosphoinositides. Previous studies suggest, PA is converted into phosphatidylinositol (PI, dashed lines), which serves as the substrate for phosphatidylinositol 4-kinase (PI4K) to synthesise phosphatidylinositol 4-phosphate (PI4P). Phosphatidylinositol-4-phosphate 5-kinase 1 (PIP5K1) then converts PI4P into phosphatidylinositol 4,5-bisphosphate ($PI(4,5)P_2$ or PIP_2). PA, independent of PI conversion, promotes PIP_2 synthesis by boosting PIP5K1 activity. Finally, PIP_2 can be converted into PIP_3. A. Data shown as mean ± s.e.m., $N = 5$ donors. Each point is one donor, plotted as the mean of 2 technical replicates and shown with a different symbol. B. Data shown as mean ± s.e.m., $N = 4$ donors. Each point is one donor, plotted as the mean of 2 technical replicates and shown with a different symbol. C. Representative images from two donors. Scale bar, 10 µm. D. Data shown as mean ± s.e.m., $N = 2$ donors, each data point representative of individual cells (n = cells per condition, donor 1/donor 2): Unstim. 141/85, PMA 229/209, DPI 155/217, PKCglo 197/183, PLD1i 164/121. A–B, D. Statistical significance was determined by one-way ANOVA with Bonferroni's multiple comparisons test. ****$P < 0.0001$. a.u., arbitrary units. Exact P values are provided in the Source Data file. Source data are provided as a Source Data file.

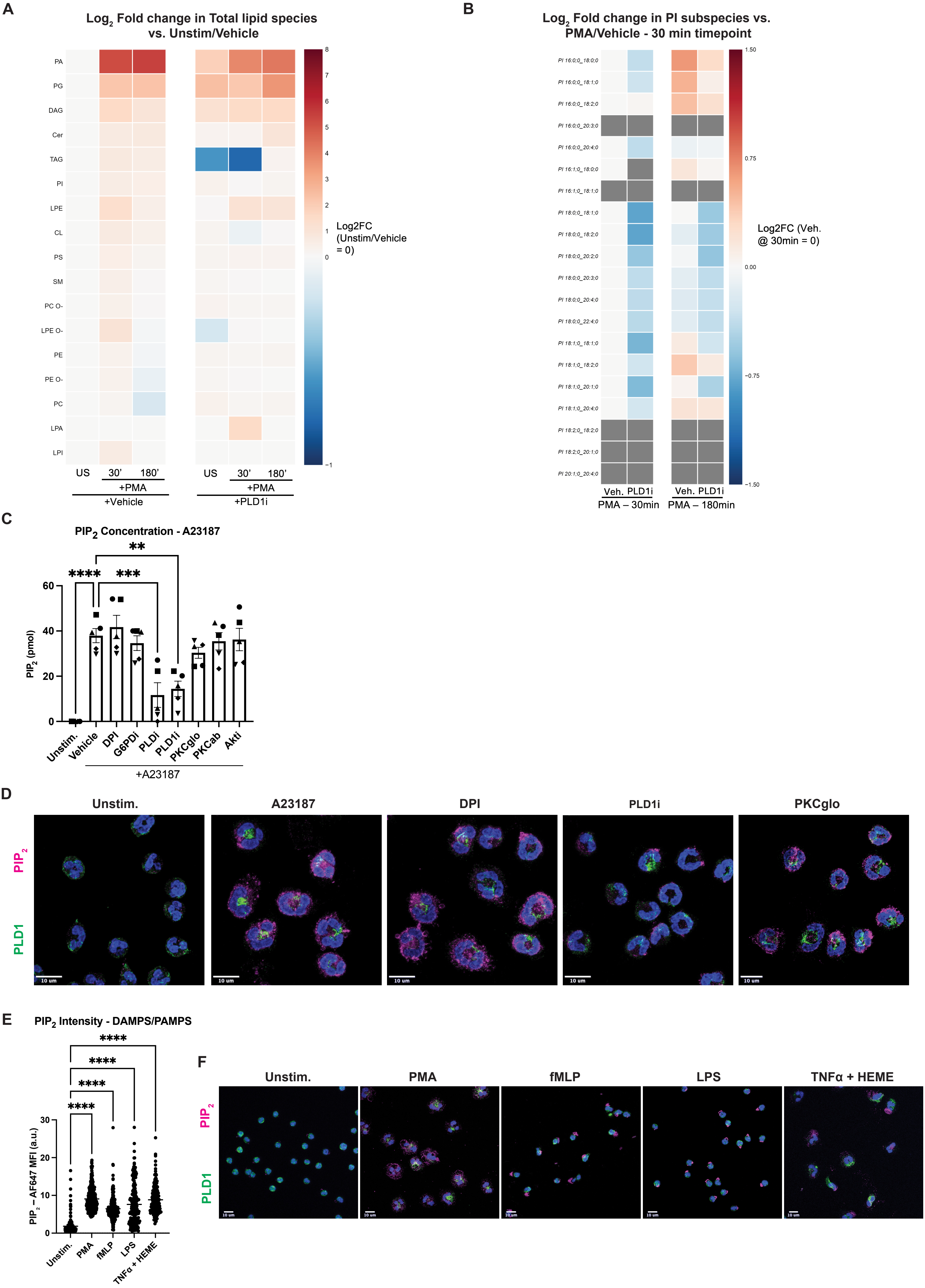
A
Log2 Fold change in Total lipid species vs. Unstim/Vehicle
PA
PG
DAG
Cer
TAG
PI
LPE
CL
PS
SM
PC O-
LPE O-
PE
PE O-
PC
LPA
LPI
US
30'
180'
+PMA
+Vehicle
+PLD1i
Log2FC (Unstim/Vehicle = 0)
B
Log2 Fold change in PI subspecies vs. PMA/Vehicle - 30 min timepoint
Log2FC (Veh. @ 30min = 0)
Veh.
PLD1i
PMA – 30min
PMA – 180min
C
PIP2 Concentration - A23187
PIP2 (pmol)
Unstim.
Vehicle
DPI
G6PDi
PLDi
PLD1i
PKCglo
PKCab
Akti
+A23187
D
Unstim.
A23187
DPI
PLD1i
PKCglo
PIP2
PLD1
E
PIP2 Intensity - DAMPS/PAMPS
PIP2 – AF647 MFI (a.u.)
Unstim.
PMA
fMLP
LPS
TNFα + HEME
F
Unstim.
PMA
fMLP
LPS
TNFα + HEME
PIP2
PLD1

Supplementary Figure 4: PLD1 inhibition alters PI and phosphoinositide synthesis during neutrophil activation. **A**. Heatmap showing log_2 fold change (log_2FC) in major lipid classes in lysates of human neutrophils stimulated with PMA for 30 or 180 min, relative to unstimulated (US) cells, including phosphatidylglycerol (PG), diacylglycerol (DAG), lysophosphatidylethanolamine (LPE), lysophosphatidic acid (LPA), ceramides (Cer) and phosphatidylinositol (PI). Data represent mean log_2 fold change from N = 3 donors. **B**. Heatmap showing log_2 fold change in phosphatidylinositol (PI) subspecies in lysates of neutrophils treated with PLD1i or vehicle and stimulated with PMA for 30 or 180 min, normalised to vehicle-treated PMA-30 min cells. Grey boxes indicate undetected species. Data represent mean log_2 fold change from N = 3 donors. **C**. PIP_2 concentrations determined by ELISA in lysates of neutrophils pre-incubated with either vehicle or the indicated inhibitors and then stimulated for 10 min with A23187. **D**. Representative confocal microscopy images of neutrophils pre-incubated in the indicated conditions and stained the DNA with DAPI (blue), PIP_2 (magenta) and PLD1 (green) after stimulation with A23187. **E**. Confocal images quantification of cells stained with an antibody recognising PIP_2 taken from neutrophils stimulated with PMA or DAMPs/PAMPs: fMLP, LPS or TNFα-primed heme. Mean fluorescence intensity of the Alexa 647-labelled secondary. **F**. Representative confocal images of the DAMP/PAMP conditions stained with DAPI (blue), PIP_2 (magenta) and PLD1 (green). C. Data shown as mean ± s.e.m., N = 5 donors. Each point is one donor, plotted as the mean of 2 technical replicates and shown with a different symbol. D. Representative images from two donors. Scale bar, 10 µm. E. Data shown as mean ± s.e.m., N = 2 donors, each data point representative of individual cells (n = cells per condition, donor 1/donor 2): Unstim. 47/132, PMA 172/159, fMLP 106/127, LPS 79/129, TNFα-primed heme 110/101. F. Representative images from two donors. Scale bar, 10 µm. C, E. Statistical significance was determined by one-way ANOVA with Bonferroni's multiple comparisons test. $^{**}P < 0.01$, $^{***}P < 0.001$ and $^{****}P < 0.0001$. a.u., arbitrary units. Exact P values are provided in the Source Data file. Source data are provided as a Source Data file.

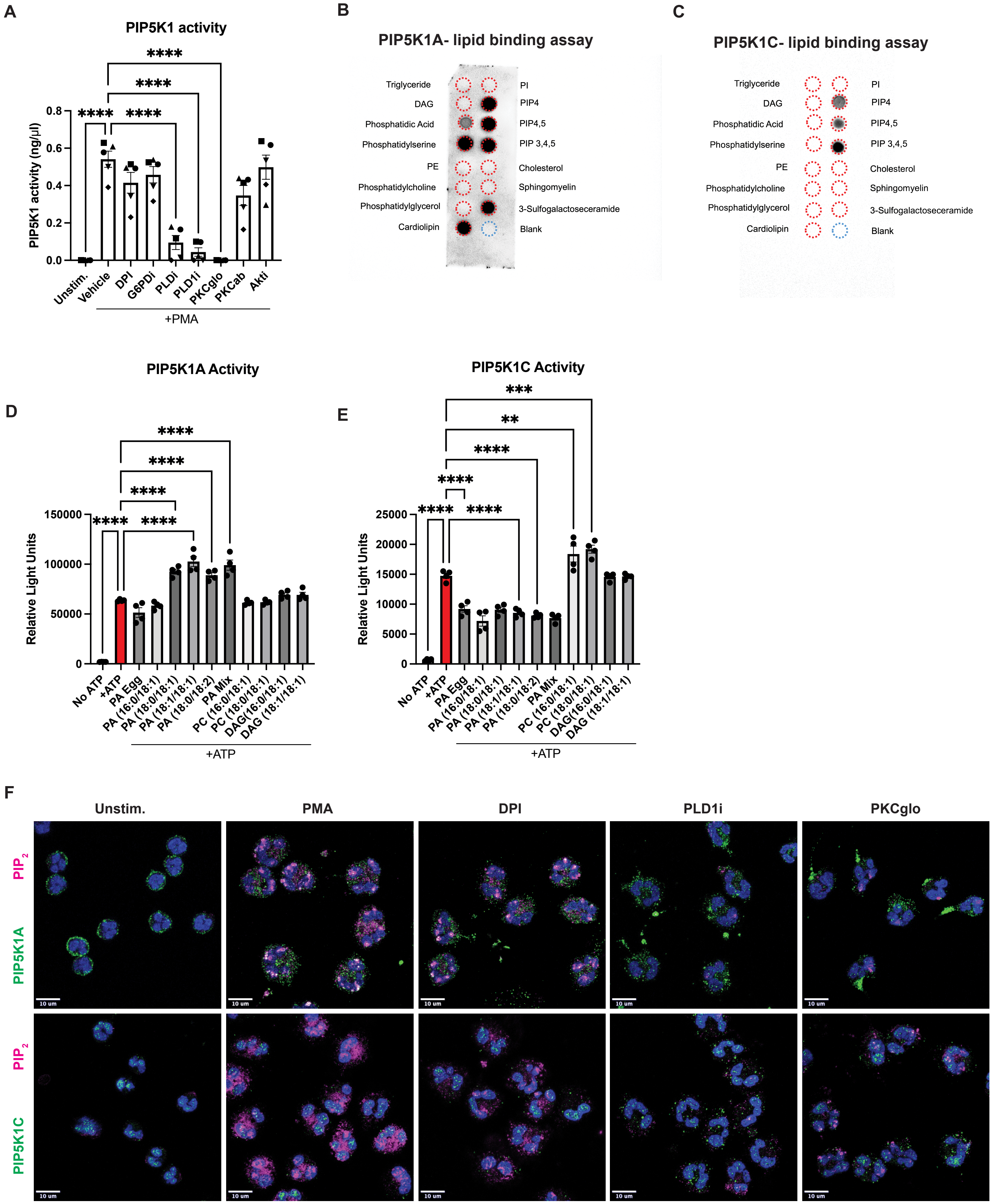

A
PIP5K1 activity
PIP5K1 activity (ng/µl)
Unstim.
Vehicle
DPI
G6PDi
PLDi
PLD1i
PKCglo
PKCab
Akti
+PMA
B
PIP5K1A- lipid binding assay
C
PIP5K1C- lipid binding assay
Triglyceride
DAG
Phosphatidic Acid
Phosphatidylserine
PE
Phosphatidylcholine
Phosphatidylglycerol
Cardiolipin
PI
PIP4
PIP4,5
PIP 3,4,5
Cholesterol
Sphingomyelin
3-Sulfogalactoseceramide
Blank
PIP5K1A Activity
PIP5K1C Activity
D
E
Relative Light Units
No ATP
+ATP
PA Egg
PA (16:0/18:1)
PA (18:0/18:1)
PA (18:1/18:1)
PA (18:0/18:2)
PA Mix
PC (16:0/18:1)
PC (18:0/18:1)
DAG(16:0/18:1)
DAG (18:1/18:1)
+ATP
F
Unstim.
PMA
DPI
PLD1i
PKCglo
PIP2
PIP5K1A
PIP5K1C
10 um

Supplementary Figure 5: PA regulates PIP5K1 activity and phosphoinositide synthesis. **A**. PIP5K1 activity in lysates of neutrophils pre-incubated with the indicated inhibitors for 30 min before PMA stimulation. **B**. Dot-blot showing that recombinant PIP5K1A binds to anionic lipids; PA, phosphatidylserine (PS), cardiolipin, and multiple phosphoinositides (PI, PI4P, PIP_2, and PIP_3), as well as sulphogalactosylceramide. **C**. Dot-blot showing that recombinant PIP5K1C binds predominantly to phosphoinositides (PI, PI4P, PIP_2, and PIP_3), and interacts poorly with other anionic lipids such as PA, PS, or cardiolipin. **D**. Recombinant PIP5K1A activity determined using an ADP-glo assay in the presence of the indicated lipids as well as ATP. **E**. Recombinant PIP5K1C activity determined using an ADP-glo assay in the presence of the indicated lipids as well as ATP. **F**. Representative confocal microscopy images of PMA-, DPI-, PLD1i- or PKCglo-treated neutrophils stained with DAPI (blue), PIP_2 (magenta) and either PIP5K1A (green, top row) or PIP5K1C (green, bottom row). A. Data shown as mean ± s.e.m., $N = 5$ donors. Each point is one donor, plotted as the mean of 2 technical replicates and shown with a different symbol. B–C. Representative dot blots from 2 independent experiments. D–E. Data shown as mean ± s.e.m., $n = 4$ experiments. Each point is one experiment, plotted as the mean of 2 technical replicates. F. Representative images from two donors. Scale bar, 10 µm. A, D–E. Statistical significance was determined by one-way ANOVA with Bonferroni's multiple comparisons test. $^{**}P < 0.01$, $^{***}P < 0.001$ and $^{****}P < 0.0001$. Exact *P* values are provided in the Source Data file. Source data are provided as a Source Data file.

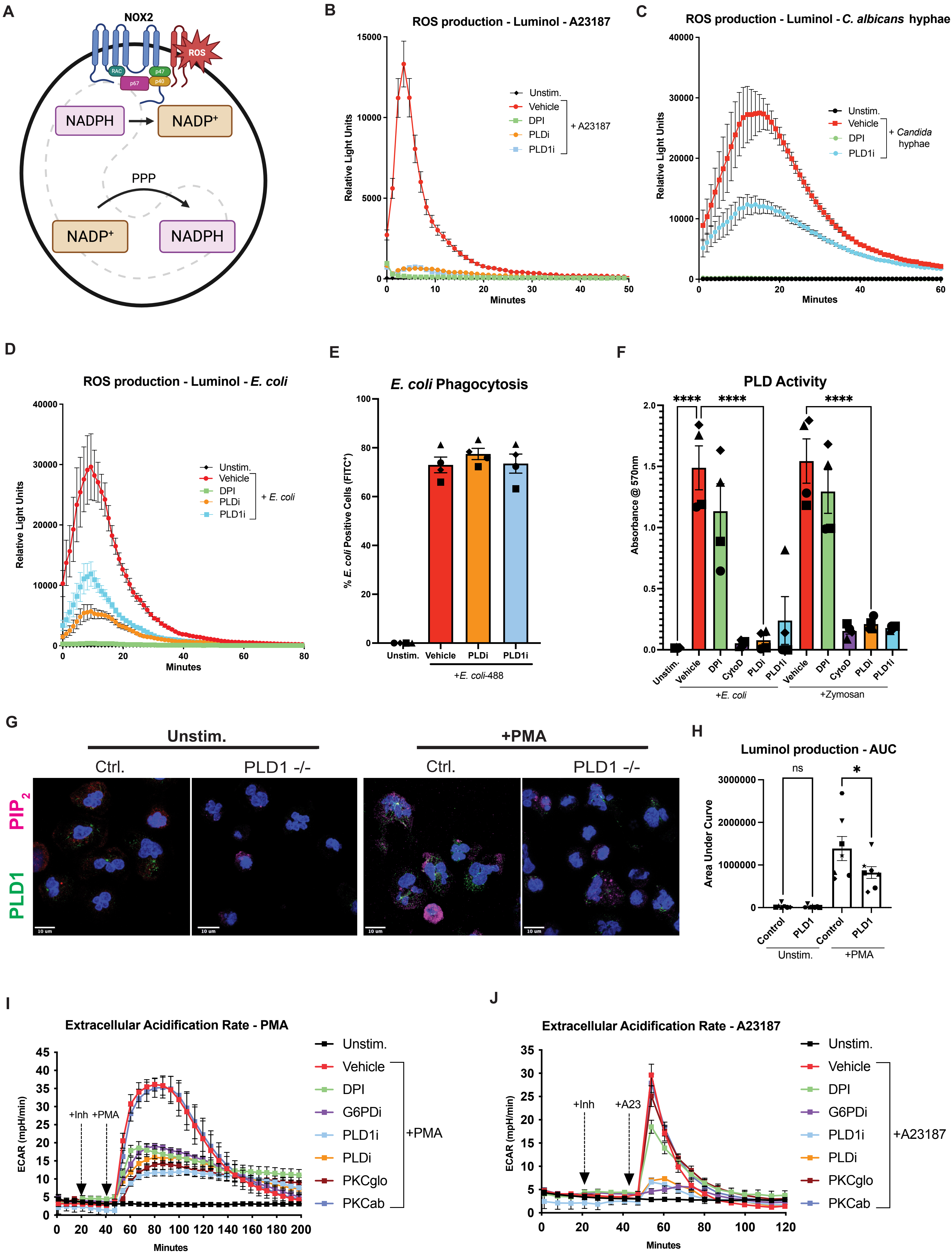

A
NOX2
ROS
RAC
p47
p67
p40
NADPH
NADP+
PPP
NADP+
NADPH
B
ROS production - Luminol - A23187
Relative Light Units
Minutes
Unstim.
Vehicle
DPI
PLDi
PLD1i
+ A23187
C
ROS production - Luminol - C. albicans hyphae
Relative Light Units
Minutes
Unstim.
Vehicle
DPI
PLD1i
+ Candida hyphae
D
ROS production - Luminol - E. coli
Relative Light Units
Minutes
Unstim.
Vehicle
DPI
PLDi
PLD1i
+ E. coli
E
E. coli Phagocytosis
% E. coli Positive Cells (FITC+)
Unstim.
Vehicle
PLDi
PLD1i
+E. coli-488
F
PLD Activity
Absorbance @ 570nm
****
****
****
Unstim.
Vehicle
DPI
CytoD
PLDi
PLD1i
+E. coli
+Zymosan
G
Unstim.
+PMA
Ctrl.
PLD1 -/-
Ctrl.
PLD1 -/-
PIP2
PLD1
10 um
H
Luminol production - AUC
Area Under Curve
ns
*
Control
PLD1
Unstim.
+PMA
I
Extracellular Acidification Rate - PMA
ECAR (mpH/min)
Minutes
+Inh
+PMA
Unstim.
Vehicle
DPI
G6PDi
PLD1i
PLDi
PKCglo
PKCab
+PMA
J
Extracellular Acidification Rate - A23187
ECAR (mpH/min)
Minutes
+Inh
+A23
Unstim.
Vehicle
DPI
G6PDi
PLD1i
PLDi
PKCglo
PKCab
+A23187

Supplementary Figure 6: PLD1 regulates ROS production and metabolic activity during neutrophil activation. **A**. Schematic of neutrophil ROS production (PPP, pentose phosphate pathway). Created in BioRender. Patel, D. (2026) https://BioRender.com/jl448tb. B–D. ROS production by neutrophils pre-incubated with the indicated inhibitors for 30 min upon stimulation with A23187 (**B**), *C. albicans* hyphae (**C**) or *Escherichia coli* (**D**) determined by a luminol assay. **E**. Flow cytometry analysis of AF488-tagged E. coli phagocytosed by neutrophils with the indicated inhibitors. **F**. PLD activity in neutrophil lysates exposed to *E. coli* or zymosan with the indicated inhibitors, (Cytochalasin D=CytoD). PLD1 was knocked out using CRISPR/Cas9 in neutrophil-like cells differentiated from human blood $CD34^+$ stem cells. Control (Ctrl, cells transfected with a control sgRNA) and PLD1-/- cells were stimulated with PMA. **G**. Representative confocal images of indicated neutrophil-like cells stained with PLD1 (green) and PIP_2 (magenta). **H**. Area under curve (AUC) of a ROS luminol assay time course (150 min) in the indicated stem-cell-derived neutrophils. **I**. Extracellular acidification rate (ECAR) of neutrophils stimulated with PMA in the presence of the indicated inhibitors, determined by seahorse assay. **J**. ECAR of neutrophils stimulated with A23187 in the presence of the indicated inhibitors. B–C, I–J. Data shown as mean ± s.e.m., $N = 5$ donors, 2 technical replicates per donor. D. Data shown as mean ± s.e.m., $N = 4$ donors, 2 technical replicates per donor. E–F. Data shown as mean ± s.e.m., $N = 4$ donors. Each point is one donor, plotted as the mean of 2 technical replicates and shown with a different symbol. G. Representative images from two donors. Scale bar, 10 µm. H. Data shown as mean ± s.e.m., $N = 7$ donors. Each point is one donor (mean of 2 technical replicates), shown with a different symbol. F. Statistical significance was determined by one-way ANOVA with Bonferroni's multiple comparisons test. H. Statistical significance was determined by repeated-measures one-way ANOVA (matched by donor) with Holm–Šídák's multiple comparisons test. ns, not significant, $*P < 0.05$ and $****P < 0.0001$. Exact *P* values are provided in the Source Data file. Source data are provided as a Source Data file.

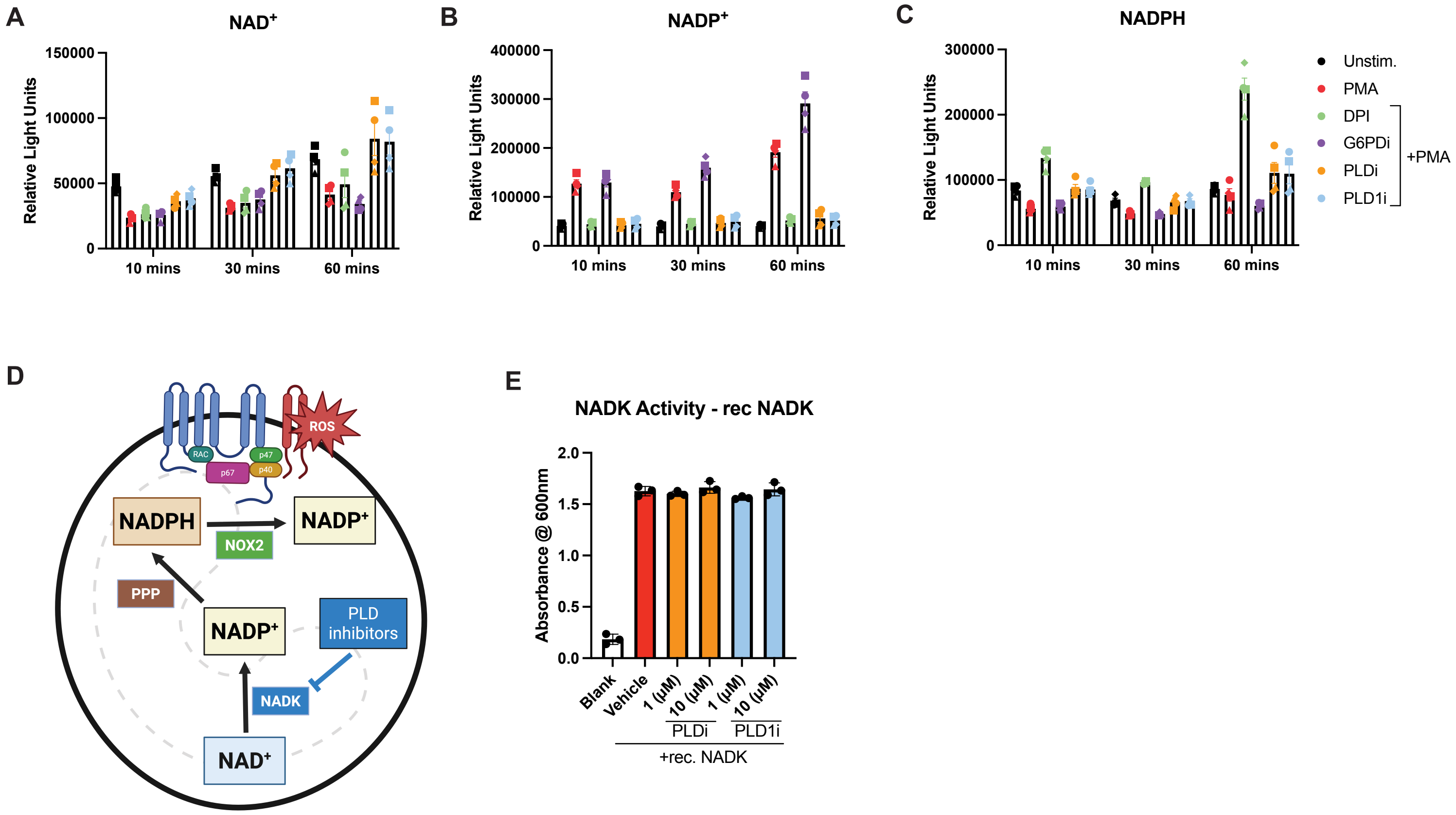
A
NAD+
Relative Light Units
150000
100000
50000
0
10 mins
30 mins
60 mins
B
NADP+
400000
300000
200000
100000
C
NADPH
300000
200000
100000
Unstim.
PMA
DPI
G6PDi
PLDi
PLD1i
+PMA
D
ROS
RAC
p47
p67
p40
NADPH
NADP+
NOX2
PPP
NADP+
PLD inhibitors
NADK
NAD+
E
NADK Activity - rec NADK
Absorbance @ 600nm
2.0
1.5
1.0
0.5
0.0
Blank
Vehicle
1 (μM)
10 (μM)
1 (μM)
10 (μM)
PLDi
PLD1i
+rec. NADK

Supplementary Figure 7: Temporal extension of Figure 4A–C. NAD^+ (**A**), $NADP^+$ (**B**) and NADPH (**C**) concentrations in neutrophil lysates at 10, 30 and 60 min after stimulation, in the presence of NOX2 (DPI), PPP (G6PDi) and PLD inhibitors. The 30-min time point corresponds to the snapshot shown in Figure 4A–C. **D**. Schematic of NAD^+, $NADP^+$ and NADPH metabolism in neutrophils during activation and ROS production. Neutrophils, upon stimulation, convert NAD^+ to $NADP^+$ via NAD kinase (NADK). $NADP^+$ is reduced to NADPH via the oxidative pentose phosphate pathway (PPP). NADPH is used by NOX2 to synthesise ROS. Our results suggest that PLD inhibitors act upstream of the PPP and NOX2 activity by regulating NADK activity. Created in BioRender. Patel, D. (2026) https://BioRender.com/ssqj8ol. **E**. Recombinant NADK activity in the presence of PLDi or PLD1i measured as described in Methods. A–C. Data shown as mean ± s.e.m., $N = 4$ donors. Each point is one donor, plotted as the mean of 2 technical replicates and shown with a different symbol. E. Data shown as mean ± s.e.m., $n = 3$ experiments. Each point is one experiment, plotted as the mean of 2 technical replicates. No statistical comparisons were performed for these panels. Source data are provided as a Source Data file.

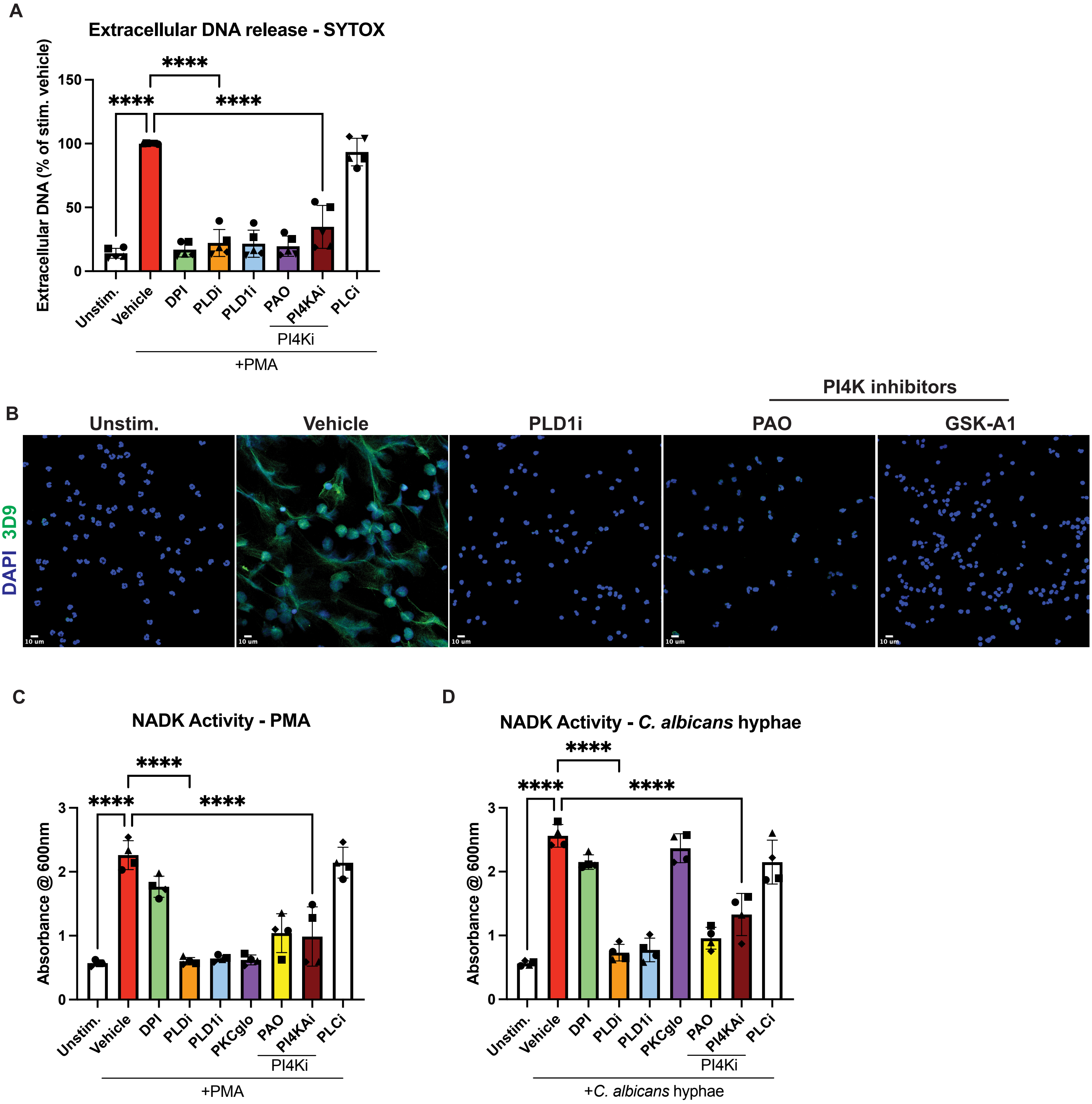
A
Extracellular DNA release - SYTOX
Extracellular DNA (% of stim. vehicle)
150
100
50
0
****
****
****
Unstim.
Vehicle
DPI
PLDi
PLD1i
PAO
PI4KAi
PLCi
PI4Ki
+PMA
B
PI4K inhibitors
Unstim.
Vehicle
PLD1i
PAO
GSK-A1
DAPI 3D9
10 um
C
NADK Activity - PMA
Absorbance @ 600nm
3
2
1
0
****
****
****
Unstim.
Vehicle
DPI
PLDi
PLD1i
PKCglo
PAO
PI4KAi
PLCi
PI4Ki
+PMA
D
NADK Activity - C. albicans hyphae
Absorbance @ 600nm
3
2
1
0
****
****
****
Unstim.
Vehicle
DPI
PLDi
PLD1i
PKCglo
PAO
PI4KAi
PLCi
PI4Ki
+C. albicans hyphae

Supplementary Figure 8: PI4K inhibition impairs NETosis and NADK activity downstream of PLD1. **A**. Extracellular DNA release, used here as a proxy for NETs, measured fluorometrically with cell-impermeable dye, SYTOX green. Neutrophils were pre-incubated with the indicated inhibitors for 30 min before PMA stimulation. **B**. Confocal microscopy images of neutrophils pre-incubated with the indicated inhibitors and stimulated with PMA for 4 hours before staining with DAPI (blue) and a NET-specific antibody, 3D9 (green). **C**. NADK activity in neutrophil lysates post PMA stimulation and pre-incubation with vehicle or the indicated inhibitors. **D**. NADK activity in neutrophil lysates post *C. albicans* hyphae stimulation and pre-incubation with vehicle or the indicated inhibitors. A. Data shown as mean ± s.e.m., $N = 5$ donors. Each point is one donor, plotted as the mean of 2 technical replicates and shown with a different symbol. C–D. Data shown as mean ± s.e.m., $N = 4$ donors. Each point is one donor, plotted as the mean of 2 technical replicates and shown with a different symbol. B. Images are representative images from 2 donors. Scale bar, 10 µm. A, C–D. Statistical significance was determined by one-way ANOVA with Bonferroni's multiple comparisons test. ****$P < 0.0001$. Exact P values are provided in the Source Data file. Source data are provided as a Source Data file.

A

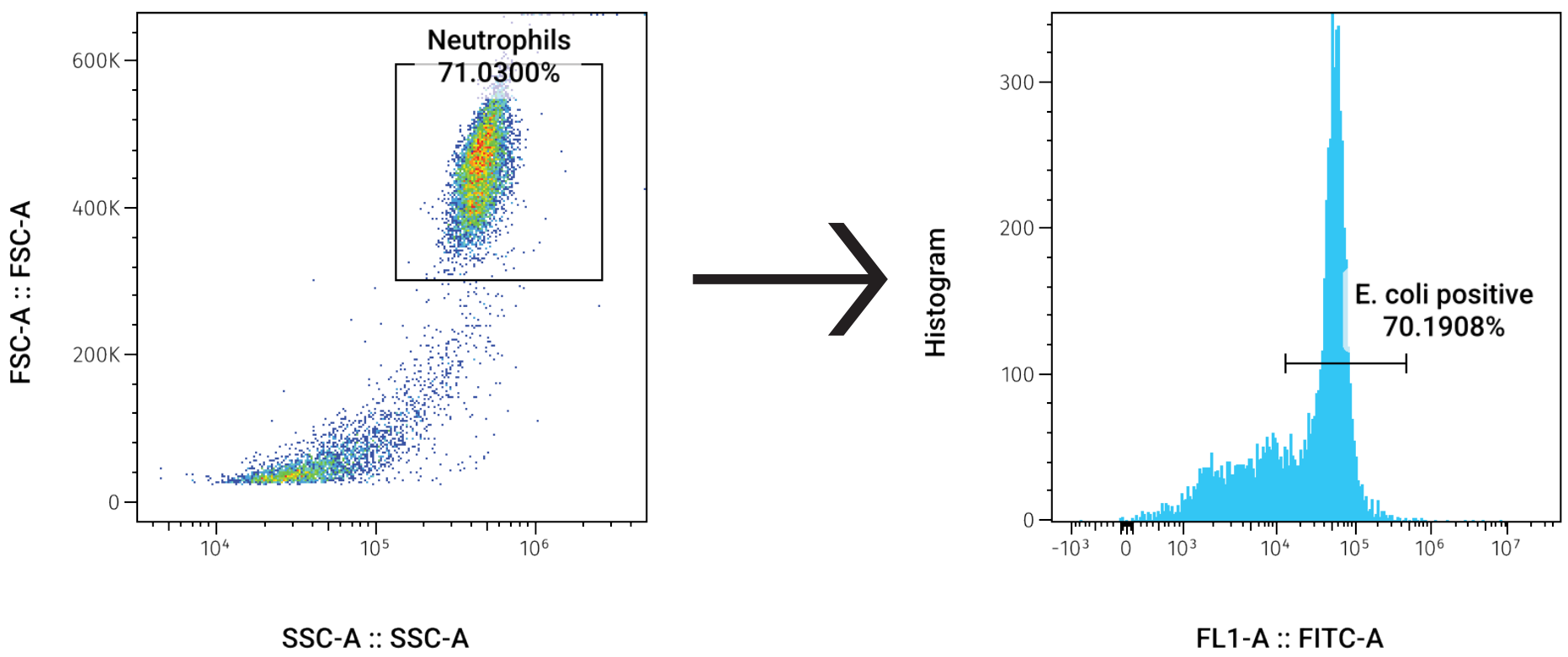
Neutrophils
71.0300%
600K
400K
200K
0
FSC-A :: FSC-A
10⁴
10⁵
10⁶
SSC-A :: SSC-A
Histogram
300
200
100
0
E. coli positive
70.1908%
-10³
0
10³
10⁴
10⁵
10⁶
10⁷
FL1-A :: FITC-A

Supplementary Figure 9: *E. coli* phagocytosis gating strategy. **A**. Representative flow cytometry gating strategy for neutrophils phagocytosing AF488-labelled *E. coli*. Neutrophils were gated on forward scatter (FSC-A) versus side scatter (SSC-A), and phagocytosis was quantified as the percentage of $AF488^+$ neutrophils determined from the fluorescence histogram (FL1-A/FITC). Representative of *N* = 4 donors.

Uncropped blot: PIP5K1A dot blot (Supplementary Figure 5B)

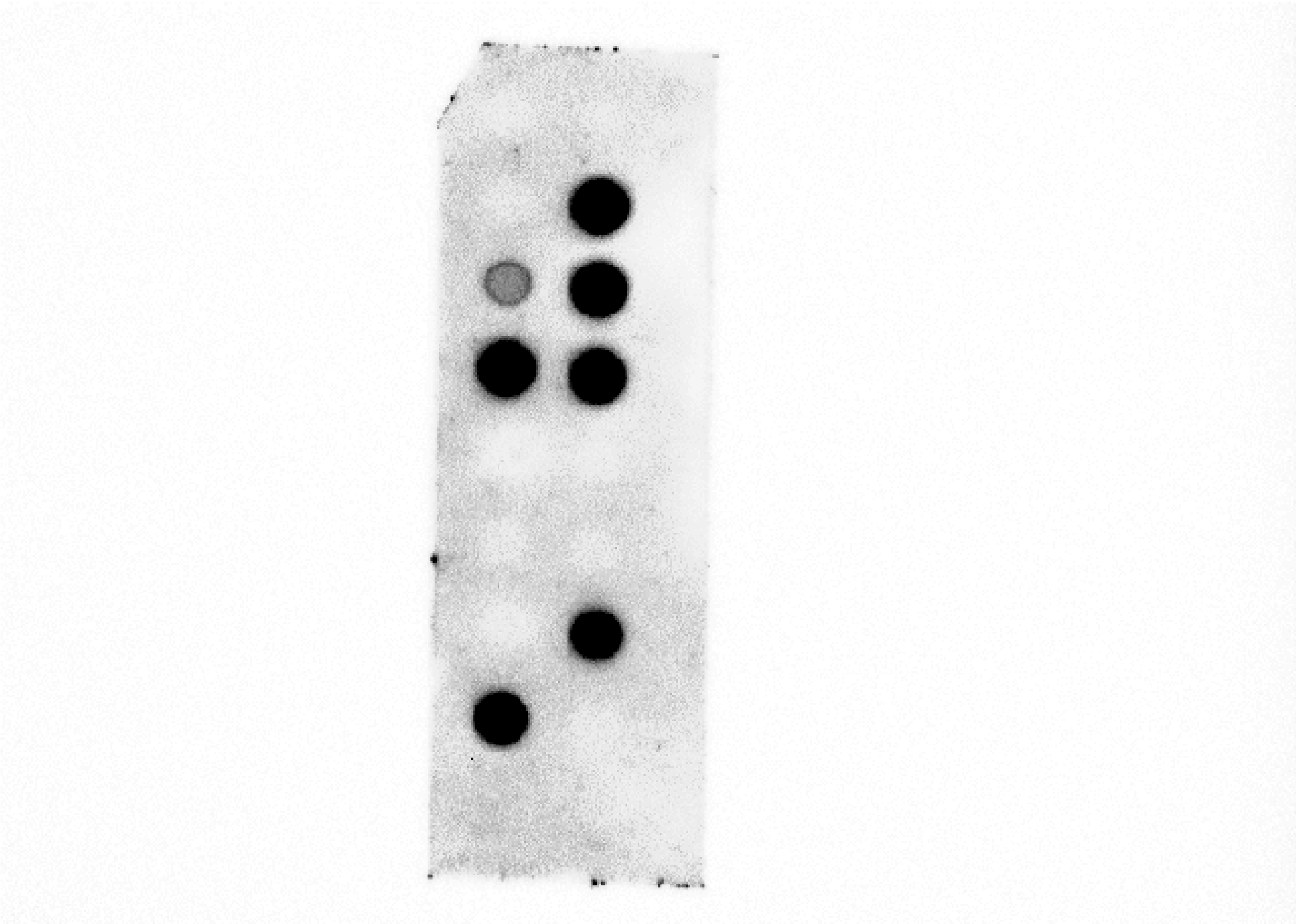

Uncropped blot: PIP5K1C dot blot (Supplementary Figure 5C)

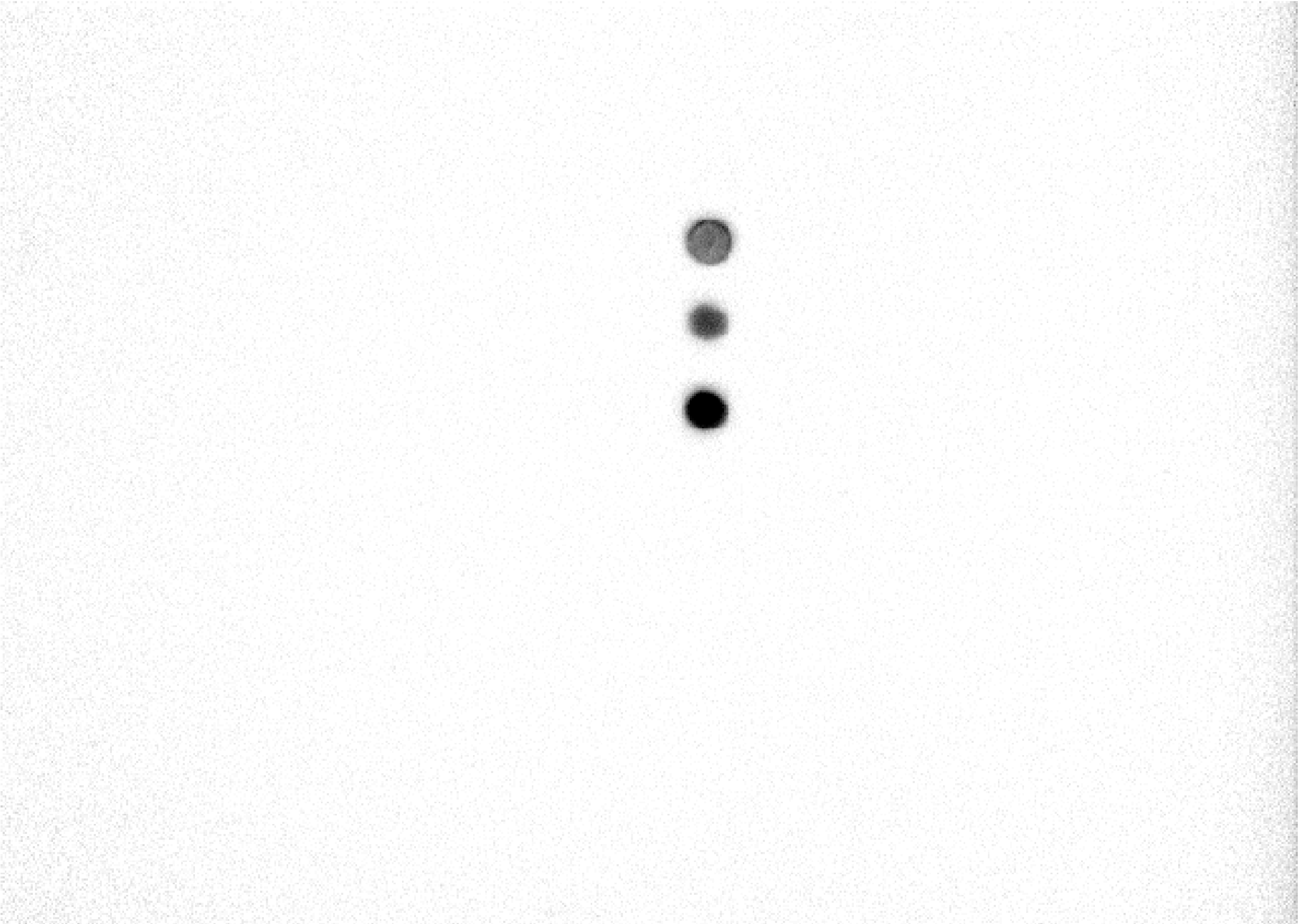

Supplement: Supplementary file 1 [file 41467_2026_79319_MOESM1_ESM.pdf]
