# Supplementary material for: Phospholipase D1 activates NAD kinase fuelling neutrophil extracellular trap formation
Source: Nat Commun. 2026 Nov 6;17:11497. doi: 10.1038/s41467-026-79319-6 (PMC13743754; doi:10.1038/s41467-026-79319-6)
Supplement: Supplementary file 2 [file 41467_2026_79319_MOESM2_ESM.pdf]

Title: Supplementary Movie 1

Description: Unstimulated human neutrophils, confocal time lapse over 4 hours, Hoechst 33342, 2 donors

Title: Supplementary Movie 2

Description: PMA-activated human neutrophils, confocal time lapse over 4 hours, Hoechst 33342, 2 donors

Title: Supplementary Movie 3

Description: DPI pre-treatment then PMA, confocal time-lapse over 4 hours, Hoechst 33342, 2 donors

Title: Supplementary Movie 4

Description: Pan-PLD inhibitor pre-treatment then PMA, confocal time-lapse over 4 hours, Hoechst 33342, 2 donors

Title: Supplementary Movie 5

Description: PLD1 inhibitor pre-treatment then PMA, confocal time-lapse over 4 hours, Hoechst 33342, 2 donors
