# Supplementary material for: Phospholipase D1 activates NAD kinase fuelling neutrophil extracellular trap formation
Source: Nat Commun. 2026 Nov 6;17:11497. doi: 10.1038/s41467-026-79319-6 (PMC13743754; doi:10.1038/s41467-026-79319-6)
Supplement: Supplementary file 3 [file 41467_2026_79319_MOESM8_ESM.pdf]

nature portfolio

Corresponding author(s): Arturo Zychlinsky, Dhiren Ferise Patel

Last updated by author(s): Jul 28, 2026

Reporting Summary

Nature Portfolio wishes to improve the reproducibility of the work that we publish. This form provides structure for consistency and transparency in reporting. For further information on Nature Portfolio policies, see our Editorial Policies and the Editorial Policy Checklist.

Statistics

For all statistical analyses, confirm that the following items are present in the figure legend, table legend, main text, or Methods section.

n/a	Confirmed	
☐	☒	The exact sample size (n) for each experimental group/condition, given as a discrete number and unit of measurement
☐	☒	A statement on whether measurements were taken from distinct samples or whether the same sample was measured repeatedly
☐	☒	The statistical test(s) used AND whether they are one- or two-sided<br>*Only common tests should be described solely by name; describe more complex techniques in the Methods section.*
☒	☐	A description of all covariates tested
☐	☒	A description of any assumptions or corrections, such as tests of normality and adjustment for multiple comparisons
☐	☒	A full description of the statistical parameters including central tendency (e.g. means) or other basic estimates (e.g. regression coefficient) AND variation (e.g. standard deviation) or associated estimates of uncertainty (e.g. confidence intervals)
☐	☒	For null hypothesis testing, the test statistic (e.g. F, t, r) with confidence intervals, effect sizes, degrees of freedom and P value noted<br>*Give P values as exact values whenever suitable.*
☒	☐	For Bayesian analysis, information on the choice of priors and Markov chain Monte Carlo settings
☒	☐	For hierarchical and complex designs, identification of the appropriate level for tests and full reporting of outcomes
☒	☐	Estimates of effect sizes (e.g. Cohen's d, Pearson's r), indicating how they were calculated

Our web collection on statistics for biologists contains articles on many of the points above.

Software and code

Policy information about availability of computer code

Data collection	LAS X (version 3.5.2.18963, Leica Microsystems) — confocal acquisition<br>Wave (version 2.4.3.7, Agilent) — Seahorse XF96<br>Gen5 (version 3.14, Agilent BioTek) — Synergy H1 plate reader<br>CytExpert (version 2.3, Beckman Coulter) — CytoFlex flow cytometer<br>Image Lab (version 5.2.1 build 11, Bio-Rad) — Gel Doc XR+<br>LipotypeXplorer (Lipotype GmbH) — lipid identification from shotgun mass spectrometry
Data analysis	Fiji (ImageJ version 1.54) — nuclear segmentation and 3D9 intensity<br>Python (version 3.12.5) with scikit-image (version 0.26.0) — PLD1 and phosphoinositide quantification<br>GraphPad Prism (version 11.0) — plotting and statistics<br>R (version 4.3.3) with limma (version 3.58.1) — lipidomics differential analysis<br>FlowJo (version 11.1.1, BD Biosciences) — flow cytometry analysis<br>TIDE (Brinkman et al. 2014) — indel quantification<br>Custom image-analysis code is deposited at Zenodo (DOI: https://doi.org/10.5281/zenodo.22896752)

For manuscripts utilizing custom algorithms or software that are central to the research but not yet described in published literature, software must be made available to editors and reviewers. We strongly encourage code deposition in a community repository (e.g. GitHub). See the Nature Portfolio guidelines for submitting code & software for further information.

Data

Policy information about availability of data

All manuscripts must include a data availability statement. This statement should provide the following information, where applicable:

- Accession codes, unique identifiers, or web links for publicly available datasets
- A description of any restrictions on data availability
- For clinical datasets or third party data, please ensure that the statement adheres to our policy

The data supporting the findings of this study are available within the article and its Supplementary Information files. Source data are provided with this paper. The Source Data file comprises the numerical raw data underlying all main and supplementary figure panels, organised by figure, with heatmap source values and uncropped blot scans in dedicated worksheets. The lipidomics datasets generated in this study, comprising absolute (pmol) lipid class and lipid species quantifications for both mass spectrometry experiments, have been deposited in figshare under accession code https://doi.org/10.6084/m9.figshare.33974230.

Research involving human participants, their data, or biological material

Policy information about studies with human participants or human data. See also policy information about sex, gender (identity/presentation), and sexual orientation and race, ethnicity and racism.

Reporting on sex and gender	Donor sex and gender were not recorded at collection, either by self-report or by assignment, and cannot be linked to individual samples. Sex and gender were therefore not considered in the study design, and sex- and gender-disaggregated analyses were not possible and none were performed. The cellular and biochemical mechanisms described are not expected to be sex or gender specific.
Reporting on race, ethnicity, or other socially relevant groupings	Donations were fully anonymised and no data on race, ethnicity or other socially relevant groupings were collected or available to the authors.
Population characteristics	Anonymous healthy adult blood donors. Donor age was not recorded and cannot be linked to individual samples. No other population characteristics were collected.
Recruitment	Blood samples and buffy coats were collected from anonymous healthy adult donors with consent and approval from the Charité Hospital ethics committee in Berlin, Germany (EA1/0104/06). Donors were not recruited for this study and no selection criteria were applied, so no self-selection bias is expected. Donors received no compensation.
Ethics oversight	Sample collection was approved by the ethics committee of Charité Hospital, Berlin, Germany (EA1/0104/06), and experiments using human samples were conducted in accordance with the Declaration of Helsinki.

Note that full information on the approval of the study protocol must also be provided in the manuscript.

Field-specific reporting

Please select the one below that is the best fit for your research. If you are not sure, read the appropriate sections before making your selection.

[x] Life sciences [ ] Behavioural & social sciences [ ] Ecological, evolutionary & environmental sciences

For a reference copy of the document with all sections, see nature.com/documents/nr-reporting-summary-flat.pdf

Life sciences study design

All studies must disclose on these points even when the disclosure is negative.

Sample size	No statistical methods were used to predetermine sample size. Sample sizes were chosen based on previous experience with each assay and on standards in the field for primary human neutrophil studies. Lipidomics: N = 3 donors per condition (three technical replicates per donor for the PMA time course, two for the PLD1 inhibitor experiment). Biochemical and lysate-based assays: N = 4–5 independent donors or experiments per condition. ROS assays in $CD34^{+}$-derived neutrophil-like cells: N = 7 donors. Single-cell imaging: N = 2 donors, with the exact number of cells analysed per donor per condition given in each figure legend.
Data exclusions	No data were excluded, except in the single-cell imaging pipeline, where cells touching the image border or with a cross-sectional area outside 20–2000 μm^2 were excluded by pre-established criteria.
Replication	All experiments were replicated across independent donors, with the number of donors given in each figure legend (N = 2 for single-cell imaging, N = 3 for lipidomics, N = 4–5 for biochemical and lysate-based assays and N = 7 for ROS in $CD34^{+}$-derived neutrophil-like cells). All replication attempts were successful.
Randomization	Not relevant: experiments were performed on cells from individual donors under defined in-vitro conditions with no allocation into groups, so randomisation does not apply.
Blinding	Blinding was not performed; samples were anonymised donor material processed under identical conditions, and quantification was automated (scripted pipelines), limiting operator bias.

Reporting for specific materials, systems and methods

We require information from authors about some types of materials, experimental systems and methods used in many studies. Here, indicate whether each material, system or method listed is relevant to your study. If you are not sure if a list item applies to your research, read the appropriate section before selecting a response.

Materials & experimental systems

n/a	Involved in the study
☐	☒ Antibodies
☒	☐ Eukaryotic cell lines
☒	☐ Palaeontology and archaeology
☒	☐ Animals and other organisms
☒	☐ Clinical data
☒	☐ Dual use research of concern
☒	☐ Plants

Methods

n/a	Involved in the study
☒	☐ ChIP-seq
☐	☒ Flow cytometry
☒	☐ MRI-based neuroimaging

Antibodies

Antibodies used	Primary antibodies: anti-PtdIns(4)P IgM (Echelon Biosciences, Z-P004, clone PI4-2, lot XCM091624-21, 1:250); anti-PtdIns(4,5)P_2 IgM (ThermoFisher Scientific, MA3-500, clone 2C11, lot AB399605, 1:250); anti-PtdIns(3,4,5)P_3 IgM (Echelon Biosciences, Z-P345, clone RC6F8, lot AB102821-23, 1:250); anti-PLD1 (Abcam, ab235997, clone EP1506Y, lot 1056216-18, 1:400); anti-PIP5K1A (ThermoFisher Scientific, MA5-47257, clone HL2011, lot AA46625051, 1:200 for immunofluorescence and 1:1000 for dot blots); anti-PIP5K1C (ThermoFisher Scientific, 702444, clone 16H4L5, lot 2554946, 1:200 for immunofluorescence and 1:1000 for dot blots); anti-NADK (Cell Signaling Technology, 55948, rabbit polyclonal, lot 1, 1:1000); anti-cleaved histone H3 (clone 3D9, in-house, 1:200).<br>Secondary antibodies: F(ab')$_2$-goat anti-rabbit IgG (H+L) cross-adsorbed Alexa Fluor 488 (ThermoFisher Scientific, A-11070, lot 2563831, 1:1000); goat anti-mouse IgG (H+L) cross-adsorbed Alexa Fluor 647 (ThermoFisher Scientific, A-21235, lot 3192624, 1:1000); goat anti-mouse IgG (H+L) highly cross-adsorbed Alexa Fluor 488 (ThermoFisher Scientific, A-11029, lot 1789729, 1:1000); Peroxidase AffiniPure goat anti-rabbit IgG, F(ab')$_2$ fragment specific (Jackson ImmunoResearch, 111-035-047, lot 000000135784, 1:1000).
Validation	All commercial antibodies have been validated and quality tested by the manufacturer. Antibody details can be found:<br>PI4P: https://www.echelon-inc.com/product/purified-anti-ptdins4p-igm/<br>PIP2: https://www.thermofisher.com/antibody/product/Phosphatidylinositol-4-5-bisphosphate-Antibody-clone-2C11-Monoclonal/MA3-500<br>PIP3: https://www.echelon-inc.com/product/purified-anti-ptdins345p3-igm/<br>PLD1: https://www.abcam.com/en-us/products/primary-antibodies/phospholipase-d1-antibody-ep1506y-bsa-and-azide-free-ab235997<br>PIP5K1A: https://www.thermofisher.com/antibody/product/PIP5K1A-Antibody-clone-HL2011-Recombinant-Monoclonal/MA5-47257?imageId=987465<br>PIP5K1C: https://www.thermofisher.com/antibody/product/PIP5K1C-Antibody-clone-16H4L5-Recombinant-Monoclonal/702444?imageId=132414<br>3D9 antibody validated in: Dorothea Ogmore Tilley et al. Histone H3 clipping is a novel signature of human neutrophil extracellular traps eLife 11:e68283 (2022)<br>NADK: https://www.cellsignal.com/products/primary-antibodies/nadk-antibody/55948<br>Goat anti-rabbit AF488 (A-11070): https://www.thermofisher.com/antibody/product/Goat-anti-Rabbit-IgG-H-L-Cross-Adsorbed-Secondary-Antibody-Polyclonal/A-11070<br>Goat anti-mouse AF647 (A-21235): https://www.thermofisher.com/antibody/product/Goat-anti-Mouse-IgG-H-L-Cross-Adsorbed-Secondary-Antibody-Polyclonal/A-21235<br>Goat anti-mouse AF488 (A-11029): https://www.thermofisher.com/antibody/product/Goat-anti-Mouse-IgG-H-L-Highly-Cross-Adsorbed-Secondary-Antibody-Polyclonal/A-11029<br>Peroxidase goat anti-rabbit F(ab')2 (111-035-047): https://www.jacksonimmuno.com/catalog/products/111-035-047

Plants

Seed stocks	N/A
Novel plant genotypes	N/A
Authentication	N/A

Flow Cytometry

Plots

Confirm that:

- [x] The axis labels state the marker and fluorochrome used (e.g. CD4-FITC).
- [x] The axis scales are clearly visible. Include numbers along axes only for bottom left plot of group (a 'group' is an analysis of identical markers).
- [x] All plots are contour plots with outliers or pseudocolor plots.
- [x] A numerical value for number of cells or percentage (with statistics) is provided.

Methodology

Sample preparation	Neutrophils were the purified input population; the AF-488+ (phagocytic) fraction is reported per condition (Supplementary Fig. 6E), with the gating strategy shown in Supplementary Fig. 9.
Instrument	CytoFlex flow cytometer (Beckman Coulter)
Software	CytExpert (version 2.3, Beckman Coulter) for acquisition on the CytoFlex; FlowJo (version 11.1.1, BD Biosciences) for analysis.
Cell population abundance	Neutrophils were the purified input population; the AF-488$^+$ (phagocytic) fraction is reported per condition (Supplementary Fig. 6E). The gating strategy is shown in Supplementary Fig. 9.
Gating strategy	Cells were gated on FSC/SSC to identify neutrophils and exclude debris; the AF-488+ gate was set on unstimulated cells. A representative gating strategy is shown in Supplementary Fig. 9.

- [x] Tick this box to confirm that a figure exemplifying the gating strategy is provided in the Supplementary Information.
