# Supplementary material for: Phospholipase D1 activates NAD kinase fuelling neutrophil extracellular trap formation
Source: Nat Commun. 2026 Nov 6;17:11497. doi: 10.1038/s41467-026-79319-6 (PMC13743754; doi:10.1038/s41467-026-79319-6)
Supplement: Supplementary file 4 [file 41467_2026_79319_MOESM9_ESM.pdf]

nature portfolio

Peer Review File

Phospholipase D1 activates NAD kinase fuelling Neutrophil Extracellular Trap formation.

Corresponding Author: Dr Dhiren Patel

This file contains all reviewer reports in order by version, followed by all author rebuttals in order by version.

Version 0:

Reviewer comments:

Reviewer #1

(Remarks to the Author)
The authors used integrated lipidomic, biochemical and pharmacological analyses to characterize the molecular mechanisms underlying phospholipase D1 (PLD1) regulation of NET formation in human neutrophils. They identify the PLD1-phosphatidic acid/PI(4,5)P2-NAD kinase signaling pathway that generate NADP to sustain glucose metabolism and NOX2-dependent ROS production. Pharmacological blockade or genetic disruption of PLD1 suppressed NADK activity and NET extrusion. These findings extend previous reports on impaired NET release in PLD1-deficient mice, and define how PLD1-driven membrane lipid remodeling couples NADK activity to NET release. I have the following comments for the authors` consideration.

Specific Comments.
1. The authors did not distinguish suicidal and vital NETosis. Indeed, the text appears to focus on NOX2-dependent NETosis, whereas the studies with C. albicans and Ca2+ ionophore also addressed vital NETosis. Since the mechanisms underlying various forms of NETosis and their biological function differ, it would be essential to better define the role of PLD1 in these processes. Would PLD1 also affect mitochondrial vital NETosis?
2. PMA was used to trigger suicidal NETosis. It would be important to document that biologically more relevant stimuli, such DAMPs or PAMPs, could also induce generation of PA and PI(4,5)P2 sufficient to evoke NET release.
3. E. coli triggered PLD activity, and pharmacological blockade of PLD or PLD1 reduced ROS production during E. coli phagocytosis. What was the rationale for studying phagocytosis (neither mentioned in the Introduction or Discussion)? Did E. coli evoke NET release? Could the PLD1-NADK coupling function as switch from phagocytosis to NETosis?
4. What is the link between reducing extracellular acidification rate and NET release by PLD inhibitors?
5. How could NADK activation through phosphorylation by PKC or Akt and direct binding of PA/PI(4,5)P2 be dissected in PMA-stimulated neutrophils?
6. The results imply a role for NADK in ROS generation and NET formation (which are interlinked), but do not establish dynamic tuning of NADP production during neutrophil activation in general.
7. Depicting duplicates as individual data points is unusual and may be misleading in terms of n values. Figure legend should explain what the different symbols mean.

Minor Points.
1. Lines 394-395. “selectively” appears twice in the same sentence.
2. Fig. 4G. Please revise “representative blot from 1 independent experiment from one donor”.
3. Suppl. Figure 1F and G. labeling of Y-axis may be misleading for using “PMA/vehicle” as the reference when NET release was triggered with A23187 or C. albicans hyphae.

Reviewer #2

(Remarks to the Author)
In the manuscript Phospholipase D1 activates NAD kinase fuelling NET formation, the authors propose a mechanism whereby neutrophil activation results in PLD1 mediated production of PA and PI(4,5)P2 which activate NADK, increasing NADP availability for NADPH oxidase activity required for NET formation. They employ lipidomics together with biochemical

and functional assays in the presence of chemical inhibitors to delineate this pathway. Inhibitors are used at concentrations pre-defined in the literature. Conceptually, this provides additional granularity to the existing literature regarding potential mechanisms of control. I have a number of major points I would like to raise

1. What is the evidence that access to PI subspecies is rate limiting. In figure 2 A-D, whilst PMA increase PI production, all are present to varying degrees in the unstimulated and PLD1i treated neutrophils
2. The IF data included in figure 2E Is not convincing – I can't see evidence of PIP3 expression and effects of PLD1i and PKC glo on PI4P look modest – no quantification and single image representation
3. I have major concerns re how the data is displayed and analysed. The majority of expts n=2 with technical replicates (all shown as individual data points in the graphs). One-way Anova has been used for statistical analysis, which is not appropriate given the n number.
4. How do the authors explain the persistent elevation in OCR in the presence of PKC glo (Fig 3 C)
5. Figure 4G n=1. No control data / molecular markers included with this image- similarly for figure 5A
6. In the abstract the authors reference NADP sustaining glucose metabolism but provide no direct evidence to support this statement.

Overall, the limited n number make it very difficult to have confidence in the data interpretation, I do not understand how the authors have been able to perform statistical analysis in this context.

Minor point

Figure legend 2 – the authors state Pi4P when they mean PIP3

Insufficient detail provided re use of CD34+ stem cells and PLD1 CRISPR knockdown

Reviewer #3

(Remarks to the Author)
Summary
This manuscript examines how PLD1 regulates NET formation by linking phospholipid signaling to NADK-dependent NADP production and downstream NOX2 activity. The overall question is interesting, and the study has a number of strengths, particularly the combination of genetic perturbation, lipid imaging, and metabolite measurements. The central model is also plausible. My main concerns are with how some of the data are interpreted and presented. In several places, the manuscript overstates what the inhibitor and metabolic data show. There are also a number of internal inconsistencies in the figure citations and some claims from microscopy that would benefit from quantification.

Specific Comments
1. The manuscript repeatedly conflates glucose metabolism, glycolysis, and PPP activity.
This is the biggest issue in the paper. These terms are used too loosely and at times interchangeably, especially in the NADK/G6PD/ECAR sections. For example, the manuscript states that conversion of NAD to NADP increases NADP availability "to sustain glucose metabolism and NOX2-dependent ROS production," and later argues that PLD inhibition attenuates "glucose metabolism" based on ECAR. That is too broad. NADK is directly relevant to the NADP(H) pool, oxidative PPP flux, and NOX2 function. That is not the same as saying it sustains glycolysis or "glucose metabolism" more generally. Likewise, reduced ECAR after G6PDi or PLD inhibition does not by itself show direct inhibition of glycolysis. In activated neutrophils, this could also reflect disruption of PPP cycling and a broader reduction in activation-associated glucose flux. The authors need to clean this up throughout the manuscript.

2. Some inhibitor-based conclusions are stronger than the data support.
There are several places where the text makes exclusionary pathway statements that do not seem fully supported by the experiments shown. For example, the statement that PIP2 induction depends on PLD1 and a calcium-independent PKC isoform rather than ROS, glucose metabolism, or Akt signaling is confusing as written. In that section, the inhibitor set appears to address ROS/flavoprotein activity, Akt, and PKC isoforms, but not glucose metabolism directly. Similarly, the claim that PMA-induced PLD activity is independent of ROS seems stronger than warranted based on the apparent magnitude of the DPI effect. These statements should either be toned down or supported more clearly.

3. There are multiple internal inconsistencies in figure citation and figure/model alignment.
The paper needs a careful pass here. The inhibitors shown in the model figure should match what is actually tested in the supporting data. I also do not see DPI used in the figure cited to support the claim around line 164. Later, line 322 appears to call out Fig. 4G, but the relevant data look to be in Fig. 4E–F. These are not major issues individually, but there are enough of them that they become distracting.

4. The microscopy-based claims need more quantification.
There are multiple points where representative images are used to support changes in localization or abundance without quantitative analysis. This is particularly relevant where the manuscript discusses PLD1 redistribution and relocalization of lipid species or associated proteins. If the conclusion depends on altered localization or enrichment, that should be quantified.

5. The NADK phosphorylation measurements are not described clearly enough.
I was confused by how NADK phosphorylation was specifically assessed. As written, the phos-tag SDS-PAGE description is not clear enough to understand how this was quantified for NADK specifically rather than phosphoproteins more broadly. If the authors ran a phos-tag gel and then immunoblotted for NADK, that should be stated explicitly in the Methods and figure legend. If that is not what was done, then the wording around this result needs to be revised.

6. The ECAR data should be interpreted more narrowly.
Related to point 1, I do not think the ECAR data support the broad conclusion that PLD inhibition attenuates “glucose metabolism.” At most, these data show that PLD1 inhibition, like G6PD inhibition, reduces activation-associated extracellular acidification. That is compatible with impaired PPP-linked glucose flux and/or PPP cycling, but it does not establish direct inhibition of glycolysis. The text should be revised accordingly.

7. Some of the supplemental presentation feels redundant.
I was not sure what Supplementary Fig. 7A–C adds beyond Fig. 4A–C other than the inclusion of additional time points. If the purpose is simply to show temporal consistency, that should be stated more clearly. As presented, it feels repetitive.

Version 1:

Reviewer comments:

Reviewer #1

(Remarks to the Author)
I appreciate the authors` thoughtful responses to my previous comments. The new data, analyses and extensive rewriting have strengthened this interesting manuscript. I have no further comments.

Reviewer #2

(Remarks to the Author)
The authors have undertaken a significant program of work to expand the experimental repeats increasing confidence in the reported results. They have also provided text changes to ensure that they do not over interpret the results provided and removed data sets where for logistic reasons further experimental repeats were not feasible. Relevant methodology text has been provided/expanded as requested. I am happy these revisions have addressed the concerns raised at first review.

Reviewer #3

(Remarks to the Author)
The authors have carefully addressed my previous concerns. The revised manuscript uses more precise metabolic terminology, appropriately narrows the interpretation of the ECAR and inhibitor experiments, corrects the figure inconsistencies, and provides additional quantitative support for the microscopy-based conclusions. The removal of the unclear NADK phosphorylation experiment and clarification of the supplemental figures also improve the manuscript.

I have one remaining minor correction: the Supplementary Figure 6 legend states that glycolysis and the PPP both convert $NADP^+$ to NADPH. Glycolysis does not directly perform this conversion; $NADP^+$ is reduced to NADPH through the oxidative PPP.

Open Access This Peer Review File is licensed under a Creative Commons Attribution 4.0 International License, which permits use, sharing, adaptation, distribution and reproduction in any medium or format, as long as you give appropriate credit to the original author(s) and the source, provide a link to the Creative Commons license, and indicate if changes were made.
In cases where reviewers are anonymous, credit should be given to 'Anonymous Referee' and the source.
The images or other third party material in this Peer Review File are included in the article's Creative Commons license, unless indicated otherwise in a credit line to the material. If material is not included in the article's Creative Commons license and your intended use is not permitted by statutory regulation or exceeds the permitted use, you will need to obtain permission directly from the copyright holder.
To view a copy of this license, visit https://creativecommons.org/licenses/by/4.0/

Point-by-Point Response to Reviewers

Phospholipase D1 activates NAD kinase fuelling Neutrophil Extracellular Trap formation

In response to the reviewers' concerns, we include new experiments and analyses in this new version of the manuscript.

In this point-by-point response, the reviewers' comments are reproduced in italics, followed by our response. All changes in the manuscript are highlighted using track changes.

Response to Reviewer 1

Reviewer comment. *The authors used integrated lipidomic, biochemical and pharmacological analyses to characterize the molecular mechanisms underlying phospholipase D1 (PLD1) regulation of NET formation in human neutrophils. They identify the PLD1–phosphatidic acid/PI(4,5)P_2–NAD kinase signaling pathway that generate NADP to sustain glucose metabolism and NOX2-dependent ROS production. Pharmacological blockade or genetic disruption of PLD1 suppressed NADK activity and NET extrusion. These findings extend previous reports on impaired NET release in PLD1-deficient mice, and define how PLD1-driven membrane lipid remodeling couples NADK activity to NET release. I have the following comments for the authors' consideration.*

We thank the reviewer for their positive evaluation and for recognising the value of defining the molecular mechanism coupling membrane lipid remodeling to NADK activity. We address each comment below.

Specific Comments

Point 1. *The authors did not distinguish suicidal and vital NETosis. Indeed, the text appears to focus on NOX2-dependent NETosis, whereas the studies with C. albicans and Ca^{2+} ionophore also addressed vital NETosis. Since the mechanisms underlying various forms of NETosis and their biological function differ, it would be essential to better define the role of PLD1 in these processes. Would PLD1 also affect mitochondrial vital NETosis?*

We thank the reviewer for raising this distinction. Our extracellular-DNA and imaging assays measure NET release rather than cell fate, so they do not formally distinguish suicidal (lytic) NET release from vital release, in which the cell remains viable. What our data establish is that all three stimuli we tested were NOX2-dependent and DPI-sensitive. Our study examines PLD1 across three NET-inducing stimuli with distinct upstream pathways that converge on NOX2: PMA (direct PKC engagement), calcium ionophore A23187 (Ca^{2+}-driven), and *Candida albicans* hyphae (physiological, receptor-mediated). Pharmacological PLD1 inhibition and CRISPR-mediated PLD1 knockout reduced NET release under all three conditions, in parallel with reductions in NAD kinase (NADK) activity, the NADP(H) pool, and NOX2-derived reactive oxygen species (ROS). The shared effect across mechanistically distinct triggers points to a common upstream requirement: PLD1-driven NADP(H) provision to NOX2.

Our data localise PLD1 to ROS production itself. In Figure 3E, we used two stimuli that bypass NOX2: hydrogen peroxide (H_2O_2), which supplies ROS directly, and nigericin, which drives ROS-independent NETosis. Neither was blocked by PLD or PLD1 inhibition, whereas NET release

under NOX2-dependent stimuli was. PLD1 is required to generate the ROS that drives NETosis but is dispensable if ROS are supplied exogenously or in ROS-independent NET formation. This places PLD1 upstream of ROS, on the NOX2-dependent arm, rather than in ROS-independent NET formation such as the nigericin response. Consistently NET release induced by the three NOX2-dependent stimuli was sensitive to the NOX2 inhibitor DPI.

We agree with the reviewer that the role of PLD1 in mitochondrial vital NETosis is interesting. However, our present model concerns cytosolic NADK and NADP(H) pool that supplies NOX2. Whether the PLD1–NADK axis contributes to mitochondrial-ROS-driven vital NETosis is an interesting question, and it is one we would be keen to pursue in future work; the distinct subcellular NADP(H) pools involved make it a particularly attractive avenue. We added an explicit statement to this effect in the Discussion.

Manuscript text changes:

Results, lines 84–97: PLD converts phosphatidylcholine (PC) to PA by replacing the choline headgroup with a monophosphate[1,2] (Figure 1G). Of the six mammalian PLD isoforms, only PLD1 and PLD2 synthesise PA (Figure 1G). Targeting each isoform with two inhibitors (Figure 1G) for PLD1 (VU0359595, hereafter called PLD1i and VU0155069) and PLD2 (VU0364739 and VU0155056), revealed that PLD1, but not PLD2, is required for NET formation induced by PMA, a direct protein kinase C (PKC) activator (Figure 1H). PLD1 inhibition also blocked NET formation triggered by two distinct NOX2-dependent stimuli, the calcium ionophore A23187 and heat-killed *Candida albicans* hyphae (a physiological, receptor-mediated stimulus)[3] (Supplementary Figure 1F–G). We confirmed the inhibitor results with live microscopy (Supplementary Movies 1–5) and with immunofluorescent staining (IF) (Supplementary Figure 2A–C) of neutrophils activated for 4 hours with PMA. We detected NETs using the NET-specific antibody 3D9, which recognises cleaved histone H3[4], alongside a panel of control inhibitors, Neutrophil Elastase inhibitor (NEi, BAY 85-8501), DPI and the pan-isoform PKC inhibitor, Gö6983 (PKCglo)—to validate the pathway specificity (Supplementary Figure 2).

Discussion, lines 354–358: Consistent with a requirement for ROS production rather than for post-ROS NET-execution events, PLD1 inhibition did not impair NET formation driven by exogenous H_2O_2 (which bypasses NOX2) or by nigericin (a ROS-independent stimulus). Whether the PLD1–NADK axis additionally contributes to mitochondrial-ROS-driven vital NETosis was not tested here and remains an open question.

Point 2. *PMA was used to trigger suicidal NETosis. It would be important to document that biologically more relevant stimuli, such as DAMPs or PAMPs, could also induce generation of PA and PI(4,5)P_2 sufficient to evoke NET release.*

We thank the reviewer for this important point. In the new version of the manuscript, we report per-cell PI(4,5)P_2 intensity by quantitative immunofluorescence after stimulation with three pathogen-associated molecular patterns (PAMPs) and damage-associated molecular patterns (DAMPs): bacterial LPS, the chemotactic peptide fMLP, and heme priming before TNF-α stimulation. These three activators increased PI(4,5)P_2 intensity above the unstimulated level, although to a lesser extent than PMA (new Supplementary Figure 4E–F). These data show that PI(4,5)P_2 production is induced not only by PMA but also in response to ligands that neutrophils encounter during infection and sterile inflammation. We did not measure phosphatidic acid (PA) directly under these conditions. We interpret the induced rise in PI(4,5)P_2 as a PA-dependent product of PIP5K activity, which serves as a readout of PLD1-pathway engagement. We have updated the Discussion to note that we did not directly quantify PA or assess PLD1 activity with these stimuli, which remains an open question for future studies.

Manuscript text change.

Results, lines 155–160: To test whether the PLD1–PIP_2 axis operates under other stimuli, we examined three damage- and pathogen-associated molecular patterns (DAMPs and PAMPs): bacterial lipopolysaccharide (LPS), the chemotactic peptide N-formyl-methionyl-leucyl-phenylalanine (fMLP), and heme applied after a 30-min tumour necrosis factor-α (TNFα) priming step. These stimuli increased PIP_2 intensity, although to a lesser extent than PMA, as measured by quantitative immunofluorescence (Supplementary Figure 4E–F).

Discussion, lines 385–386: Although we observed that DAMPs and PAMPs induced PIP_2 production, further studies will be required to confirm that this occurs via PA and PLD1 activity.

Point 3. *E. coli triggered PLD activity, and pharmacological blockade of PLD or PLD1 reduced ROS production during E. coli phagocytosis. What was the rationale for studying phagocytosis (neither mentioned in the Introduction or Discussion)? Did E. coli evoke NET release? Could the PLD1–NADK coupling function as switch from phagocytosis to NETosis?*

We thank the reviewer for raising this point. The Results now state our rationale explicitly. We included *E. coli* phagocytosis because, during this process, NOX2 produces ROS, and showed that PLD/PLD1 inhibition also attenuates ROS in this setting, alongside the NET-inducing stimuli. This indicates that PLD1 acts broadly on NOX2 rather than on a single effector programme. The key observation is a dissociation: PLD1 inhibition reduced the *E. coli*-triggered ROS burst while leaving *E. coli* ingestion unchanged (Supplementary Figure 6D vs 6E). This places the effect of PLD1 on the NOX2 oxidative burst rather than on uptake.

At the multiplicity of infection (MOI) of 10, used here, *E. coli* robustly induced phagocytosis but not NET release. We now describe these data as a phagocytosis paradigm in which PLD1 inhibition lowers NOX2-dependent ROS without blocking ingestion (Supplementary Figure 6D–F). Whether PLD1–NADK coupling sets a threshold between phagocytosis and NETosis at higher bacterial loads remains an open and interesting question.

Manuscript text change.

Results, lines 196–203: During phagocytosis, NOX2 is activated, prompting us to ask whether PLD1 is required for the ROS production in this context as well. We incubated neutrophils with opsonised *Escherichia coli* at a multiplicity of infection (MOI) of 10, a dose routinely used to assess phagocytosis. PLDi and PLD1i reduced ROS during *E. coli* phagocytosis (Supplementary Figure 6D) without affecting ingestion (Supplementary Figure 6E). PLD activity triggered by the phagocytic stimuli E. coli and zymosan was blocked by the actin inhibitor Cytochalasin D (Supplementary Figure 6F) and PLD inhibitors. These data indicate that PLD1 is required to activate NOX2 in different neutrophil effector functions.

Point 4. *What is the link between reducing extracellular acidification rate and NET release by PLD inhibitors?*

We thank the reviewer for this important question. The reduction in extracellular acidification rate (ECAR) and the loss of NET release are not causally linked to each other. Both are downstream consequences of a single upstream block: when PLD1 is inhibited, NADK can no longer expand the cellular NADP(H) pool.

We interpret our nicotinamide data through two established controls, DPI (which blocks NOX2) and G6PDi (which blocks the oxidative Pentose Phosphate Pathway, PPP). Both abolish ROS production and NETosis, confirming that NET release depends on an intact NADP(H)-to-NADPH-to-NOX2 supply line. PLD inhibition produces the same functional outcome but acts furthest upstream. Rather than blocking NADPH regeneration or NOX2 itself, it blocks NADK, so $NADP^+$ is never made and the whole pool contracts at its source. The nicotinamide measurements show this directly: under PLD inhibition NAD^+ stays high and the $NADP^+$ rise is lost, a signature reproduced by neither control (Figure 4A–C; Supplementary Figure 7A–C).

This shared bottleneck explains both readouts. A smaller NADP(H) pool leaves NOX2 short of the NADPH it needs, reducing ROS and NET release. It also lowers activation-associated acidification, measured as reduced ECAR. PLD and PLD1 inhibition reduced ECAR to the same extent as G6PDi (Supplementary Figure 6I–J). We interpret this as reduced activation-associated glucose handling downstream of the contracted NADP(H) pool.

This model is consistent with prior work on neutrophil NADPH metabolism. Britt et al. 2022[5] showed that the oxidative PPP powers the neutrophil burst and is required for ROS, NET release and pathogen killing. Amara et al. 2021[6] measured the nicotinamide pools directly and found that limiting glucose flux to the PPP contracts the same NADP(H) pool. Both position NADK-derived $NADP^+$ as the supply for the PPP-generated NADPH that NOX2 draws on during the burst.

Manuscript text change.

Results, lines 245–254: We next examined how PLD1 inhibition affects extracellular acidification rate (ECAR). As expected, G6PDi reduced the ECAR response to PMA or A23187, as did PLD and PLD1 inhibition (Supplementary Figure 6I–J). We interpret this as a reduction in activation-associated extracellular acidification, compatible with impaired PPP cycling or a broader reduction in activation-associated glucose flux; ECAR alone does not establish direct inhibition of glycolysis. Critically, the $NAD^+/NADP^+/NADPH$ patterns under PLD inhibition differed markedly from those induced by G6PDi or DPI (Figure 4A–C; Supplementary Figure 7A–C), placing PLD1 upstream of NADK-mediated $NADP^+$ synthesis rather than within the PPP or at NOX2. By reducing NADP(H) supply to NOX2, PLD1 inhibition therefore accounts for both the fall in ECAR and the block in NET release.

Discussion, lines 328–333: Activated neutrophils engage the PPP to power the NADPH-dependent oxidative burst[5,6], and pharmacological diversion of glycolytic flux away from the PPP depletes cellular NADPH and suppresses the NOX2-dependent burst[6]. Our direct nicotinamide-nucleotide measurements place PLD1, via NADK, upstream of this PPP-dependent step: by setting the size of the $NADP^+$ pool, PLD1 governs the NADP(H) supply on which PPP-coupled NADPH regeneration and, ultimately, NOX2-dependent NET release depend.

Point 5. *How could NADK activation through phosphorylation by PKC or Akt and direct binding of PA/PI(4,5)P_2 be dissected in PMA-stimulated neutrophils?*

We thank the reviewer for this thoughtful question. We distinguished PKC/Akt-dependent phosphorylation from direct lipid binding by measuring NADK activity across three stimuli and in a cell-free system, since phosphorylation of NADK by PKC and Akt has been reported to activate the enzyme in other cell types[7,8].

- In PMA-activated neutrophils, PKCglo was the only kinase inhibitor that reduced NADK activity, to a similar extent as PLDi and PLD1i (Figure 4D).

- In neutrophils activated with A23187 or *C. albicans* hyphae, PLDi and PLD1i, but not DPI, G6PDi, PKCglo, Akti nor Rafi, diminished NADK activity (Figure 4E–F). PKC is therefore required only under PMA, whereas PLD1 is required under all three stimuli.
- In a cell-free assay, PA and $PI(4,5)P_2$ activate recombinant NADK (Figure 5), while PLDi and PLD1i do not inhibit the enzyme (Supplementary Figure 7E). Lipid binding therefore activates NADK without any kinase signalling.

Mapping the precise lipid-binding residues on NADK will require a heterologous expression system and is a priority for future work, which we now note in the Discussion.

Manuscript text change.

Discussion, lines 319–327: A central finding of this report is that PLD1 regulates neutrophil $NADP^+/NAD^+$ metabolism through direct lipid-dependent activation of NADK. PLD1 inhibition consistently reduced $NADP^+$ and increased NAD^+ during PMA stimulation, indicating impaired NAD^+ phosphorylation and placing NADK downstream of PLD1. Notably, this $NADP^+/NAD^+$ shift reflected impaired NADK activity rather than altered oxidative-PPP flux or NOX2 activity. Although PA was reported to interact with subunits of the NOX2 complex[9], PLD1 inhibition did not reproduce the metabolic or ROS phenotypes observed with direct NOX2 inhibition. Instead, we found that PLD1 regulates cellular NADK activity across multiple stimuli, establishing NADK as the downstream metabolic effector linking PLD1-derived lipids to $NADP^+$ production.

Discussion, lines 343–345: Direct dissection of lipid binding to NADK, for example via point mutations in candidate PA/PIP_2-binding residues, will require structural identification of those residues and a heterologous expression system, and is a priority for future work.

Point 6. *The results imply a role for NADK in ROS generation and NET formation (which are interlinked), but do not establish dynamic tuning of NADP production during neutrophil activation in general.*

We thank the reviewer for this comment. The manuscript quantifies NAD^+, $NADP^+$ and NADPH at 10, 30 and 60 min. In PMA-stimulated cells, the $NADP^+$ pool increases progressively, whereas it remains flat in unstimulated cells. This indicates that $NADP^+$ production is dynamically tuned. Importantly, both NOX2 and PLD1 inhibition blunt the PMA-induced $NADP^+$ increase, but only DPI triggers an NADPH increase, because NADPH is then no longer consumed by NOX2. Across all timepoints, PLD and PLD1 inhibition also blocked the PMA-induced reduction in NAD^+, an effect not observed with DPI. This places PLD1 upstream, at NADK-mediated $NADP^+$ synthesis itself, and reproduces the phenotype we originally reported: PLD and PLD1 inhibition reduced $NADP^+$ without increasing NADPH.

We revised the Results and figure legends to present this $NADP^+$-centred comparison explicitly. The Discussion now states that NADK activity is dynamically tuned in a PLD1-dependent manner rather than constitutive.

Manuscript text change.

Results, lines 227–234: $NADP^+$ rose after stimulation and remained elevated throughout the time course, while it stayed unchanged in unstimulated cells, indicating that $NADP^+$ production is dynamically tuned during activation. To interpret the NAD^+, $NADP^+$ and NADPH measurements, we first established how DPI and G6PDi affect them in PMA-stimulated neutrophils. NOX2 consumes NADPH to generate ROS and $NADP^+$; blocking this step with DPI caused NADPH to

accumulate and $NADP^+$ to fall. The oxidative PPP regenerates NADPH from $NADP^+$; blocking this step with G6PDi caused $NADP^+$ to accumulate (Figure 4A–C, Supplementary Figure 7A–C), consistent with prior work linking oxidative PPP flux to NADPH supply for the NOX2 burst[5,6].

Point 7. *Depicting duplicates as individual data points is unusual and may be misleading in terms of n values. Figure legend should explain what the different symbols mean.*

Response. We agree with the reviewer. In the new version of the manuscript, each plotted point represents the mean of two technical replicates of a single donor. Biochemical panels now include 4 or 5 independent donors per condition. Single-cell imaging panels remain at N = 2 donors, with the number of cells analysed per condition per donor given as a range in each figure legend. Figure legends now state what each symbol represents, the number of independent experiments, and the statistical test used. We added two additional donors to the two originally submitted, acquired independently on different days with freshly prepared reagents and inhibitors, providing independent batch replication. We used One-way ANOVA with Bonferroni post hoc correction throughout.

Manuscript text change.

Figure legends (donor- and experiment-based panels): Data are shown as mean ± s.e.m. Each point is one donor or independent experiment, plotted as the mean of 2 technical replicates and shown with a different symbol; the lipidomics PMA time course (Figure 1B–F) is plotted as the mean of 3 technical replicates. These panels have N = 4–5 independent donors or experiments per condition; the specific n is given in each legend. Statistical significance was determined by one-way ANOVA with Bonferroni's multiple comparisons test. *$P < 0.05$, **$P < 0.01$, ***$P < 0.001$, ****$P < 0.0001$.

Figure legends (single-cell fluorescence intensity panels): Data are shown as mean ± s.e.m. Each data point is representative of individual cells; N = 2 donors, with the number of cells or objects analysed per condition per donor specified as a range in each figure legend. Statistical significance was determined by one-way ANOVA with Bonferroni's multiple comparisons test.

Methods, lines 855–863: For each donor, or for each independent experiment in cell-free assays, the two technical replicates were averaged to give a single value per condition. For the lipidomics PMA time course (Figure 1B–F), each value is the mean of three technical replicates. For biochemical and lysate-based assays (N = 4–5 independent donors or experiments per condition), statistical significance was determined by one-way ANOVA with Bonferroni post hoc correction. For single-cell imaging panels (N = 2 donors, with the number of cells or objects analysed per condition per donor specified as a range in each figure legend), statistical significance was likewise determined by one-way ANOVA with Bonferroni's multiple comparisons test. Sample sizes and the test used are specified in each figure legend.

Minor Points

Point M1. *Lines 394–395. "selectively" appears twice in the same sentence.*

The sentence has been re-worded to remove the duplication.

Manuscript text change.

Discussion, lines 309–311: We found that the PIP5K1A isoform selectively bound anionic lipids, particularly PA and phosphoinositides and that PA enhanced PIP5K1A activity.

Point M2. *Fig. 4G. Please revise "representative blot from 1 independent experiment from one donor".*

We thank the reviewer for flagging this limitation.

The proprietary formulation of the SuperSep Phos-tag precast gels (FUJIFILM Wako) changed since the experiment reported in the first submission of this manuscript. With the available gels, we failed to obtain interpretable separation. Furthermore, delivery lag times for these gels increased to several months, making further optimisation difficult. We therefore decided to remove the Phos-tag panel and all statements derived from it across the manuscript.

This does not affect the manuscript's conclusions. We tested the mechanisms driving NADK phosphorylation in neutrophils, since it was reported that PKC and Akt phosphorylate NADK in other cell types[7,8]. We addressed the requirement of phosphorylation to activate NADK with enzymatic activity assays, where we tested inhibitors of PKC, Akt and Raf, as well as NOX2 and G6PD as controls, in neutrophils activated with three different stimuli:

- In PMA-activated neutrophils, PKCglo was the only kinase inhibitor that reduced NADK activity, to a similar extent as PLDi and PLD1i (Figure 4D).
- In neutrophils activated with A23187 or *C. albicans* hyphae, PLDi and PLD1i, but not DPI, G6PDi, PKCglo, Akti nor Rafi, diminished NADK activity (Figure 4E–F).

These results indicate that PLD1 is required for NADK activation, regardless of the stimulus, while PKC regulates NADK only in PMA-stimulated neutrophils. Our data indicate that NADK can be activated through two independent pathways: by classical phosphorylation, or by binding PA or PI(4,5)P_2. This is supported by the following observations. (1) In A23187- or *C. albicans*-activated neutrophils, PKC is not required for NADK activity, suggesting that either an unknown kinase phosphorylates NADK or an alternative mechanism operates. (2) PLDi and PLD1i do not inhibit recombinant NADK activity *in vitro* (Supplementary Figure 7E), excluding an off-target effect on the enzyme. (3) PA and PI(4,5)P_2 activate recombinant NADK in a cell-free assay (Figure 5). Together, these data indicate that PLD1-derived lipids directly activate NADK, a conclusion that does not depend on the removed panel.

Point M3. *Suppl. Figure 1F and G. Labelling of Y-axis may be misleading for using "PMA/vehicle" as the reference when NET release was triggered with A23187 or C. albicans hyphae.*

Response. We thank the reviewer; the Y-axis labels now read "% of stim. vehicle", since each panel is normalised to its own stimulated vehicle. The stimulus for each panel is given in the legend.

Response to Reviewer 2

Reviewer comment. *In the manuscript Phospholipase D1 activates NAD kinase fuelling NET formation, the authors propose a mechanism whereby neutrophil activation results in PLD1-mediated production of PA and PI(4,5)P_2 which activate NADK, increasing NADP availability for NADPH oxidase activity required for NET formation. They employ lipidomics together with biochemical and functional assays in the presence of chemical inhibitors to delineate this pathway.*

Inhibitors are used at concentrations pre-defined in the literature. Conceptually, this provides additional granularity to the existing literature regarding potential mechanisms of control. I have a number of major points I would like to raise.

We thank the reviewer for the careful reading and for recognising the additional mechanistic granularity our work provides. We address each point below.

Point 1. *What is the evidence that access to PI subspecies is rate limiting? In Figure 2A–D, whilst PMA increases PI production, all are present to varying degrees in the unstimulated and PLD1i-treated neutrophils.*

We thank the reviewer for this point. The reviewer is correct that phosphatidylinositol (PI) subspecies are present in unstimulated and PLD1i-treated cells and that the stimulus-evoked increase is incremental rather than absolute. We do not claim that PI subspecies are rate-limiting in absolute terms, and we have removed any wording that implied so.

Although phosphatidic acid (PA) and PI interconvert through the CDP-DAG/PI synthesis cycle, in which PLD-derived PA can feed into PI and downstream phosphoinositides[10], we have not measured this flux directly. Rigorously establishing flux from PLD1-derived PA into PI would require metabolic tracing experiments. We therefore do not claim that PLD1-derived PA gives rise to PI.

Our data support a more proximal mechanism: PLD1-derived PA, together with PLD1 itself, controls PIP5K1 to promote PI(4,5)P_2 synthesis. PIP5K1A is allosterically activated by PA[11,12], so PLD1-derived PA need not alter the bulk PI pool to raise PI(4,5)P_2. Instead, it tunes the PIP5K1A-catalysed conversion of pre-existing PI(4)P. Consistently, PLD1 inhibition reduced PIP5K1A activity and the stimulus-induced increase in PI(4,5)P_2 while leaving PI subspecies broadly detectable, exactly as the reviewer notes. The model schematic depicts PI(4,5)P_2 synthesis but draws the PA-to-PI step as a dashed line to mark this boundary. We revised the Results and Discussion and now state that the relative contributions of de novo PI synthesis versus turnover of pre-existing pools remain an open question.

Manuscript text change.

Results, lines 178–180: Mechanistically, PA acts as an allosteric activator of PIP5K1A[11,12]; accordingly, PLD1 inhibition reduced PIP5K1A activity in neutrophils and the stimulus-induced rise in PIP_2 in parallel, even though PI subspecies remained detectable.

Results, lines 131–134: Although PA and PI can interconvert through the cytidine diphosphate-diacylglycerol (CDP-DAG)/PI cycle[10], we did not measure this flux directly; to look for any relationship, we examined PI subspecies whose fatty acyl chains matched the four major PA species identified earlier (Figure 1C–F).

Discussion, lines 315–318: The relative contributions of de novo PI synthesis (via the CDP-DAG/PI cycle)[10] versus turnover of pre-existing pools remain an open question that the present data do not resolve. Our data instead support PLD1-derived PA acting as an allosteric input on PIP5K1A rather than as a determinant of bulk PI subspecies abundance.

Point 2. *The IF data included in Figure 2E is not convincing, I can't see evidence of* PIP_3 *expression and effects of PLD1i and PKC Gö on PI4P look modest, no quantification and single image representation.*

Response. We thank the reviewer for this point and have addressed each concern with quantification and orthogonal data.

Quantification: We now quantify per-cell mean fluorescence intensity for PI(4)P, PI(4,5)P_2 and PI(3,4,5)P_3 across 74–298 cells per donor per condition (N = 2 donors), replacing the single-image representation (Figure 2F–H). PI(4)P and PI(4,5)P_2 both accumulate on stimulation and are significantly reduced by PLD1i and PKCglo, confirming that the effects are real rather than modest.

Orthogonal confirmation: PI(4,5)P_2 is the functionally central readout here, as it is the phosphoinositide that directly binds and activates NADK (Figure 5). It is also the most rigorously supported: beyond the single-cell imaging, PI(4,5)P_2 is independently corroborated by a mass ELISA in two stimulation contexts, PMA (Figure 2I) and A23187 (Supplementary Figure 4C). This ELISA is a separate assay with its own replication (N = 5 for PMA and A23187), so the PI(4,5)P_2 conclusion does not rest on the imaging alone.

PI(3,4,5)P_3 gave a signal only just above background by immunofluorescence, consistent with its highly transient nature and rapid turnover. ELISA values likewise fell below the limit of detection in most conditions (≤0.05 pmol, far below the PI(4,5)P_2 signal on the same material). We therefore show only the per-cell PI(3,4,5)P_3 intensity in the manuscript, provide the ELISA here for transparency, and make no claims about PI(3,4,5)P_3.

The image acquisition and fully scripted analysis pipeline (Python and scikit-image; marker-controlled watershed, two-stage background subtraction, per-cell intensity quantification) are described in the Methods.

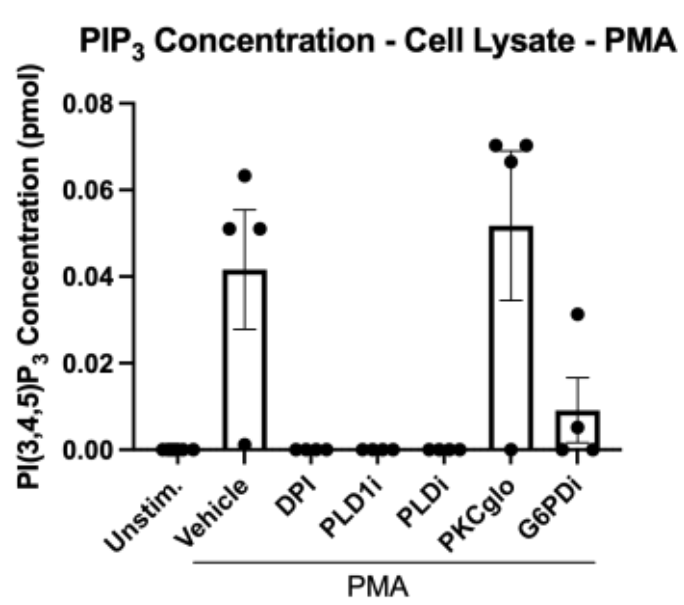

Legend: PIP_3 concentrations in lysates of neutrophils treated with the indicated inhibitors before 30 min PMA activation. Data shown as mean ± s.e.m., N = 4 donors. We plotted the mean of two technical replicates for each donor.

Manuscript text change.

Results, lines 139–143: PMA triggered PI4P and PIP_2 accumulation in the cytoplasm and nucleoplasm, which was abrogated upon PLD1 inhibition (Figure 2E). Quantification of the mean fluorescence intensity confirmed that PLD1i and PKCglo, but not DPI, reduced PI4P and PIP_2 (Figure 2F–G). PIP_3 gave a signal close to background by IF, consistent with its highly transient nature (Figure 2E,H).

Methods, lines 761–782: Images were acquired on Leica confocal microscopes and processed with LAS-X. PIP_2 panels were acquired with an HC PL APO 63×/1.32 oil-immersion objective at 2048×2048 pixels, whereas PI4P and PIP_3 panels were acquired at 1024×1024 pixels on an SP8 AOBS with a 100× oil objective. For each target, all conditions were imaged in the same run and analysed with a fully scripted pipeline in Python (scikit-image v0.26.0)[13]. Each channel was recorded across all Z planes, and the stack was combined by sum-intensity projection before

measurement. Background was removed in two stages: a per-slice rolling-ball estimate per channel, followed by a per-cell local correction subtracting the median intensity of a surrounding annular ("donut") ring, excluding neighbouring cells and image borders, with an image-wide non-cell median as a fall-back. Cells were segmented by a marker-controlled watershed[14]; the DAPI-stained nucleus of each cell served as the starting marker (Gaussian smoothing, Li thresholding and morphological closing, which merges the lobes of a single polymorphonuclear nucleus without joining adjacent cells), and the cell footprint was defined by Li (or, as a fall-back, triangle) thresholding of the combined PLD1-and-phosphoinositide signal. For each cell the nucleus was excluded, and intensities were measured for PLD1 and the phosphoinositide channel; we report the mean fluorescence intensity. For PLD1, the rim (0.45 µm wide, a plasma-membrane proxy) was additionally excluded and the mean cytoplasmic fluorescence intensity measured in the interior compartment. Cells touching the image border, or with a cross-sectional area outside 20–2000 μm^2, were excluded, and the number of cells retained per condition per donor is specified as a range in each figure legend (two independent donors). For each target, the pooled single-cell distributions were compared across conditions by one-way ANOVA with Bonferroni's multiple comparisons test.

Point 3. *I have major concerns re how the data is displayed and analysed. The majority of expts n=2 with technical replicates (all shown as individual data points in the graphs). One-way ANOVA has been used for statistical analysis, which is not appropriate given the n number.*

We thank the reviewer for this comment. The revised manuscript addresses the data display and statistical analysis as follows:

- Biochemical and lysate-based experiments are now N = 4–5 biological donors per condition, each plotted point being one donor (2–3 technical duplicates averaged). Specifically, the $PI(4,5)P_2$ ELISA is N = 5; the NAD^+/$NADP^+$/NADPH panels N = 4; NAD kinase (NADK) activity N = 5 (PMA) and N = 4 (A23187, *C. albicans*); luminol reactive oxygen species (ROS) N = 5 (PMA, A23187 and *C. albicans*) and N = 4 (*E. coli*); and Seahorse oxygen consumption rate (OCR)/extracellular acidification rate (ECAR) N = 5. For the N = 4 panels, the additional donors were acquired as a separate, independent batch (different days, freshly prepared reagents), providing the independent batch replication as requested.
- Technical replicates are no longer shown as independent points. Only biological replicates (or, for imaging, individual cells) are plotted, with each bar showing the biological replicates overlaid.
- Immunofluorescence panels remain at N = 2 donors, with the number of cells per condition per donor given as a range in each figure legend. In these panels each plotted point is one cell, and the comparison is supported by the large cell numbers and the matched $PI(4,5)P_2$ ELISA.
- We applied one-way ANOVA with Bonferroni post hoc correction throughout. This is now appropriate because the biochemical panels are powered at N = 4–5 biological replicates, addressing the concern that ANOVA was not justified at the lower n of the original submission.

The re-plotted datasets are shown in the revised manuscript figures.

Manuscript text change.

Figure legends (donor- and experiment-based panels): Data are shown as mean ± s.e.m. Each point is one donor or independent experiment, plotted as the mean of 2 technical replicates and shown with a different symbol; the lipidomics PMA time course (Figure 1B–F) is plotted as the mean of 3 technical replicates. These panels have N = 4–5 independent donors or experiments per

condition; the specific n is given in each legend. Statistical significance was determined by one-way ANOVA with Bonferroni's multiple comparisons test. *P < 0.05, **P < 0.01, ***P < 0.001, ****P < 0.0001.

Figure legends (single-cell fluorescence intensity panels): Data are shown as mean ± s.e.m. Each data point is representative of individual cells; N = 2 donors, with the number of cells or objects analysed per condition per donor specified as a range in each figure legend. Statistical significance was determined by one-way ANOVA with Bonferroni's multiple comparisons test.

Methods, lines 855–863: For each donor, or for each independent experiment in cell-free assays, the two technical replicates were averaged to give a single value per condition. For the lipidomics PMA time course (Figure 1B–F), each value is the mean of three technical replicates. For biochemical and lysate-based assays (N = 4–5 independent donors or experiments per condition), statistical significance was determined by one-way ANOVA with Bonferroni post hoc correction. For single-cell imaging panels (N = 2 donors, with the number of cells or objects analysed per condition per donor specified as a range in each figure legend), statistical significance was likewise determined by one-way ANOVA with Bonferroni's multiple comparisons test. Sample sizes and the test used are specified in each figure legend.

Point 4. *How do the authors explain the persistent elevation in OCR in the presence of PKC Gö (Fig. 3C)?*

We thank the reviewer for raising this point. The persistent OCR under PKCglo is specific to A23187 stimulation (now Figure 3D); upon PMA activation, PKCglo collapses the OCR response, as expected for a PKC-dependent NOX2 programme. This A23187 result mirrors the NADK data: PKCglo does not appreciably reduce NADK activation in the A23187 NADK activity panel (Figure 4E). We interpret this as follows. Under A23187, NADK is activated by PLD1-derived PA/PI(4,5)P_2 rather than by PKC-mediated phosphorylation. PKC inhibition therefore leaves NADK activity, and $NADP^+$ production, intact. Because the $NADP^+$ pool feeding the oxidative pentose phosphate pathway (PPP) is preserved, the PPP-coupled bioenergetic readouts are preserved: both OCR and activation-associated ECAR are maintained despite PKCglo treatment. In contrast, G6PD inhibition, which acts within the PPP itself, abrogates the activation-associated ECAR (Supplementary Figure 6I–J). The retained OCR and ECAR are thus the bioenergetic counterpart of preserved, phosphorylation-independent NADK activity. They reinforce our central conclusion that PKC phosphorylation is not the route to NADK activation under Ca^{2+}-driven stimulation.

Point 5. *Figure 4G n=1. No control data / molecular markers included with this image, similarly for Figure 5A.*

Response. We thank the reviewer for the suggestion.

The proprietary formulation of the SuperSep Phos-tag precast gels (FUJIFILM Wako) changed since the experiment reported in the first submission of this manuscript. With the available gels, we failed to obtain interpretable separation. Furthermore, delivery lag times for these gels increased to several months, making further optimisation difficult. We therefore decided to remove the Phos-tag panel and all statements derived from it across the manuscript.

This does not affect the manuscript’s conclusions. We tested the mechanisms driving NADK phosphorylation in neutrophils, since it was reported that PKC and Akt phosphorylate NADK in other cell types[7,8]. We addressed the requirement of phosphorylation to activate NADK with enzymatic activity assays, where we tested inhibitors of PKC, Akt and Raf, as well as NOX2 and G6PD as controls, in neutrophils activated with three different stimuli:

- In PMA-activated neutrophils, PKCglo was the only kinase inhibitor that reduced NADK activity, to a similar extent as PLDi and PLD1i (Figure 4D).
- In neutrophils activated with A23187 or *C. albicans* hyphae, PLDi and PLD1i, but not DPI, G6PDi, PKCglo, Akti nor Rafi, diminished NADK activity (Figure 4E–F).

These results indicate that PLD1 is required for NADK activation, regardless of the stimulus, while PKC regulates NADK only in PMA-stimulated neutrophils. Our data indicate that NADK can be activated through two independent pathways: by classical phosphorylation, or by binding PA or $PI(4,5)P_2$. This is supported by the following observations. (1) In A23187- or *C. albicans*-activated neutrophils, PKC is not required for NADK activity, suggesting that either an unknown kinase phosphorylates NADK or an alternative mechanism operates. (2) PLDi and PLD1i do not inhibit recombinant NADK activity *in vitro* (Supplementary Figure 7E), excluding an off-target effect on the enzyme. (3) PA and $PI(4,5)P_2$ activate recombinant NADK in a cell-free assay (Figure 5). Together, these data indicate that PLD1-derived lipids directly activate NADK, a conclusion that does not depend on the removed panel.

For Figure 5A (lipid dot-blot). This is a lipid-overlay assay, in which immobilised lipids are probed with recombinant protein to test direct binding. The format does not carry molecular-weight markers, and there is no conventional within-assay control for it. We updated the panel to show 2 independent experiments, and uncropped scans for both replicates are provided in the Source Data.

Manuscript text change.

Legend for Figure 5: A. Representative dot blot of the indicated lipids with recombinant NADK. […] A. Representative blot from two independent experiments.

Point 6. *In the abstract the authors reference NADP sustaining glucose metabolism but provide no direct evidence to support this statement. Overall, the limited n number make it very difficult to have confidence in the data interpretation, I do not understand how the authors have been able to perform statistical analysis in this context.*

Response. We thank the reviewer for this comment. We addressed this concern in two ways.

First, we removed the claim from the Abstract. The Abstract now states that PLD1-driven NADK activation supplies the cytosolic $NADP^+$ pool, which is available for reduction to NADPH via the PPP and for consumption by NOX2[5,6]. This framing is supported by two independent studies: Britt et al. 2022[5], who showed by metabolomic tracing that activated neutrophils reroute glucose through a cyclic PPP coupled to the NOX2 burst and NET release, and Amara et al. 2021[6], who showed that diverting glycolytic flux away from the PPP depletes NADPH and suppresses the NOX2-dependent burst.

Second, our data support the revised statement and show where PLD1 acts. We measured NAD^+, $NADP^+$ and NADPH over time (Figure 4A–C; Supplementary Figure 7A–C). Each inhibitor gives a distinct signature. PLD and PLD1 inhibition keep NAD^+ high, preventing the fall that PMA alone causes, and block the rise in $NADP^+$. Both effects point to the same step: NADK, the enzyme that consumes NAD^+ to make $NADP^+$. Thus, NADK blockade by PLD inhibition limits $NADP^+$ supply. DPI, which blocks NOX2, instead lets NADPH build up. G6PDi, which blocks the PPP, lets $NADP^+$ build up due to limited NADPH generation. PLD1 inhibition matches neither downstream pattern, so it acts upstream of both, at the level of $NADP^+$ supply. The ECAR data agree: PLD inhibition lowers the PMA-induced ECAR to a similar extent as G6PDi (Supplementary Figure 6I–J), as expected when a smaller $NADP^+$ pool limits PPP flux.

Third, we agree that the original statistical concern was with an N = 2. In the revised manuscript, biochemical and lysate-based experiments are now N = 4–5 biological donors per condition, with technical replicates averaged so that each plotted point is one donor. These datasets are tested by one-way ANOVA with Bonferroni post-hoc correction, which is appropriate at this replication. This is detailed in our response to Point 3.

Manuscript text change.

Abstract, lines 16–19: PLD1-derived PA and PIP_2 directly bind to and activate NADK, supplying the cytosolic $NADP^+$ pool, which is available for reduction to NADPH via the oxidative pentose phosphate pathway (PPP) and for consumption by NADPH Oxidase 2 (NOX2) during NET formation.

Introduction, lines 58–59: rapidly increasing $NADP^+$ availability, which is reduced to NADPH via the oxidative PPP and consumed by NOX2 during NET formation.

Minor Points

Point M1. *Figure legend 2, the authors state Pi4P when they mean PIP_3.*

Response. We thank the reviewer for catching this error. Figure legend 2 now correctly refers to PIP_3 and we standardized lipid nomenclature throughout the manuscript: we now use PIP_2 (phosphatidylinositol 4,5-bisphosphate), PIP_3 (phosphatidylinositol (3,4,5)-trisphosphate) and PI4P (phosphatidylinositol 4-phosphate) as the standard abbreviations in the main text, reserving the systematic bracket form for introductory or defining use and for naming specific molecular species (for example $PI(4,5)P_2$ (18:1/18:1)).

Manuscript text change.

Legend for Figure 2: PLD1 (green) and PI4P (magenta) top panels, PLD1 (green) and PIP_2 (magenta) middle panels and PLD1 (green) and PIP_3 (magenta) bottom panels.

Point M2. *Insufficient detail provided re use of $CD34^+$ stem cells and PLD1 CRISPR knockdown.*

We thank the reviewer for this suggestion. We substantially expanded the Methods section for $CD34^+$ haematopoietic stem and progenitor cell (HSPC) differentiation and PLD1 CRISPR-Cas9 editing. The additions include the stage-specific cytokine schedule, electroporation parameters, and editing-efficiency readout. These protocols build on published methods[15–18].

Manuscript text change.

Methods, lines 819–853: $CD34^+$ HSPC isolation and neutrophil-like differentiation. $CD34^+$ haematopoietic stem and progenitor cells were isolated from human buffy coats by density-gradient centrifugation over Histopaque-1077 (400 xg, 35 min, no brake) to obtain PBMCs, followed by red-cell lysis in ammonium chloride buffer (10 min, 4 °C) and magnetic-activated cell sorting (MACS) using the CD34 MicroBead Ultra-Pure Kit (Miltenyi Biotec). $CD34^+$ cells were seeded at 100,000 cells/ml in IMDM, maintained at 5×10^5 cells/ml, and differentiated over 28 days using a stage-specific cytokine regimen adapted from published protocols[15–18]. During the expansion phase (Days 0–14), cells were cultured on non-treated polystyrene plates with 10% human serum, SR-1

(1 µM), UM729 (1 µM), SCF (50 ng/mL), FLT3L (50 ng/mL), IL-3 (10 ng/mL), TPO (20 ng/mL) and cholesterin (10 µg/mL). From Day 14, serum was reduced to 5%, SR-1, UM729, TPO and cholesterin were removed, and cells were transferred to fibronectin-coated plates (StemCell Technologies). To drive maturation, IL-6 (10 ng/mL) and G-CSF (10 ng/mL) were introduced on Day 14 and maintained continuously; SCF and FLT3L were halved to 25 ng/mL on Days 17–18 and then withdrawn; IL-3 was maintained at 10 ng/mL until Days 17–18 before withdrawal; GM-CSF was introduced at 10 ng/mL on Day 14, decreased to 5 ng/mL on Days 17–18, then removed. To promote terminal neutrophilic maturation, DHT (100 nM) and nicotinamide (1 mM) were added from Day 24 onwards. Donor sex and gender were not recorded at collection, either by self-report or by assignment, and cannot be linked to individual samples. Sex- and gender-disaggregated analyses were therefore not possible, and none were performed. Our findings do not apply to only one sex or gender, and the cellular and biochemical mechanisms we describe are not expected to be sex or gender specific.

CRISPR-Cas9 mutagenesis of PLD1. For PLD1 knockout, $CD34^{+}$ HSPCs were expanded for 10 days prior to electroporation. Ribonucleoprotein (RNP) complexes were assembled by combining Cas9-GFP protein (IDT, Cat. No. 10008161; 5 µg per 10^{6} cells) with a sgRNA targeting PLD1 exon 14 (5'-GAGCACAGACTCACAGACGT-3', chr3q26.31) or a non-targeting sgRNA at a 1:5 molar ratio (Cas9:sgRNA), incubated at room temperature for 10–15 min. Cells were resuspended in Opti-MEM at 2×10^{7} cells/ml, mixed with the RNP complex, and electroporated using a NEPA21 electroporator (Nepagene) with the following settings: Poring pulse, 175 V, 5 ms pulse length, 50 ms interval, 2 pulses, 10% decay, positive polarity; Transfer pulse, 20 V, 50 ms pulse length, 50 ms interval, 5 pulses, 40% decay, ± polarity. Twenty-four hours post-electroporation, GFP^{+} cells were isolated by FACS and returned to the stage-specific differentiation culture. Editing efficiency was assessed at Days 21–28 by extracting genomic DNA (Qiagen DNeasy Kit), PCR-amplifying the target locus (Q5 High-Fidelity Polymerase, NEB) and Sanger sequencing (Eurofins Genomics); indel frequency was quantified using the TIDE algorithm.

Response to Reviewer 3

Reviewer comment. *This manuscript examines how PLD1 regulates NET formation by linking phospholipid signaling to NADK-dependent NADP production and downstream NOX2 activity. The overall question is interesting, and the study has a number of strengths, particularly the combination of genetic perturbation, lipid imaging, and metabolite measurements. The central model is also plausible. My main concerns are with how some of the data are interpreted and presented. In several places, the manuscript overstates what the inhibitor and metabolic data show. There are also a number of internal inconsistencies in the figure citations and some claims from microscopy that would benefit from quantification.*

Response. We thank the reviewer for the constructive assessment and for recognising the strengths of our combined genetic perturbation, lipid imaging and metabolite measurement approach. We addressed the concerns regarding overstated interpretation, internal inconsistencies and unquantified microscopy through comprehensive revision of the text, figures and analyses.

Point 1. The manuscript repeatedly conflates glucose metabolism, glycolysis, and PPP activity.

This is the biggest issue in the paper. These terms are used too loosely and at times interchangeably, especially in the NADK/G6PD/ECAR sections. For example, the manuscript states that conversion of NAD to NADP increases NADP availability "to sustain glucose metabolism and NOX2-

dependent ROS production," and later argues that PLD inhibition attenuates "glucose metabolism" based on ECAR. That is too broad. NADK is directly relevant to the NADP(H) pool, oxidative PPP flux, and NOX2 function. That is not the same as saying it sustains glycolysis or "glucose metabolism" more generally. Likewise, reduced ECAR after G6PDi or PLD inhibition does not by itself show direct inhibition of glycolysis. In activated neutrophils, this could also reflect disruption of PPP cycling and a broader reduction in activation-associated glucose flux. The authors need to clean this up throughout the manuscript.

We agree and thank the reviewer for this important point. We revised the terminology throughout to distinguish them clearly.

- We no longer use "glucose metabolism" as a specific claim. Where the overall concept is meant, we now specify the process involved, whether glycolysis, oxidative PPP flux, or the NADP(H) pool. The phrase "glucose metabolism" has been removed from the manuscript.
- Vague statements such as "PLD1 inhibition attenuates glucose metabolism" were replaced with mechanistically specific wording, for example, "PLD1 inhibition reduces activation-associated extracellular acidification consistent with impaired oxidative PPP flux".
- ECAR is now interpreted narrowly. Reduced ECAR following PLD1 or G6PDi is described as reduced activation-associated extracellular acidification consistent with impaired oxidative pentose phosphate pathway (PPP) flux. We state explicitly that ECAR does not by itself establish direct inhibition of glycolysis.
- The Abstract, Results and Discussion were revised accordingly. The Discussion limitations paragraph now states plainly that we did not measure glycolytic or PPP flux directly, and that our metabolic conclusions rest on NADP(H) pool measurements and on ECAR interpreted as activation-associated acidification.

The revised text now addresses the requested rigour.

Manuscript text change.

Introduction, lines 34–36: Sustained ROS production requires continuous regeneration of NADPH from $NADP^+$, principally via the oxidative pentose phosphate pathway (PPP)[5,19].

Results, lines 183–184: This NOX2-driven ROS production is fuelled by NADPH regenerated through the oxidative PPP (Supplementary Figure 6A)[5,19].

Results, lines 245–254: We next examined how PLD1 inhibition affects extracellular acidification rate (ECAR). As expected, G6PDi reduced the ECAR response to PMA or A23187, as did PLD and PLD1 inhibition (Supplementary Figure 6I–J). We interpret this as a reduction in activation-associated extracellular acidification, compatible with impaired PPP cycling or a broader reduction in activation-associated glucose flux; ECAR alone does not establish direct inhibition of glycolysis. Critically, the NAD^+/$NADP^+$/NADPH patterns under PLD inhibition differed markedly from those induced by G6PDi or DPI (Figure 4A–C; Supplementary Figure 7A–C), placing PLD1 upstream of NADK-mediated $NADP^+$ synthesis rather than within the PPP or at NOX2. By reducing NADP(H) supply to NOX2, PLD1 inhibition therefore accounts for both the fall in ECAR and the block in NET release.

Discussion, lines 380–384: Our study has limitations. We have not measured glycolytic or PPP flux directly; our metabolic conclusions rest on the NADP(H) pool measurements and on ECAR interpreted as activation-associated acidification. More generally, inhibitor-based conclusions have intrinsic limitations; where possible, pharmacological perturbations were corroborated by PLD1-CRISPR genetic loss-of-function, and instances in which pharmacological and genetic results diverge are noted as such.

Abstract, lines 16–19: PLD1-derived PA and PIP_2 directly bind to and activate NADK, supplying the cytosolic $NADP^+$ pool, which is available for reduction to NADPH via the oxidative pentose phosphate pathway (PPP) and for consumption by NADPH Oxidase 2 (NOX2) during NET formation.

Introduction, lines 58–59: rapidly increasing $NADP^+$ availability, which is reduced to NADPH via the oxidative PPP and consumed by NOX2 during NET formation.

Results, lines 221–226: In unstimulated neutrophils, NAD^+, $NADP^+$ and NADPH concentrations remained stable, but upon PMA stimulation, NAD^+ rapidly decreased while $NADP^+$ increased within 10 minutes (Supplementary Figure 7A–C). NADPH levels remained relatively stable, consistent with continued reduction of $NADP^+$ to NADPH via the oxidative PPP and its recycling back to $NADP^+$ by NOX2. Supplementary Figure 7D schematises this cycle, in which NAD kinase phosphorylates NAD^+ to $NADP^+$[7,8].

Legend for Supplementary Figure 6: Glycolysis and the pentose phosphate pathway (PPP) convert $NADP^+$ to NADPH, the substrate of NOX2 to mediate ROS production.

Legend for Supplementary Figure 7: $NADP^+$ is reduced to NADPH via the oxidative pentose phosphate pathway (PPP). PLD inhibitors act upstream of the PPP and NOX2 activity by regulating NADK activity.

Discussion, lines 322–323: Notably, this $NADP^+/NAD^+$ shift reflected impaired NADK activity rather than altered oxidative-PPP flux or NOX2 activity.

Point 2. Some inhibitor-based conclusions are stronger than the data support.

There are several places where the text makes exclusionary pathway statements that do not seem fully supported by the experiments shown. For example, the statement that PIP2 induction depends on PLD1 and a calcium-independent PKC isoform rather than ROS, glucose metabolism, or Akt signaling is confusing as written. In that section, the inhibitor set appears to address ROS/flavoprotein activity, Akt, and PKC isoforms, but not glucose metabolism directly. Similarly, the claim that PMA-induced PLD activity is independent of ROS seems stronger than warranted based on the apparent magnitude of the DPI effect. These statements should either be toned down or supported more clearly.

Response. We agree and thank the reviewer for this careful assessment. We made the following changes:

- Each cross-pathway claim now distinguishes between directly tested and inferred pathways. The sentence "PIP_2 induction depends on PLD1 and a Ca^{2+}-independent PKC isoform rather than ROS, glucose metabolism, or Akt signalling" was replaced with: "PIP_2 induction was reduced by inhibition of PLD1 and Ca^{2+}-independent PKC isoforms, but was not significantly affected by Akt, NOX2 inhibition or oxidative PPP inhibition (G6PDi, a glucose-6-phosphate dehydrogenase inhibitor[5]) after PMA stimulation."
- We also found that the A23187 $PI(4,5)P_2$ response differs from PMA. Although PLD and PLD1 inhibition reduce $PI(4,5)P_2$ under both stimuli, the pan-PKC inhibitor PKCglo does not significantly reduce the A23187 response (Supplementary Figure 4C), whereas it partially reduces the PMA response (Figure 2I). The text no longer describes the A23187 data as mirroring PMA across the inhibitor panel; this stimulus-specific PKC dependence is consistent with PKC contributing only under PMA, while PLD1 is required across stimuli.
- We reassessed the claim that PMA-induced PLD activity is reactive oxygen species (ROS)-independent. Quantified across 4 donors, DPI produces only a modest reduction. The respective section is now corrected.

- The Discussion now acknowledges the limitations of inhibitor-based conclusions and indicates where PLD1-CRISPR genetic loss-of-function corroborates the pharmacology and where it does not.

Manuscript text change.

Results, lines 137–154: We next assessed downstream PI-derived phosphoinositides, phosphatidylinositol-4-monophosphate (PI4P), phosphatidylinositol 4,5-bisphosphate (PIP_2) and phosphatidylinositol (3,4,5)-trisphosphate (PIP_3) (Supplementary Figure 3E)[20,21]. PMA triggered PI4P and PIP_2 accumulation in the cytoplasm and nucleoplasm, which was abrogated upon PLD1 inhibition (Figure 2E). Quantification of the mean fluorescence intensity confirmed that PLD1i and PKCglo, but not DPI, reduced PI4P and PIP_2 (Figure 2F–G). PIP_3 gave a signal close to background by IF, consistent with its highly transient nature (Figure 2E,H). Using a PIP_2 mass ELISA, we confirmed that PMA markedly increased PIP_2 concentrations, whereas PLD and PLD1 inhibitors reduced this response (Figure 2I). Of note, PKCglo, but not the PKCα/β-selective inhibitor Gö6976 (PKCab), Akt-1/2 inhibitor (Akti) or DPI, reduced PIP_2 production, comparable to PLDi and PLD1i. This indicates that PIP_2 induction was reduced by inhibition of PLD1 and Ca^{2+}-independent PKC isoforms, but was not significantly affected by Akt, NOX2 inhibition or oxidative PPP inhibition (G6PDi, a glucose-6-phosphate dehydrogenase inhibitor[5]) after PMA stimulation. In contrast, when the neutrophils were stimulated with A23187, PLD and PLD1 inhibitors reduced A23187-induced PIP_2, whereas PKCglo, PKCab, Akti, DPI and G6PDi had no significant effect (Supplementary Figure 4C); imaging of the corresponding conditions showed the same pattern (Supplementary Figure 4D). These data suggest that the PKC contribution to PIP_2 generation is PMA-specific, whereas PLD1 is required for both stimuli.

Results, lines 105–114: Consistently, PLD activity increased upon PMA activation, as measured with a fluorogenic substrate (Supplementary Figure 3A). PMA elicits PKC and NOX2 activity to trigger NETosis[22]. Notably, the PKC inhibitor PKCglo significantly reduced PLD activity. DPI had a statistically significant but modest effect on PLD activity compared with the PLD inhibitors, indicating that ROS contribution is minor (Supplementary Figure 3A). A23187 and *C. albicans* hyphae[5] also robustly induced neutrophil PLD activity largely independently of ROS production. As expected, ML299 (PLDi, PLD1 and PLD2 inhibitor) and PLD1i blocked increases in PLD activity by all stimuli (Supplementary Figure 3A). Since PLD liberates choline from PC, as expected, the concentration of choline (Supplementary Figure 3B) mirrored the readout for PLD activity (Supplementary Figure 3A).

Point 3. There are multiple internal inconsistencies in figure citation and figure/model alignment.

The paper needs a careful pass here. The inhibitors shown in the model figure should match what is actually tested in the supporting data. I also do not see DPI used in the figure cited to support the claim around line 164. Later, line 322 appears to call out Fig. 4G, but the relevant data look to be in Fig. 4E–F. These are not major issues individually, but there are enough of them that they become distracting.

We thank the reviewer for identifying these inconsistencies. We corrected the figure-citation accuracy.

- Every in-text figure-panel citation was checked against the corresponding figure file. The DPI citation now references Supplementary Figure 3A and the Figure 4G callout is now updated to Figure 4E–F.
- Our schematic panels (Figure 3A and Supplementary Figure 6A) depict the NOX2/ROS-generating pathway and do not themselves show the inhibitor panel; the inhibitors referred to

in the text are those tested in the corresponding data figures. We verified that every inhibitor named in the text matches the supporting data.

Manuscript text change.

Results, lines 105–114: Consistently, PLD activity increased upon PMA activation, as measured with a fluorogenic substrate (Supplementary Figure 3A). PMA elicits PKC and NOX2 activity to trigger NETosis[22]. Notably, the PKC inhibitor PKCglo significantly reduced PLD activity. DPI had a statistically significant but modest effect on PLD activity compared with the PLD inhibitors, indicating that ROS contribution is minor (Supplementary Figure 3A). A23187 and *C. albicans* hyphae[5] also robustly induced neutrophil PLD activity largely independently of ROS production. As expected, ML299 (PLDi, PLD1 and PLD2 inhibitor) and PLD1i blocked increases in PLD activity by all stimuli (Supplementary Figure 3A). Since PLD liberates choline from PC, as expected, the concentration of choline (Supplementary Figure 3B) mirrored the readout for PLD activity (Supplementary Figure 3A).

Results, lines 241–242: PLD inhibition also reduced NADK activity following A23187 stimulation or *C. albicans* hyphae exposure (Figure 4E–F).

Point 4. The microscopy-based claims need more quantification.

There are multiple points where representative images are used to support changes in localization or abundance without quantitative analysis. This is particularly relevant where the manuscript discusses PLD1 redistribution and relocalization of lipid species or associated proteins. If the conclusion depends on altered localization or enrichment, that should be quantified.

Response. We thank the reviewer for this suggestion. We added quantitative analyses to support microscopy-based claims. Cytoplasmic PLD1 is now quantified per cell (revised Supplementary Figure 3D): in unstimulated neutrophils PLD1 is largely confined to the plasma membrane, whereas upon PMA stimulation cytoplasmic PLD1 intensity rises. For PI(4)P and $PI(4,5)P_2$, per-cell quantification of mean fluorescence intensity now backs the text claims (revised Figure 2F–G), with ELISA corroboration for $PI(4,5)P_2$. $PI(3,4,5)P_3$ gave only a signal close to background by immunofluorescence, and the manuscript now shows only this per-cell mean fluorescence intensity data for $PI(3,4,5)P_3$ (revised Figure 2H), which we treat as a background/negative reference rather than interpreting; the corroborating ELISA measurement (values are all below the assay limit of detection) is provided in this response but is not shown in the manuscript. The Methods have been updated to include the single-cell image-quantification methods.

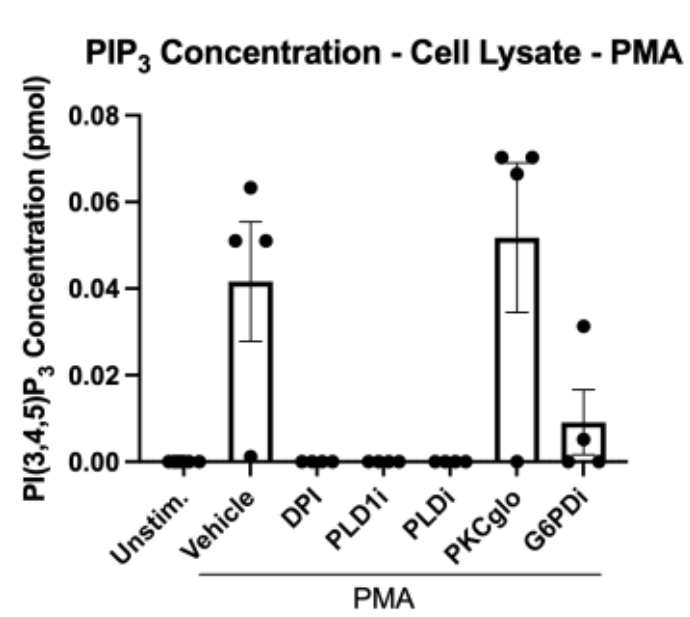

Legend: PIP_3 concentrations in lysates of neutrophils treated with the indicated inhibitors before 30 min PMA activation. Data shown as mean ± s.e.m., N = 4 donors. We plotted the mean of two technical replicates for each donor.

Manuscript text change.

Results, lines 115–122: Upon cellular activation, PLD1 relocates to specific cellular membranes to produce PA locally[23]. In PMA-stimulated neutrophils, PLD1 redistributes from the plasma membrane to the cytoplasm 30 min after stimulation (Supplementary Figure 3C–D). PLD1 intensity quantification confirmed that the cytoplasmic signal increased after PMA stimulation and was reduced by PLD1 and PKC inhibition (PKCglo), but not by DPI (Supplementary Figure 3D). PLD1 was not detectable 180 min after stimulation, likely because endomembranes disassemble during NETosis[24]. Notably, PLD1 did not colocalise with NETs, indicating that its activity is intracellular prior to chromatin decondensation (Supplementary Figure 3C).

Results, lines 137–154: We next assessed downstream PI-derived phosphoinositides, phosphatidylinositol-4-monophosphate (PI4P), phosphatidylinositol 4,5-bisphosphate (PIP_2) and phosphatidylinositol (3,4,5)-trisphosphate (PIP_3) (Supplementary Figure 3E)[20,21]. PMA triggered PI4P and PIP_2 accumulation in the cytoplasm and nucleoplasm, which was abrogated upon PLD1 inhibition (Figure 2E). Quantification of the mean fluorescence intensity confirmed that PLD1i and PKCglo, but not DPI, reduced PI4P and PIP_2 (Figure 2F–G). PIP_3 gave a signal close to background by IF, consistent with its highly transient nature (Figure 2E,H). Using a PIP_2 mass ELISA, we confirmed that PMA markedly increased PIP_2 concentrations, whereas PLD and PLD1 inhibitors reduced this response (Figure 2I). Of note, PKCglo, but not the PKCα/β-selective inhibitor Gö6976 (PKCab), Akt-1/2 inhibitor (Akti) or DPI, reduced PIP_2 production, comparable to PLDi and PLD1i. This indicates that PIP_2 induction was reduced by inhibition of PLD1 and Ca^{2+}-independent PKC isoforms, but was not significantly affected by Akt, NOX2 inhibition or oxidative PPP inhibition (G6PDi, a glucose-6-phosphate dehydrogenase inhibitor[5]) after PMA stimulation. In contrast, when the neutrophils were stimulated with A23187, PLD and PLD1 inhibitors reduced A23187-induced PIP_2, whereas PKCglo, PKCab, Akti, DPI and G6PDi had no significant effect (Supplementary Figure 4C); imaging of the corresponding conditions showed the same pattern (Supplementary Figure 4D). These data suggest that the PKC contribution to PIP_2 generation is PMA-specific, whereas PLD1 is required for both stimuli.

Methods, lines 761–782: Images were acquired on Leica confocal microscopes and processed with LAS-X. PIP_2 panels were acquired with an HC PL APO 63×/1.32 oil-immersion objective at 2048×2048 pixels, whereas PI4P and PIP_3 panels were acquired at 1024×1024 pixels on an SP8 AOBS with a 100× oil objective. For each target, all conditions were imaged in the same run and analysed with a fully scripted pipeline in Python (scikit-image v0.26.0)[13]. Each channel was recorded across all Z planes, and the stack was combined by sum-intensity projection before measurement. Background was removed in two stages: a per-slice rolling-ball estimate per channel, followed by a per-cell local correction subtracting the median intensity of a surrounding annular ("donut") ring, excluding neighbouring cells and image borders, with an image-wide non-cell median as a fall-back. Cells were segmented by a marker-controlled watershed[14]; the DAPI-stained nucleus of each cell served as the starting marker (Gaussian smoothing, Li thresholding and morphological closing, which merges the lobes of a single polymorphonuclear nucleus without joining adjacent cells), and the cell footprint was defined by Li (or, as a fall-back, triangle) thresholding of the combined PLD1-and-phosphoinositide signal. For each cell the nucleus was excluded, and intensities were measured for PLD1 and the phosphoinositide channel; we report the mean fluorescence intensity. For PLD1, the rim (0.45 µm wide, a plasma-membrane proxy) was additionally excluded and the mean cytoplasmic fluorescence intensity measured in the interior compartment. Cells touching the image border, or with a cross-sectional area outside 20–2000 μm^2, were excluded, and the number of cells retained per condition per donor is specified as a range in each figure legend (two independent donors). For each target, the pooled single-cell distributions were compared across conditions by one-way ANOVA with Bonferroni's multiple comparisons test.

Point 5. The NADK phosphorylation measurements are not described clearly enough.

I was confused by how NADK phosphorylation was specifically assessed. As written, the phos-tag SDS-PAGE description is not clear enough to understand how this was quantified for NADK specifically rather than phosphoproteins more broadly. If the authors ran a phos-tag gel and then immunoblotted for NADK, that should be stated explicitly in the Methods and figure legend. If that is not what was done, then the wording around this result needs to be revised.

The reviewer is right that the phos-tag quantification was not described clearly. On revisiting it, we found the assay could no longer be reproduced, and we have removed the panel.

The proprietary formulation of the SuperSep Phos-tag precast gels (FUJIFILM Wako) changed since the experiment reported in the first submission of this manuscript. With the available gels, we failed to obtain interpretable separation. Furthermore, delivery lag times for these gels increased to several months, making further optimisation difficult. We therefore decided to remove the Phos-tag panel and all statements derived from it across the manuscript.

This does not affect the manuscript's conclusions. We tested the mechanisms driving NADK phosphorylation in neutrophils, since it was reported that PKC and Akt phosphorylate NADK in other cell types[7,8]. We addressed the requirement of phosphorylation to activate NADK with enzymatic activity assays, where we tested inhibitors of PKC, Akt and Raf, as well as NOX2 and G6PD as controls, in neutrophils activated with three different stimuli:

- In PMA-activated neutrophils, PKCglo was the only kinase inhibitor that reduced NADK activity, to a similar extent as PLDi and PLD1i (Figure 4D).
- In neutrophils activated with A23187 or *C. albicans* hyphae, PLDi and PLD1i, but not DPI, G6PDi, PKCglo, Akti nor Rafi, diminished NADK activity (Figure 4E–F).

These results indicate that PLD1 is required for NADK activation, regardless of the stimulus, while PKC regulates NADK only in PMA-stimulated neutrophils. Our data indicate that NADK can be activated through two independent pathways: by classical phosphorylation, or by binding PA or $PI(4,5)P_2$. This is supported by the following observations. (1) In A23187- or *C. albicans*-activated neutrophils, PKC is not required for NADK activity, suggesting that either an unknown kinase phosphorylates NADK or an alternative mechanism operates. (2) PLDi and PLD1i do not inhibit recombinant NADK activity *in vitro* (Supplementary Figure 7E), excluding an off-target effect on the enzyme. (3) PA and $PI(4,5)P_2$ activate recombinant NADK in a cell-free assay (Figure 5). Together, these data indicate that PLD1-derived lipids directly activate NADK, a conclusion that does not depend on the removed panel.

We revised the Results and figure legends to remove the phosphorylation claim and to frame NAD kinase (NADK) activation in terms of the quantitative, multi-stimulus activity assays (N = 5 donors for PMA; N = 4 for A23187 and *C. albicans* hyphae).

Manuscript text change.

Results, lines 240–244: Consistent with a block in $NADP^+$ synthesis, PLDi and PLD1i, but not DPI or G6PDi, reduced NADK activity after PMA stimulation (Figure 4D). PLD inhibition also reduced NADK activity following A23187 stimulation or *C. albicans hyphae* exposure (Figure 4E–F). Notably, PLD inhibitors did not affect the activity of recombinant NADK, excluding the possibility that PLDi or PLD1i had an off-target effect on this enzyme (Supplementary Figure 7E).

Results, lines 255–263: Phosphorylation of NADK by kinases such as Akt, Raf or PKC enhances its activity[7,8,25]. Neither Akt (Akti) nor Raf (GW5074, Rafi) inhibitors influenced NADK activity

in lysates from PMA-stimulated neutrophils (Figure 4D). However, PKCglo, but not PKCab, decreased NADK activity (Figure 4D), suggesting that a Ca^{2+}-independent PKC isoform regulates NADK activity during PMA stimulation. PKCglo also reduced PMA-induced PLD activity to a similar extent as the PLD inhibitors themselves (Supplementary Figure 3A), so its effect on NADK may be indirect. Interestingly, neither PKCglo, Akti nor Rafi affected NADK activity after A23187 or *C. albicans* hyphae stimulation, and only PLD and PLD1 inhibition did so (Figure 4D–F). This implies that PLD-derived lipids can activate NADK independently of its phosphorylation.

Discussion, lines 325–327: Instead, we found that PLD1 regulates cellular NADK activity across multiple stimuli, establishing NADK as the downstream metabolic effector linking PLD1-derived lipids to $NADP^+$ production.

Point 6. The ECAR data should be interpreted more narrowly.

Related to point 1, I do not think the ECAR data support the broad conclusion that PLD inhibition attenuates "glucose metabolism." At most, these data show that PLD1 inhibition, like G6PD inhibition, reduces activation-associated extracellular acidification. That is compatible with impaired PPP-linked glucose flux and/or PPP cycling, but it does not establish direct inhibition of glycolysis. The text should be revised accordingly.

We thank the reviewer for this point. We revised the ECAR text as suggested. We now state that PLD1i and G6PDi reduce activation-associated extracellular acidification, which is compatible with impaired PPP cycling or a broader reduction in activation-associated glucose flux, but does not establish direct inhibition of glycolysis.

Critically, our conclusion that PLD1 acts upstream of the PPP does not rest only on ECAR. It rests on direct NAD^+/$NADP^+$/NADPH measurements, in which two control inhibitors define the reference signatures: the NOX2 inhibitor DPI and the PPP inhibitor G6PDi (Figure 4A–C; Supplementary Figure 7A–C). On PMA activation, $NADP^+$ rises and NAD^+ falls. PLD and PLD1 inhibition prevent both: NAD^+ stays high and the $NADP^+$ rise is blocked. Both effects point to the same step: NADK, the enzyme that consumes NAD^+ to make $NADP^+$. By blocking NOX2, DPI instead lets NADPH accumulate. By blocking the PPP, G6PDi lets $NADP^+$ accumulate as a result of limited NADPH regeneration. PLD1 inhibition matches neither control pattern, so it acts upstream of both, at $NADP^+$ supply rather than within the PPP or at NOX2.

Manuscript text change.

Results, lines 245–254: We next examined how PLD1 inhibition affects extracellular acidification rate (ECAR). As expected, G6PDi reduced the ECAR response to PMA or A23187, as did PLD and PLD1 inhibition (Supplementary Figure 6I–J). We interpret this as a reduction in activation-associated extracellular acidification, compatible with impaired PPP cycling or a broader reduction in activation-associated glucose flux; ECAR alone does not establish direct inhibition of glycolysis. Critically, the NAD^+/$NADP^+$/NADPH patterns under PLD inhibition differed markedly from those induced by G6PDi or DPI (Figure 4A–C; Supplementary Figure 7A–C), placing PLD1 upstream of NADK-mediated $NADP^+$ synthesis rather than within the PPP or at NOX2. By reducing NADP(H) supply to NOX2, PLD1 inhibition therefore accounts for both the fall in ECAR and the block in NET release.

Discussion lines 380–382: Our study has limitations. We have not measured glycolytic or PPP flux directly; our metabolic conclusions rest on the NADP(H) pool measurements and on ECAR interpreted as activation-associated acidification.

Point 7. Some of the supplemental presentation feels redundant.

I was not sure what Supplementary Fig. 7A–C adds beyond Fig. 4A–C other than the inclusion of additional time points. If the purpose is simply to show temporal consistency, that should be stated more clearly. As presented, it feels repetitive.

Response. We thank the reviewer for this important point. We agree the two panels overlap and have clarified the distinct purpose of each.

Figure 4A–C shows the 30-min timepoint, when ROS activity peaks. Various assays, including NADK activity, $PI(4,5)P_2$ and the lipidomic measurements, were made at this timepoint. Keeping the 30-min nicotinamide comparison in the main figure aligns it with those datasets.

Supplementary Figure 7A–C is the temporal extension of this snapshot. It serves two purposes the main figure cannot. First, it shows that the inhibitor phenotype is sustained across activation, not confined to a single timepoint: PLD and PLD1 inhibition blunted the $NADP^+$ rise and retained NAD^+ throughout, whereas DPI blunted $NADP^+$ without retaining NAD^+. Second, it shows that the nicotinamide pools are dynamically tuned rather than static. This is clearest for $NADP^+$, the pool that supplies NADPH to NOX2, which PLD1 inhibition keeps low at every timepoint. This also addresses Reviewer 1's Point 6, on whether $NADP^+$ production is dynamically tuned during activation. We have kept the full time course as a reference for the reader.

We have revised the Results and retitled the Supplementary Figure 7 legend as a temporal extension.

Manuscript text change.

Results, lines 216–220: To determine how PLD1 regulates this nicotinamide metabolic cycle, we quantified NAD^+, $NADP^+$ and NADPH at 10, 30 (this timepoint is highlighted in Figures 4A–C) and 60 minutes after PMA stimulation in the presence or absence of NOX2, PPP or PLD inhibitors (Supplementary Figure 7A–C).

Legend for Supplementary Figure 7: Temporal extension of Figure 4A–C. NAD^+ (A), $NADP^+$ (B) and NADPH (C) concentrations in neutrophil lysates at 10, 30 and 60 min after stimulation, in the presence of NOX2 (DPI), PPP (G6PDi) and PLD inhibitors. The 30-min time point corresponds to the snapshot shown in Figure 4A–C.

We sincerely thank the reviewers for their thoughtful and constructive feedback. We believe the revised manuscript is substantially strengthened by the additional experiments and the careful re-interpretation of the data.

Bibliography

1. Thakur, R., Naik, A., Panda, A. & Raghu, P. Regulation of Membrane Turnover by Phosphatidic Acid: Cellular Functions and Disease Implications. *Front. Cell Dev. Biol.* **7**, 83 (2019).
2. Nelson, R. K. & Frohman, M. A. Physiological and pathophysiological roles for phospholipase D. *J. Lipid Res.* **56**, 2229–2237 (2015).
3. Kenny, E. F. *et al.* Diverse stimuli engage different neutrophil extracellular trap pathways. *eLife* **6**, e24437 (2017).
4. Tilley, D. O. *et al.* Histone H3 clipping is a novel signature of human neutrophil extracellular traps. *eLife* **11**, e68283 (2022).

5. Britt, E. C. *et al.* Switching to the cyclic pentose phosphate pathway powers the oxidative burst in activated neutrophils. *Nat. Metab.* **4**, 389–403 (2022).
6. Amara, N. *et al.* Selective activation of PFKL suppresses the phagocytic oxidative burst. *Cell* **184**, 4480–4494 (2021).
7. Hoxhaj, G. *et al.* Direct stimulation of NADP+ synthesis through Akt-mediated phosphorylation of NAD kinase. *Science* **363**, 1088–1092 (2019).
8. Schild, T. *et al.* NADK is activated by oncogenic signaling to sustain pancreatic ductal adenocarcinoma. *Cell Rep.* **35**, (2021).
9. Karathanassis, D. *et al.* Binding of the PX domain of p47(phox) to phosphatidylinositol 3,4-bisphosphate and phosphatidic acid is masked by an intramolecular interaction. *EMBO J.* **21**, 5057–5068 (2002).
10. Cockcroft, S. & Raghu, P. Topological organisation of the phosphatidylinositol 4,5-bisphosphate–phospholipase C resynthesis cycle: PITPs bridge the ER–PM gap. *Biochem. J.* **473**, 4289–4310 (2016).
11. Jenkins, G. H., Fisette, P. L. & Anderson, R. A. Type I phosphatidylinositol 4-phosphate 5-kinase isoforms are specifically stimulated by phosphatidic acid. *J. Biol. Chem.* **269**, 11547–11554 (1994).
12. Honda, A. *et al.* Phosphatidylinositol 4-Phosphate 5-Kinase α Is a Downstream Effector of the Small G Protein ARF6 in Membrane Ruffle Formation. *Cell* **99**, 521–532 (1999).
13. van der Walt, S. *et al.* scikit-image: image processing in Python. *PeerJ* **2**, e453 (2014).
14. Beucher, S. & Meyer, F. The morphological approach to segmentation: the watershed transformation. in *Mathematical Morphology in Image Processing* (ed. Dougherty, E. R.) 433–481 (Marcel Dekker, New York, 1993).
15. Nasri, M. *et al.* CRISPR-Cas9n-mediated ELANE promoter editing for gene therapy of severe congenital neutropenia. *Mol. Ther. J. Am. Soc. Gene Ther.* **32**, 1628–1642 (2024).
16. Jie, Z. *et al.* Large-scale ex vivo generation of human neutrophils from cord blood CD34+ cells. *PloS One* **12**, e0180832 (2017).
17. Kuhikar, R. *et al.* Neutrophils generated in vitro from hematopoietic stem cells isolated from apheresis samples and umbilical cord blood form neutrophil extracellular traps. *Stem Cell Res.* **50**, 102150 (2021).
18. Naveh, C. A. *et al.* Neutrophils cultured ex vivo from CD34+ stem cells are immature and genetically tractable. *J. Transl. Med.* **22**, 526 (2024).
19. Burn, G. L., Foti, A., Marsman, G., Patel, D. F. & Zychlinsky, A. The Neutrophil. *Immunity* **54**, 1377–1391 (2021).
20. Moritz, A., De Graan, P. N., Gispen, W. H. & Wirtz, K. W. Phosphatidic acid is a specific activator of phosphatidylinositol-4-phosphate kinase. *J. Biol. Chem.* **267**, 7207–7210 (1992).
21. Roach, A. N. *et al.* Phosphatidic acid regulation of PIPKI is critical for actin cytoskeletal reorganization. *J. Lipid Res.* **53**, 2598–2609 (2012).
22. Hakkim, A. *et al.* Activation of the Raf-MEK-ERK pathway is required for neutrophil extracellular trap formation. *Nat. Chem. Biol.* **7**, 75–77 (2011).
23. Tei, R. & Baskin, J. M. Click chemistry and optogenetic approaches to visualize and manipulate phosphatidic acid signaling. *J. Biol. Chem.* **298**, 101810 (2022).

24. Thiam, H. R. *et al.* NETosis proceeds by cytoskeleton and endomembrane disassembly and PAD4-mediated chromatin decondensation and nuclear envelope rupture. *Proc. Natl. Acad. Sci. U. S. A.* **117**, 7326–7337 (2020).
25. Rabani, R., Cossette, C., Graham, F. & Powell, W. S. Protein kinase C activates NAD kinase in human neutrophils. *Free Radic. Biol. Med.* **161**, 50–59 (2020).

Point-by-Point Response to Reviewers

We thank the reviewers for their careful reading of the revised manuscript. Reviewers 1 and 2 raised no further points. Reviewer 3 raised one remaining correction, which we have made. Our response to each reviewer follows.

Response to Reviewer 1

Reviewer comment. *I appreciate the authors` thoughtful responses to my previous comments. The new data, analyses and extensive rewriting have strengthened this interesting manuscript. I have no further comments.*

We thank the reviewer for their positive assessment, and for the comments made during the earlier rounds of review, which substantially strengthened the manuscript.

Response to Reviewer 2

Reviewer comment. *The authors have undertaken a significant program of work to expand the experimental repeats increasing confidence in the reported results. They have also provided text changes to ensure that they do not over interpret the results provided and removed data sets where for logistic reasons further experimental repeats were not feasible. Relevant methodology text has been provided/expanded as requested. I am happy these revisions have addressed the concerns raised at first review.*

We thank the reviewer for their assessment and for the guidance on experimental replication, data presentation and methodological reporting, which improved the rigour of the study.

Response to Reviewer 3

Reviewer comment. *The authors have carefully addressed my previous concerns. The revised manuscript uses more precise metabolic terminology, appropriately narrows the interpretation of the ECAR and inhibitor experiments, corrects the figure inconsistencies, and provides additional quantitative support for the microscopy-based conclusions. The removal of the unclear NADK phosphorylation experiment and clarification of the supplemental figures also improve the manuscript.*

We thank the reviewer for their positive assessment of the revised manuscript, and for the detailed comments in the earlier rounds that led to the clarification of the metabolic terminology and the narrowing of the ECAR and inhibitor interpretations. We address the one remaining correction below.

Specific Comments

Point 1. *The Supplementary Figure 6 legend states that glycolysis and the PPP both convert NADP+ to NADPH. Glycolysis does not directly perform this conversion; NADP+ is reduced to NADPH through the oxidative PPP.*

The reviewer is correct, and we have made this correction. Glycolysis does not reduce $NADP^+$; in neutrophils $NADP^+$ is reduced to NADPH by the oxidative pentose phosphate pathway. We have amended both the schematic and its legend. Supplementary Figure 6A now shows NOX2 consuming NADPH to generate $NADP^+$, and a single arrow labelled PPP returning $NADP^+$ to NADPH, with no

contribution from glycolysis depicted. The legend for panel A has been shortened accordingly and no longer refers to glycolysis.

We have also checked every remaining mention of glycolysis in the manuscript to confirm that none implies a direct role in NADPH generation. The remaining references describe extracellular acidification and glycolytic flux.
